# A unified approach to the important protein kinase inhibitor balanol and a proposed analogue

**DOI:** 10.3762/bjoc.9.327

**Published:** 2013-12-19

**Authors:** Tapan Saha, Ratnava Maitra, Shital K Chattopadhyay

**Affiliations:** 1 Department of Chemistry, University of Kalyani, Kalyani - 741235, West Bengal, India

**Keywords:** azepane, balanol, Garner’s aldehyde, PKC inhibitor, ring-closing metathesis

## Abstract

A common approach to the important protein kinase inhibitor (−)-balanol and an azepine-ring-modified balanol derivative has been developed using an efficient fragment coupling protocol which proceeded in good overall yield.

## Introduction

Protein kinase C (PKC) is a family of phospholipid-dependent kinases that phosphorylate serine and threonine residues of a substrate protein by transferring a phosphate group from ATP to the substrate protein [1–3]. This phosphorylation induces conformational changes of the substrate protein leading to initiation of a number of cellular events including signal transduction [4–5]. The human PKC enzyme comprises of a number of isozymes and inappropriate activation of PKC has been linked to a variety of disorders [6–7]. The development of selective PKC inhibitors as novel therapeutics has therefore remained significant [8–14].

**Figure 1:**
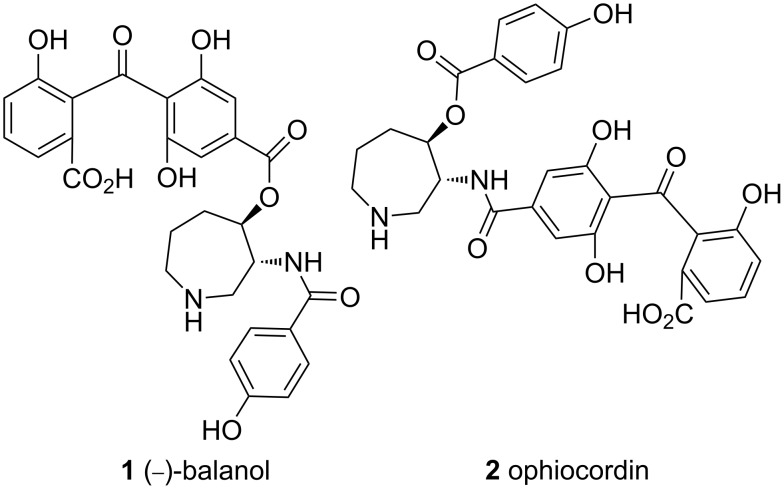
Balanol (**1**) and ophiocordin (**2**).

Balanol ((−)**-1**, Figure 1), a fungal metabolite [15] is known to inhibit a number of PKC isozymes at nanomolar concentrations [16], a finding that has motivated research related to the total- [17–26] or fragment synthesis [27–47] of this important natural product. Based on the information [48–49] that balanol binds to the ATP-docking site of protein kinase, all the three distinct domains present in the natural product such as the benzophenone core [50–52], the azepine core [53–59] and the *p*-hydroxybenzamide [60–61] unit have been targeted for analogue design in the quest for a more selective drug candidate over the last two decades. Although remarkable achievements have been made, the development of a unified synthetic strategy that would allow access to the natural product itself as well as some of its analogues remains important. A similar target is the closely related natural product ophiocordin (**2**). Herein, we describe a general approach to some of these targets.

## Results and Discussion

**Figure 2:**
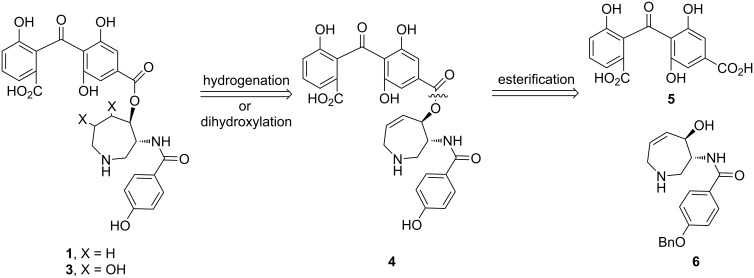
Strategic bond disconnections of balanol.

The key feature of our retrosynthetic analysis (Figure 2) is the identification of the dehydro derivative of balanol **4** as the unified precursor of balanol (**1**) and an azepin ring-modified balanol **3**. Derivative **4** could be obtained through esterification between the carboxylic acid **5** and the allylic alcohol **6**.

**Scheme 1:**
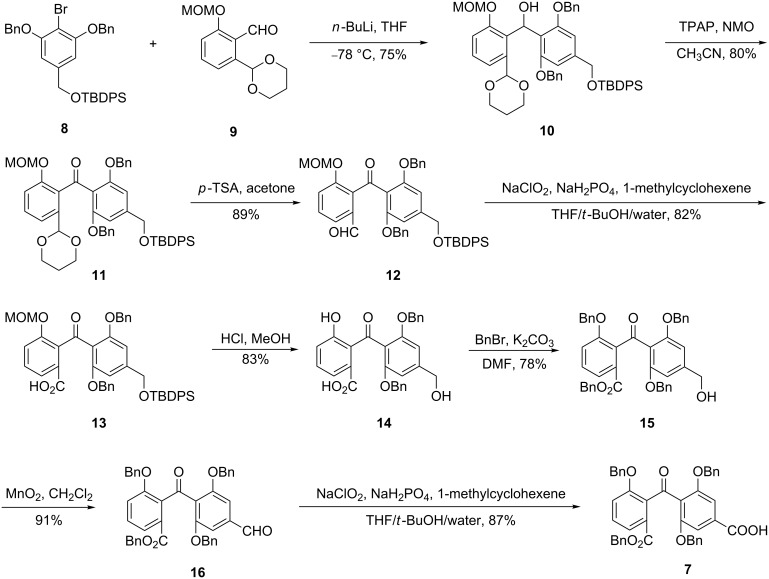
Synthesis of the benzophenone fragment of balanol.

We thus focused on the synthesis of the two key fragments **5** and **6**. The synthesis of the benzophenone unit has previously been achieved by several groups [27–30]. We adopted some of these methodologies with a number of modifications to prepare fragment **5** in its protected form **7** (Scheme 1). At first, the reaction of the known [17] bromo compound **8** with the known [27] aldehyde **9** in the presence of butyllithium effected a smooth conversion to the new benzylic alcohol **10**. The latter was oxidized with tetrapropylammonium perruthenate to provide the benzophenone derivative **11** in good yield. Subsequent cleavage of the 1,3-dioxane unit followed by oxidation of the resulting aldehyde **12** furnished carboxylic acid **13** in 73% overall yield over two steps. Concomitant removal of the phenolic MOM ether and the alcoholic TBDPS ether protecting groups in **13** under acidic conditions proceeded without significant loss of product to provide the dihydroxy acid **14** in good yield. Reaction of **14** with an excess of benzyl bromide in the presence of K_2_CO_3_ afforded simultaneous protection of the phenolic OH and the carboxylic acid functions leaving the primary alcohol function unprotected, as desired. Compound **15** was then converted following a literature procedure into the known [17] benzophenone **7** through two consecutive oxidations involving the aldehyde **16** as the intermediate. Taken as a hole the described synthesis of **7** from **8** and **9** proceeded in eight linear steps in an overall yield of 22%.

**Scheme 2:**
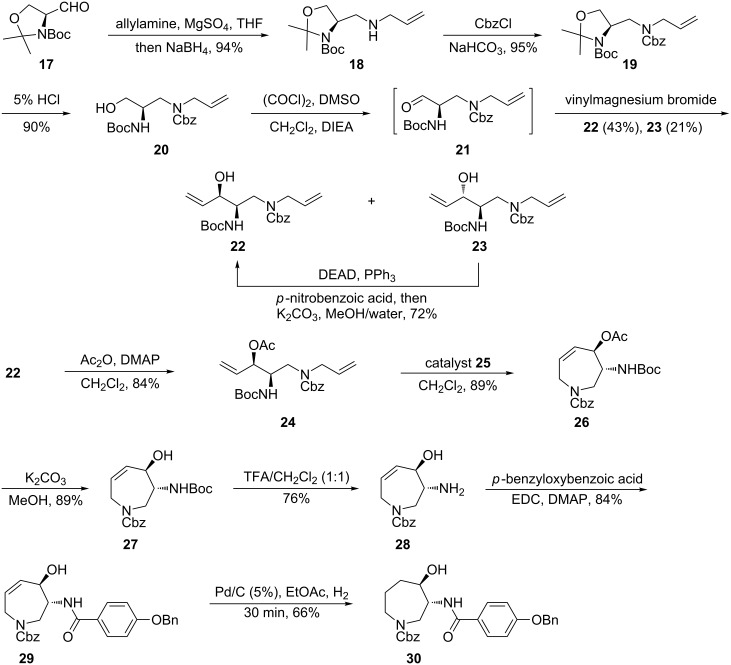
Synthesis of the hexahydroazepine core of balanol.

The synthesis of the azepine unit [31–47] was achieved following our preliminary report [62]. Thus, reductive amination of Garner’s aldehyde **17** (Scheme 2) with allylamine produced amine **18** which was N-protected with CbzCl to obtain **19** in an overall yield of 89% over three steps. The oxazolidine ring in compound **19** was then cleaved under acidic conditions and the resulting primary alcohol **20** was oxidized carefully under modified Swern conditions [63] to provide the α-chiral aldehyde **21** which was used directly in the next step. Addition of vinylmagnesium bromide to aldehyde **21** under optimized conditions gave a separable mixture of the allylic alcohols **22** and **23** in a combined yield of 64% over two steps. The undesired *anti*-isomer **23** could be effectively converted to the desired *syn*-isomer **22** by a Mitsunobu-type inversion [64].

The major *syn*-isomer **22** was then acetylated and the resulting diene **24** was subjected to ring-closing metathesis [65] in the presence of Grubbs’ second generation catalyst, benzylidene[1,3-bis(2,4,6-trimethylphenyl)-2-imidazolidinylidene]dichloro-(tricyclohexylphosphine)ruthenium (**25**). Pleasingly, the desired cycloalkene **26** was obtained in a gratifying yield of 89%. The sequential removal of the *O*-acetyl group leading to **27** followed by removal of the *N*-Boc group in the latter was executed under standard conditions to provide amine **28**. This was then coupled with 4-benzyloxybenzoic acid using EDC as activating agent to obtain the corresponding amide derivative **29** in an overall yield of 20% over eleven steps from **17**. The stereochemical identity of this tetrahydroazepine derivative was confirmed by its selective conversion to the corresponding known azepane derivative **30** which displayed optical and ^13^C NMR data nearly overlapping with those reported by Nicolaou et al [17].

**Scheme 3:**
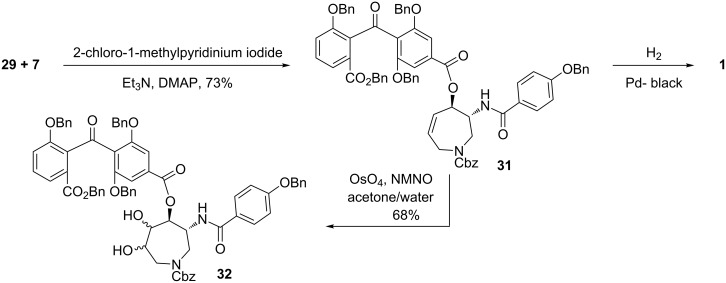
Synthesis of balanol and an analogue.

With the two key fragments **29** and **7** in hand, we next focused on their convergent combination. The esterification of the allylic alcohol **29** with the acid **7** (Scheme 3) proceeded best in the presence of Mukaiyama’s reagent [66], 2-chloro-1-methylpyridinium iodide, to provide the ester **31** in 73% yield. Simultaneous hydrogenolytic removal of the *O*-benzyl groups and the *N*-Cbz group under reported conditions finally provided the natural (−)-balanol in a yield of 41%. The product thus obtained displayed spectroscopic and optical data in close agreement to those reported for natural balanol [17].

We next focused our attention to demonstrate the utility of the intermediate coupled product **31** in a possible synthesis of an azepane ring-modified balanol derivative along the projected pathway. To this end, dihydroxylation of the adduct **31** was next attempted. Pleasingly, the dihydroxylation of **31** proceeded smoothly; however, unfortunately to provide an inseparable mixture of the two possible dihydroxylated isomers **32** in a combined yield of 68%. The isomeric composition of **32** was determined to be 81:19 by HPLC.

## Conclusion

In conclusion, we have developed a concise synthetic approach to the naturally occurring (−)-balanol (**1**) from easily available starting materials and reagents. Most of the synthetic steps proceeded in good to very good overall yield and stereocontrol. The developed synthesis may therefore be a complement to the existing literature. An attempted synthesis of an azepane ring-modified balanol derivative from a common precursor unfortunately was unsuccessful due to difficulty in separating stereoisomeric products. However, the intermediate **31** may prove to be useful in the synthesis of other analogues.

## Supporting Information

File 1 Experimental details and characterization data for the prepared compounds, copies of ^1^H and ^13^C NMR spectra of all new compounds, and data for the comparison of **30** and **1** with reported data.
