# Supplementary material for: A unified approach to the important protein kinase inhibitor balanol and a proposed analogue
Source: Beilstein J Org Chem. 2013 Dec 19;9:2910–5. doi: 10.3762/bjoc.9.327 (PMC3896276; doi:10.3762/bjoc.9.327)
Supplement: File 1 — Experimental details and characterization data for the prepared compounds, copies of 1H and 13C NMR spectra of all new compounds, and data for the comparison of 30 and 1 with reported data. [file Beilstein_J_Org_Chem-09-2910-s001.pdf]

Supporting Information

for

A unified approach to the important protein kinase inhibitor balanol and a proposed analogue

Tapan Saha, Ratnava Maitra, Shital K. Chattopadhyay*[§]

Address: Department of Chemistry, University of Kalyani, Kalyani - 741235, West Bengal, India

Email: Shital K. Chattopadhyay - skchatto@yahoo.com

[§]Corresponding author* Fax: +91+33+25828282

*Corresponding author

Experimental details and characterization data for the prepared compounds are included. Copies of ^{1}H and ^{13}C NMR spectra of all new compounds are also provided. Data for comparison of 30 and 1 with reported data are also presented

Table of contents

1. Experimental procedures S2

2. ^{1}H- and ^{13}C-NMR spectra of all new compounds S15

3. Comparison of data S39

4. References...S40

Experimental

General

Optical rotations were recorded in spectroscopic grade chloroform on a Jasco DIP-370 polarimeter, $[\alpha]_D$ values are recorded in units of 10^{-1} deg cm^2 g^{-1}. Infrared spectra were recorded on a Perkin-Elmer Spectrum-1 spectrophotometer purchased through a DST-FIST grant. Proton and carbon NMR spectra were recorded on a Bruker DRX-300 spectrometer. Chemical shifts are recorded relative to residual solvent or TMS as standard. Mass spectra were recorded on a JEOL-JMS 600 instrument from I. I. C. B., Kolkata or IACS, Kolkata. Petroleum ether refers to the fraction boiling in the range 60–80 °C. Silica gel (60–120 mesh) for column chromatography was purchased from Spectrochem, India.

2-(1,3-Dioxan-2-yl)-6-(methoxymethoxy)benzaldehyde (9):

n-BuLi (5.5 mL, 2 M in hexane) was added dropwise to a stirred solution of 2-(3-(methoxymethoxy)phenyl)-1,3-dioxane [1] (2.24 g, 10 mmol) in cyclohexane (15 mL) at room temperature and stirring was continued for 15 min. Then DMF (2.25 mL, 30 mmol) was slowly added to this reaction mixture and stirred for another 30 min. It was then quenched by slow addition of brine (20 mL) and extracted with ethyl acetate (2 × 50 mL). The combined organic extract was washed successively with water (2 × 50 mL) and brine (50 mL), and then dried (Na_2SO_4). It was filtered and the filtrate was concentrated in vacuo to leave a crude mass which on chromatographic purification over silica gel using a mixture of petroleum ether/ethyl acetate (95:5) as eluent provided compound **9** as a pale yellow solid (1.63 g, 65%). mp 58-60 °C. IR (KBr): 2930, 1694, 1598, 1590 cm^{-1}. ^{1}H NMR (400 MHz, $CDCl_3$): δ 10.63 (s, 1H), 7.54-7.50 (m, 2H), 7.27-7.21 (m, 1H), 6.26 (s, 1H), 5.27 (s, 2H), 4.23 (dd, *J* = 1.6, 5.2 Hz, 2H), 4.06 (dt, *J* = 12, 1.6 Hz, 2H), 3.49 (s, 3H), 2.25-2.20 (m, 1H), 1.47-1.43 (m, 1H). ^{13}C NMR (100 MHz, $CDCl_3$): δ 192.0, 159.8, 139.9, 134.7, 122.9, 119.9, 115.6, 97.8, 94.7, 67.6, 56.4, 25.8; HRMS (TOF MS ES+): *m/z* calcd for $C_{13}H_{16}NaO_5$ (M^++Na): 275.0895. Found: 275.0893.

(2-(1,3-Dioxan-2-yl)-6-(methoxymethoxy)phenyl)(2,6-bis(benzyloxy)-4-((*tert*-butyldiphenylsilyloxy)methyl)phenyl)methanol (10):

n-BuLi (0.7 mL, 2 M in hexane) was added dropwise to a stirred solution of **8** (0.955 g, 1.5 mmol) in THF (10 mL) at −78 °C, and stirring was continued for 5 min. A solution of the aldehyde **9** (250 mg, 1 mmol) in THF (5 mL) was then added dropwise to the reaction mixture at −78 °C. It was allowed to

come to room temperature over 2 h and then cooled to 0 °C before being quenched with saturated aqueous NH_4Cl (5 mL). It was extracted with ethyl acetate (2 × 50 mL) and the combined organic extract was washed successively with water (50 mL) and brine (50 mL). It was then dried (Na_2SO_4), filtered and the filtrate was concentrated in vacuo to leave a crude mass which was purified by chromatography over silica gel using a mixture of petroleum ether/ethyl acetate (85:15) as eluent to provide compound **10** as a colourless viscous liquid (0.6 g, 75%). IR (KBr): 3469, 2928, cm^{-1}. ^{1}H NMR (400 MHz, $CDCl_3$): δ 7.62 (d, *J* = 1.2 Hz, 4H), 7.43-7.41 (m, 3H), 7.4-7.34 (m, 4H), 7.32-7.23 (m, 6H), 7.21-7.7.12 (m, 4H), 6.98 (d, *J* = 12 Hz, 1H), 6.70 (d, *J* = 7.6 Hz, 1H), 6.56 (s, 2H), 6.11 (s, 1H), 5.23 (d, *J* = 8 Hz, 1H), 4.99 (s, 4H), 4.8 (dd, *J* = 10.8, 6.8 Hz, 2H), 4.63 (s, 2H), 4.13-4.08 (m, 2H), 3.98-3.82 (m, 1H), 3.69 (t, *J* = 12 Hz, 1H), 2.92 (s, 3H), 1.32-1.28 (m, 1H), 2.18-2.09 (m, 1H), 1.04 (s, 9H). ^{13}C NMR (100 MHz, $CDCl_3$): δ 157.1, 154.7, 141.4, 138.2, 136.9, 135.5, 133.4, 130.5, 129.7, 128.5, 127.7, 127.5, 127.2, 119.5, 118.6, 114.6, 103.7, 99.1, 93.7, 70.5, 67.3, 67.2, 66.7, 65.3, 55.4, 26.8, 25.7, 19.3. MS (TOF MS ES+): *m/z* (%) = 834 (M^{+}+Na, 100).

(2-(1,3-Dioxan-2-yl)-6-(methoxymethoxy)phenyl)(2,6-bis(benzyloxy)-4-((*tert*-butyldiphenylsilyloxy)methyl)phenyl)methanone (11):

A solution of the benzylic alcohol **10** (0.81 g, 1 mmol) in CH_3CN (15 mL) was stirred for 15 min in the presence of molecular sieves (4 Å, 2 g). 4-Methylmorpholine *N*-oxide (0.175 g, 1.5 mmol) and tetrapropylammonium perruthenate (17.5 mg, 0.05 mmol) were then sequentially added and the resulting mixture was stirred for 18 h at room temperature. It was then diluted with ethyl acetate, filtered through Celite, concentrated in vacuo and the residual mass was purified by chromatography over silica gel using a mixture of petroleum ether/ethyl acetate (85:15) to provide compound **11** as a colourless viscous liquid (0.645 g, 80%). IR (KBr): 2930, 2865, 1674, 1606, 1589 cm^{-1}. ^{1}H NMR (400 MHz, $CDCl_3$): δ 7.61 (d, *J* = 6.8 Hz, 4H), 7.43 (dt, *J* = 6.4, 0.8 Hz, 3H), 7.35 (m, 5H), 7.25-7.21 (m, 6H), 7.11-7.10 (m, 4H), 6.98 (d, *J* = 8 Hz, 1H), 6.55 (s, 2H), 5.61 (s, 1H), 4.96 (s, 4H), 4.73 (s, 2H), 4.66 (s, 2H), 4.08 (dd, *J* = 11.2, 4.4 Hz, 2H), 3.74 (t, *J* = 12 Hz, 2H), 2.95 (s, 3H), 2.1-2.14 (m, 1H), 1.31-1.24 (m, 1H), 1.06 (s, 9H). ^{13}C NMR (100 MHz, $CDCl_3$): δ 194.7, 157.8, 154.2, 145.5, 138.2, 136.7, 135.5, 133.2, 132.1, 130.2, 129.8, 128.4, 127.8, 127.5, 126.9, 119.9, 119.8, 115.2, 102.8, 99.5, 94.1, 70.0, 67.3, 65.3, 55.5, 26.8, 25.7, 19.3. HRMS (TOF MS ES+): *m/z* calcd for $C_{50}H_{53}O_8Si$(M^{+}+H): 809.3501. Found: 809.3505 (M^{+}+H).

2-(2,6-Bis(benzyloxy)-4-((*tert*-butyldiphenylsilyloxy)methyl)benzoyl)-3-(methoxymethoxy)benzaldehyde (12):

p-Toluenesulfonic acid (25 mg) was added to a solution of the benzophenone **11** (0.81 g, 1 mmol) in acetone/water (20 mL, 9:1) and the resulting solution was refluxed gently for 4 h. It was allowed to come to room temperature, concentrated in vacuo and then diluted with ethyl acetate (200 mL). The ethyl acetate extract was washed successively with water (100 mL) and brine (100 mL), and then dried (Na_2SO_4). It was filtered and the filtrate was concentrated in vacuo to leave a crude mass which was purified by chromatography over silica gel using a mixture of petroleum ether/ethyl acetate (85:15) as eluent to provide compound **12** as a colourless viscous liquid (0.67 g, 89%). IR (KBr): 2932, 2859, 1693, 1672, 1584 cm^{-1}. ^1H NMR (400 MHz, $CDCl_3$): δ 9.95 (s, 1H), 7.63 (d, *J* = 6.8 Hz, 4H), 7.46-7.43 (m, 3H), 7.38-7.35 (m, 5H), 7.25-7.19 (m, 7H), 7.11-7.08 (m, 4H), 6.61 (s, 2H), 4.93 (s, 4H), 4.81 (s, 2H), 4.70 (s, 2H), 3.00 (s, 3H), 1.08 (s, 9H). ^{13}C NMR (100 MHz, $CDCl_3$): δ 194.0, 191.3, 157.7, 154.6, 146.2, 136.5, 136.2, 136.1, 135.5, 133.2, 130.5, 129.9, 128.5, 127.9, 127.8, 127.2, 120.9, 119.5, 119.2, 102.7, 94.2, 70.4, 65.3, 55.8, 26.9, 19.3. MS (TOF MS ES+): *m/z* (%) = 774 (M^++Na, 100).

General procedure of the $NaClO_2$-oxidation of an aldehyde to a carboxylic acid:

Aq. NaH_2PO_4 (1.5 mL, 1 M), 1-methylcyclohexene (120 µL, 1 mmol) and $NaClO_2$ (181 mg, 2 mmol) were sequentially added to a solution of the aldehyde (0.5 mmol) in THF-*t*-BuOH-water (5 mL, 4:4:1), and the resulting mixture was stirred for 6–8 h at room temperature before being treated with aqueous $KHSO_4$ (5 mL, 0.5 M). The resulting reaction mixture was concentrated in vacuo and then extracted with ethyl acetate (2 × 50 mL). The combined organic extract was washed successively with water (50 mL), saturated aqueous sodium sulfite (10 mL) and brine (50 mL). It was then dried (Na_2SO_4), filtered and the filtrate was concentrated in vacuo to leave a crude mass which was purified by chromatography over silica gel to produce the corresponding pure carboxylic acid.

2-(2,6-Bis(benzyloxy)-4-((*tert*-butyldiphenylsilyloxy)methyl)benzoyl)-3-(methoxymethoxy)benzoic acid (13):

Yield: 82%. IR (KBr): 3403, 1767, 1696 cm^{-1}. ^1H NMR (400 MHz, $CDCl_3$) : δ 8.43 (s), 7.63 (dd, *J* = 7.6, 1.2 Hz), 7.46-7.35 (m), 7.22-7.21 (m), 7.08-7.06 (m), 6.80-6.78 (m), 6.55 (brs), 5.23 (brs), 5.16 (d, *J* = 6.4 Hz), 5.50 (d, *J* = 6.4 Hz), 4.92 (br s), 4.71-4.69 (overlapping singlets), 3.17(s), 1.07 (s). ^{13}C NMR (100 MHz, $CDCl_3$): δ 191.8, 170.7, 169.1, 159.1, 156.9, 154.1, 151.1, 146.6, 144.2, 139.7, 136.4, 135.5,

135.2, 133.2, 130.7, 129.9, 129.1, 129.0, 129.9, 129.1, 129.0, 128.7, 128.5, 128.3, 127.8, 127.6, 127.3, 123.7, 119.4, 118.9, 117.8, 112.0, 105.6, 104.5, 104.1, 102.9, 94.5, 93.8, 72.7, 70.7, 70.5, 65.3, 65.0, 55.9, 55.8, 29.7, 26.8, 19.3. HRMS (TOF MS ES+): *m/z* calcd for $C_{47}H_{46}NaO_8Si$ (M^+ + Na) : 789.2860. Found: 789.2858. [spectral data revealed the presence of an equilibrium between ketocarboxilic acid and its hemiketal form and hence counting of protons is not included. This has also been observed [2] by Nicolaou et al.].

3,5-Bis(benzyloxy)-4-(2-(benzyloxy)-6-benzyloxycarbonyl)benzoic acid (7):

Yield: 87%. IR (KBr): 3441, 1718, 1698, 1652, 1583 cm^{-1}. mp 158-160 °C [Lit. [2] mp 158-160 °C]; ^{1}H NMR (400 MHz, $CDCl_3$): δ 7.33-7.0 (m, 16H), 7.14 (t, *J* = 7.2 Hz, 2H), 7.08-7.07 (m, 4H), 6.97 (d, *J* = 8.4 Hz, 1H), 6.85 (d, *J* = 7.6 Hz, 2H), 5.14 (s, 2H), 4.80 (s, 4H), 4.72 (s, 2H). ^{13}C NMR (100 MHz, $CDCl_3$): δ 191.7, 171.0, 167.6, 157.9, 156.5, 136.1, 135.8, 135.6, 133.1, 131.7, 131.6, 130.9, 128.4, 128.3, 128.0, 127.7, 127.2, 124.7, 122.1, 115.2, 107.2, 70.6, 70.4, 67.2.

2-(2,6-Bis(benzyloxy)-4-(hydroxymethyl)benzoyl)-3-hydroxybenzoic acid (14):

Hydrochloric acid (12 N, 0.2 mL) was added to a solution of the carboxylic acid **13** (380 mg, 0.5 mmol) in methanol (6 mL) and the resulting mixture was stirred at 50 °C for 2 h. It was then concentrated in vacuo, diluted with water (25 mL) and extracted with ethyl acetate (2 × 25 mL). The combined organic extract was successively washed with saturated sodium bicarbonate (2 × 25 mL), water (25 mL) and brine (25 mL). It was then dried (Na_2SO_4), filtered and the filtrate was concentrated in vacuo to leave a crude solid which was purified over silica gel using a mixture of petroleum ether/ethyl acetate/methanol (50:48:2) to provide compound **14** as a colourless solid (200 mg, 83%). mp 210-212 °C. IR (KBr): 3488, 2865, 1692, 1625, 1610, 1576 cm_{-1}. ^{1}H NMR (400 MHz, DMSO-d_6): δ 10.04 (s, 1H), 7.28-7.24 (m, 7H), 7.20-7.18 (m, 4H), 7.02 (d, *J* = 7.6 Hz, 1H), 6.89 (dd, *J* = 8, 0.4 Hz, 1H), 6.72 (s, 2H), 5.36 (brs, 1H), 4.98 (s, 4H), 4.48 (s, 2H). ^{13}C NMR (100 MHz, DMSO-d_6): δ 193.1, 169.4, 158.3, 156.7, 147.8, 137.4, 134.8, 131.3, 128.6, 128.5, 127.8, 127.3, 120.0, 119.4, 118.5, 103.8, 70.1, 63.2. Anal. Calcd for $C_{29}H_{24}O_7$: C, 71.89; H, 4.99. Found: C, 71.62; H, 5.27.

Benzyl 3-(benzyloxy)-2-(2,6-bis(benzyloxy)-4 (hydroxymethyl)benzoyl) benzoate (15):

Potassium carbonate (175 mg, 1.25 mmol) was added to a stirred solution of **14** (0.242 g, 0.5 mmol) and benzyl bromide (125 µL, 1.05 mmol) in *N*,*N*-dimethylformamide (10 mL) and the resulting solution was stirred for 24 h at room temperature. The reaction mixture was then poured into water (50 mL) and extracted with ethyl acetate (2 × 25 mL). The combined organic extract was successively washed with water (2 × 25 mL) and brine (25 mL). It was then dried (Na_2SO_4), filtered and concentrated in vacuo to leave a crude solid which was purified over silica gel using a mixture of petroleum ether/ethyl acetate (6:4) to provide the *O*-benzylated compound **15** as a colourless solid (0.26 g, 78%). mp 138-140 °C [Lit. [2] mp 138.5-139.5 °C]; IR (KBr): 3293, 1697, 1656, 1614, 1535 cm^{-1}. ^{1}H NMR (400 MHz, $CDCl_3$): δ 7.22-7.21 (m, 5H), 7.20-7.15 (m, 9H), 7.13-7.12 (m, 2H), 7.09-7.06 (m, 4H), 6.97 (d, *J* = 7.2 Hz, 2H), 6.89 (d, *J* = 8 Hz, 1H), 6.50 (s, 2H), 5.12 (s, 2H), 4.77 (s, 6H), 4.60 (d, *J* = 3.2 Hz, 2H) 1.89 (brs, 1H). ^{13}C NMR (100 MHz, $CDCl_3$): δ 192.4, 167.0, 159.0, 155.8, 147.0, 136.6, 136.3, 136.0, 134.4, 131.5, 129.6, 128.3, 128.2, 128.1, 127.9, 127.6, 127.5, 127.2, 127.1, 122.2, 118.3, 115.6, 103.4, 70.5, 70.4, 66.8, 64.6. HRMS (TOF MS ES+): *m/z* calcd for $C_{43}H_{36}NaO_7$: 687.2359 (M^++Na): Found : 687.2357.

Benzyl 3-(benzyloxy)-2-(2,6-bis(benzyloxy)-4-formylbenzoyl)benzoate (16):

A solution of the alcohol **15** (332 mg, 0.5 mmol) in dichloromethane (10 mL) was stirred with MnO_2 (435 mg, 5 mmol) for 12 h. It was then filtered through Celite and the filtrate was concentrated in vacuo to leave a crude mass which was purified over silica gel using a mixture of petroleum ether/ethyl acetate (3:1) as eluent to provide the aldehyde **16** as a colourless solid (300 mg, 91%). mp 110-112 °C [Lit. [2] mp 110-112 °C]; IR (KBr): 1721, 1695, 1670, 1579 cm^{-1} . ^{1}H NMR (400 MHz, $CDCl_3$): δ 9.81 (s, 1H), 7.3 (t, *J* = 8 Hz, 1H), 7.26-7.21 (m, 13H), 7.11-7.06 (m, 6H), 6.97 (d, *J* = 8.4 Hz, 1H), 6.90 (s, 2H), 6.83 (d, *J* = 7.6 Hz, 2H), 5.14 (s, 2H), 5.81 (s, 4H), 4.70 (s, 2H); ^{13}C NMR (100 MHz, $CDCl_3$): δ 191.5, 191.4, 167.6, 158.3, 156.7, 138.2, 136.0, 135.8, 135.5, 133.4, 131.3, 131.1, 128.4, 128.3, 128.2, 128.0, 127.9, 127.8, 127.7, 127.1, 125.3, 122.0, 115.1, 106.5, 70.6, 70.4, 67.2. MS (TOF MS ES+): *m/z* (%) = 685 (M^++Na, 100).

(*R*)-*tert*-Butyl 4-((allylamino)methyl)-2,2-dimethyloxazolidine-3-carboxylate (18):

A solution of Garner's aldehyde **17** (1.15 g, 5 mmol) in tetrahydrofuran (15 mL) was stirred at room temperature with allyl amine (410 µL, 5.5 mmol) and $MgSO_4$ (2.65 g, 22 mmol) under nitrogen for

12 h. Then it was cooled to 0 °C and $NaBH_4$ (0.208 g, 5.5 mmol) was added in portions under nitrogen atmosphere. The reaction mixture was allowed to come to room temperature and stirred for 12 h. It was then quenched by adding of 5% NH_4Cl solution at 0 °C. It was diluted with water (50 mL) and extracted with ethyl acetate (2 x 50 mL). The combined organic layer was washed successively with water (2 × 50 mL) and brine (50 mL). It was then dried (Na_2SO_4), filtered and the filtrate was concentrated in vacuo to leave a pale yellow liquid which was purified by chromatography over silica gel using a mixture of petroleum ether/ethyl acetate (93:7) as eluent to give the product **18** as a colourless liquid (1.28 g, 94%). $[\alpha]_D^{25} = +15.8$ (*c* 1.56, $CHCl_3$). IR (KBr): 3438, 1700 cm^{-1}. ^1H NMR (400MHz, $CDCl_3$) : δ 5.77-5.69 (m, 1H), 5.02 (d, *J* =17.2 Hz, 1H), 4.94 (br m, 1H), 3.79 (s, 2H), 3.68 (br s, 1H), 3.12-3.11 (m, 2H), 2.76-2.65 (m, 1H), 2.50-2.48 (m, 1H), 1.48-1.33 (overlapping singlets, 15H). ^{13}C NMR (100MHz, $CDCl_3$): δ 152.4 (151.7), 136.8, 115.8, 93.3 (93.7), 80.1 (79.6), 66.3, 57.4 (57.1), 52.3 (52.2), 51.0 (50.9), 28.4, 27.5 (26.7), 24.3 (23.0).

(*R*)-*tert*-Butyl 4-((allyl(benzyloxycarbonyl)amino)methyl)-2,2-dimethyloxazolidine-3-carboxylate (19):

Water (0.5 mL) and benzyl chloroformate (0.78 mL, 5.5 mmol) were added sequentially at room temperature to a stirred solution of the amine **18** (1.35 g, 5 mmol) and sodium bicarbonate (1.26 g, 15 mmol) in ethyl acetate (20 mL), and the resulting reaction mixture was stirred overnight. It was quenched by dropwise addition of pyridine (350 µL, 4.3 mmol) and then stirred for 10 min before being diluted with ethyl acetate (50 mL). The combined organic layer was washed successively with 1 N HCl (50 mL), water (50 mL), brine (50 mL) and then dried over Na_2SO_4. It was filtered and the filtrate was concentrated under reduced pressure to leave a pale yellow liquid which was purified by chromatography over silica gel with petroleum ether/ethyl acetate (93:7) as eluent to give the product **19** as a colourless liquid (1.8 g, 95 %). $[\alpha]_D^{25} = +5$ (*c* 1.75, $CHCl_3$). IR(neat): 1697, 1645, 1398, 1233, 1075,769 cm^{-1}. ^1H NMR (400MHz, $CDCl_3$): δ 7.38-7.35 (m,5H), 5.79 (br s, 1H), 5.16 (m, 4H), 4.11-4.01 (m, 5H), 3.95-3.87 (m, 1H), 3.65-3.26 (m, 1H), 1.6-1.48 (overlapping siglets,15H). ^{13}C NMR (75 MHz, $CDCl_3$): δ 155.9 (156.5), 151.5 (152.1), 136.4, 133.0, 127.7 (128.3), 127.2, 126.7, 116.5 (117.0), 93.3 (94.0), 79.8 (80.0), 67.1, 65.1 (65.7), 55.7 (55.9), 49.8 (50.0), 47.4 (48.0), 28.2, 26.8 (27.4), 22.9 (24.2). HRMS (TOF MS ES+): *m/z* calcd for $C_{22}H_{32}N_2NaO_5$ 427.2209 (M^++Na): Found: 427.2208.

(*R*)-benzyl allyl(2-((*tert*-butoxycarbonyl)amino)-3-hydroxypropyl)carbamate (20)

Hydrochloric acid (1.25 mL, 5%) was added dropwise while stirring to an ice-cooled solution of compound **19** (0.5 g, 1.24 mmol) in methanol (10 mL) and stirring was continued for 1 h at 0 °C, and then at room temperature for 5 h. The reaction mixture was quenched with saturated sodium bicarbonate solution and then extracted with ethyl acetate (2 x 25 mL). The combined organic extract was washed successively with water (10 mL), brine (10 mL) and then dried (Na_2SO_4). It was filtered and the filtrate was concentrated under reduced pressure to leave a crude product which on chromatography over silica gel using a mixture of petroleum ether/ethyl acetate (4:1) afforded the product **20** as a colourless viscous liquid (405 mg, 90%). $[\alpha]_D^{25} =$ - 8 (*c* 3.00, $CHCl_3$). IR (neat): 3435, 1702, 1693, 1391, 1248, 1169, 1063 cm^{-1}. ^1H NMR (400 MHz, $CDCl_3$): δ 7.37-7.25 (m, 5H), 5.79-5.72 (m, 1H), 5.17-5.14 (m, 4H), 3.97 (dd, *J* = 14, 5 Hz, 1H), 3.80 (dd, *J* = 16, 5.5 Hz, 1H), 3.68-3.62 (m, 1H), 3.59-3.56 (m, 3H), 3.50-3.47 (m, 1H), 3.13(dd, *J* = 14, 5 Hz, 1H), 1.68 (br s, 1H), 1.42 (s, 9H). ^{13}C NMR (75 MHz, $CDCl_3$): δ 157.6, 155.6, 136.1, 132.8, 128.4, 128.0, 127.6, 117.2, 79.3, 67.6, 64.8, 61.4, 50.1, 46.6, 28.2; MS (TOF MS ES+): *m/z* (%) = 387 (M^+ + Na, 100).

***tert*-Butyl (2*R*,3*R*)-1-(*N*-benzyloxycarbonyl(allyl)amino)-3-hydroxypent-4-en-2-ylcarbamate (22) and *tert*-butyl (2*R*,3*S*)-1-(*N*-benzyloxycarbonyl(allyl)amino)-3-hydroxypent-4-en-2-ylcarbamate (23):**

A solution of DMSO (310 µL, 4.2 mmol) in CH_2Cl_2 (2 mL) was added dropwise to a stirred solution of oxalyl chloride (155 µL, 1.8 mmol) in CH_2Cl_2 (3 mL) at −78 °C. It was then stirred for 20 min at the same temperature. A solution of the alcohol **20** (0.55 g, 1.5 mmol) in CH_2Cl_2 (5 mL) was then added dropwise to the reaction mixture. It was then warmed to −25 °C and stirred at this temperature for 35 min followed by dropwise addition of diiospropylethylamine (1.8 mL, 10.5 mmol). Stirring was continued at the same temperature for another 10 min. The reaction mixture was cooled back to −78 °C and diluted with THF (20 mL). Then vinylmagnesium bromide (6 mL, 1 M solution in THF) was added dropwise to the reaction mixture. The reaction mixture was allowed to warm to −40 °C and stirred at this temperature for 2 h. Then the reaction mixture was allowed to come to room temperature and stirred for 8 h before being quenched by slow addition of saturated aq NH_4Cl (10 mL) at 0 °C. It was extracted with ethyl acetate (2 × 25 mL) and the combined organic extract was washed successively with HCl (1 N, 2 × 20 mL), H_2O (2 × 20ml) and brine (20 mL). After drying (Na_2SO_4), it was filtered and the filtrate was concentrated in vacuo to leave a pale yellow crude product which on chromatographic purification over silica gel using a mixture of petroleum

ether/ethyl acetate (85:15) as eluent provided sequentially the diastereomeric alcohols **22** and **23** as colorless liquids in a combined yield of 64%.

Compound 22: Yield: 0.255 mg (43.5%). $[\alpha]_D^{25}$ = - 8 (*c* 1.5, $CHCl_3$). IR (neat): 3416, 1687 cm^{-1}. ^1H NMR (400MHz, $CDCl_3$) : δ 7.36-7.34 (m, 5H), 5.85-5.74 (m, 2H), 5.37 (d, *J* = 16 Hz, 1H), 5.21-5.15 (m, 5H), 4.94 (d, *J* = 8 Hz, 1H), 4.16-3.98 (br m, 3H), 3.89-3.62 (br m, 3H), 3.14(dd, *J* = 13.6, 4.8 Hz, 1H), 1.45-1.41 (s, 9H). ^{13}C NMR (100MHz, $CDCl_3$): δ 157.9, 156.0, 136.7, 136.2, 132.8, 128.6, 128.2, 127.8, 117.6, 116.0, 79.4, 69.4, 67.9, 52.5, 50.3, 47.7, 28.3 (27.9). HRMS (TOF MS ES+) *m/z* calcd for $C_{21}H_{30}N_2NaO_5$ (M^++Na): 413.2052. Found. 413.2051.

Compound 23: Yield: 0.12 g (20.5%). $[\alpha]_D^{25}$ = + 9 (*c* 2.0, $CHCl_3$). IR (KBr): 3437, 2977, 2930, 1698 cm^{-1}. ^1H NMR (400 MHz, $CDCl_3$): δ 7.37-7.33 (m, 5H), 5.88-5.73 (m, 2H), 5.30 (d, *J* = 17 Hz, 1H), 5.16-5.10 (m, 7H), 4.14 (br s, 1H), 3.94-3.78 (m, 3H), 3.56-3.52 (m, 1H), 3.33-3.30 (m, 1H), 1.43 (s, 9H). ^{13}C NMR (100 MHz, $CDCl_3$): δ 157.5, 156.3, 137.0, 136.3, 133.0, 128.5, 128.1, 127.8, 117.2, 115.7, 79.7, 73.8, 67.6, 54.0, 50.0, 46.0, 28.3. HRMS (TOF MS ES+) *m/z* calcd for $C_{21}H_{30}N_2NaO_5$ (M^++Na): 413.2052. Found. 413.2054.

Mitsunobu inversion of 23 to 22:

4-Nitrobenzoic acid (167 mg, 1 mol), PPh_3 (262 mg, 1 mmol) and diethylazodicarboxylate (157.5 µL, 1 mmol) were sequentially added to a solution of **23** (195 mg, 0.5 mmol) in anhydrous THF (10 mL) at 0 °C. Then the reaction mixture was allowed to come to room temperature and stirred for 24 h. Then it was diluted with ethyl acetate (25 mL) and washed successively with saturated aqueous $NaHCO_3$ and then with water (20 mL) and brine (20 mL). It was then dried (Na_2SO_4), filtered and the filtrate was concentrated in vacuo to leave a crude product which was used without further purification for the next step. A solution of this crude product in methanol/water (10 mL, 9:1) was stirred with K_2CO_3 (350 mg, excess) for 4 h. Then the reaction mixture was filtered and concentrated in vacuo, diluted with ethyl acetate (25 mL), washed with water (20 mL) and brine (20 mL), and then dried (Na_2SO_4), filtered and concentrated in vacuo to provide a crude liquid which was purified by column chromatography over silica gel using a mixture of petroleum ether/ethyl acetate (85:15) to produce compound **22** as colourless liquid (140 mg, 72%).

(3*R*,4*R*)-5-(*N*-Benzyloxycarbonyl(allyl)amino)-4-(*tert*-butoxycarbonylamino)pent-1-en-3-yl acetate (24):

Acetic anhydride (71 µL, 0.75 mmol) and DMAP (0.01 g) were added to a stirred solution of the alcohol **22** (195 mg, 0.5 mmol) in CH_2Cl_2 (5 mL) at room temperature under nitrogen and the reaction mixture was stirred for 12 h before being poured into ice-cooled water (20 mL). The resulting mixture was stirred for 30 min and then extracted with CH_2Cl_2 (2 × 25 mL). The combined organic extract was washed with H_2O (2 × 25 mL) followed by brine (25 mL). It was dried over Na_2SO_4, filtered and the filtrate was concentrated in vacuo to leave a crude product which was purified by column chromatography over silica gel using a mixture of petroleum ether/ethyl acetate (9:1) to produce compound **24** as a colourless liquid (182 mg, 84%). $[\alpha]_D^{25} =$ - 15 (*c* 2.0, $CHCl_3$). IR (neat): 3352, 1745, 1712, 1644, 1502, 1367, 1235, 1166, 1025, 770, 699 cm^{-1}. ^1H NMR (400 MHz, $CDCl_3$): δ 7.39-7.36 (m, 5H), 5.84-5.73 (m, 2H), 5.31-5.14 (m, 7H), 4.91 (d, *J* = 9.6 Hz, 1H), 4.17-4.06 (m, 2H), 3.82-3.78 (m, 1H), 3.57-3.51 (m, 1H), 3.38-3.24 (m, 1H), 2.13(1.89) (s, 3H), 1.46 (1.42) (s, 9H); ^{13}C NMR (100 MHz, $CDCl_3$): δ 170.5 (170.3), 157.3, 155.8 (155.5), 136.5(136.2), 133.2, 133.1(132.7), (128.6) 128.5, 128.3 (128.0), 127.7, 118.3 (118.1), 117.1 (117.6), 79.5 (79.9), 73.6 (73.5), 67.4 (67.7), 51.0(50.5), 49.3 (49.7), 45.4 (44.7), 35.4, 28.3, 21.0 (20.8). MS (TOF MS ES+): *m/z* (%) = 455 (M^++Na, 100). Anal. Calcd for $C_{23}H_{32}N_2O_6$: C, 63.87; H, 7.46; N, 6.48. Found: C, 64.09; H, 7.65; N, 6.61.

(3*R*,4*R*,*Z*)-Benzyl 4-acetoxy-3-(*tert*-butoxycarbonylamino)-2,3,4,7-tetrahydro-1*H*-azepine-1-carboxylate (26):

Catalyst **25** (0.011 g, 0.0125 mmol) was added to a stirred solution of the diene **24** (110 mg, 0.24 mmol) in dry and degassed CH_2Cl_2 (25 mL) under argon and the homogeneous mixture was gently refluxed for 12 h. It was then concentrated in vacuo to leave a crude product which was purified by chromatography over silica gel using a mixture of petroleum ether/ethyl acetate (85:15) to produce compound **26** as colourless viscous liquid (95 mg, 89 %); $[\alpha]_D^{25} =$ **-12** (*c* 2.0, $CHCl_3$). IR(KBr): 3350, 1738, 1713, 1699, 1504,1367, 1240, 1167, 1037, 771 cm^{-1}. ^1H NMR (400MHz, $CDCl_3$) : δ 7.39-7.33 (m,5H), 5.74-5.48 (m, 3H), 5.24-5.12 (m, 2H), 4.79 (d, *J* = 9.2, 1H), 4.59-4.46 (m, 1H), 4.25-4.11 (m, 1H), 3.89-3.65 (m, 2H), 3.51 (dd, *J* = 15, 7.2, 1H), 2.06-2.02 (m,3H), 1.43-1.38 (singlets, 9H). ^{13}C NMR (75 MHz, $CDCl_3$): δ 170.4 (170.3), 156.5 (155.5), 155.3 (154.9), 136.1, 129.4, 129.0, 128.5(128.4), 128.1, 127.7 (127.2), 79.2 (79.4), 72.9 (72.0), 67.6, 53.6 (52.8), 49.9 (49.0), 48.3 (47.9), 28.2, 20.7 (20.8). HRMS (TOF MS ES+): calcd for $C_{21}H_{28}N_2NaO_6$ (M^+ + Na): 427.1845. Found: 427.1848.

(3*R*,4*R*,*Z*)-Benzyl 3-(*tert*-butoxycarbonylamino)-4-hydroxy-2,3,4,7-tetrahydro-1*H*-azepine-1-carboxylate (27):

K_2CO_3 (345 mg, 2.5 mmol) was added to a stirred solution of **26** (100 mg, 0.25 mmol) in MeOH (2 mL) and the resulting mixture was stirred for 4 h at room temperature. Then the reaction mixture was filtered, concentrated in vacuo and extracted with ethyl acetate (2 × 25 mL). The ethyl acetate extract was washed with water (2 × 25 mL) and brine (25 mL), and dried (Na_2SO_4). It was filtered and the filtrate was concentrated in vacuo to leave a crude product which on chromatography over silica gel using a mixture of petroleum ether/ethyl acetate (5:1) provided the product **27** as a colourless viscous liquid (80 mg, 89%); $[\alpha]_D^{25} =$ - 20.0 (*c* 2.0, $CHCl_3$). IR (neat): 3410, 1694 cm^{-1}. ^1H NMR (400MHz, $CDCl_3$) : δ 7.37-7.35 (m, 5H), 6.35 (br s, 1H), 5.69 (d, *J* = 8 Hz, 1H), 5.41 (d, *J* = 11 Hz, 1H), 5.21-5.12 (m, 2H), 4.58-4.54 (m, 2H), 4.17-4.11 (m,1H), 3.92-3.91 (m, 1H), 3.82 (d, *J* = 15 Hz, 1H), 3.67-3.62 (m, 1H), 3.50 (dd, *J* = 15, 6 Hz, 1H), 1.45-1.40 (s, 9H); ^{13}C NMR (400MHz, $CDCl_3$) : δ 157.9, 157.0, 136.1, 134.2 (133.5), (128.8) 128.6, (128.5) 128.4, (128.3) 127.8, 124.4, 80.3, 73.8, (68.0) 67.8, 58.1 (56.7), 49.2, 48.7 (48.4), 28.4; HRMS (TOF MS ES+): *m/z* calcd for $C_{19}H_{26}N_2NaO_5$ (M^++Na): 385.1740. Found: 385.1755.

(3*R*,4*R*,*Z*)-Benzyl 3-amino-4-hydroxy-2,3,4,7-tetrahydro-1*H*-azepine-1-carboxylate (28):

An ice-cooled solution of TFA (1 mL, excess) in CH_2Cl_2 (1 mL) was added to the alcohol **27** (70 mg, 0.2 mmol) at 0 °C. The reaction mixture was allowed to come to room temperature and stirred for another 1 h. Then the reaction mixture was concentrated in vacuo, basified by slow addition of saturated aqueous sodium bicarbonate (10 mL), and extracted with CH_2Cl_2 (2 × 25 mL). The combined organic extract was washed successively with water (2 × 25 mL) and brine (25 mL), dried (Na_2SO_4), filtered and the filtrate was concentrated in vacuo to provide a crude mass. It was then purified by chromatography over silica gel using a mixture of chloroform/methanol (95:5) to provide the product **28** as colourless liquid (39 mg, 76 %). $[\alpha]_D^{25} =$ - 12.8 (*c* 1.3, $CHCl_3$). IR(KBr): 3357, 3290,1683 cm^{-1}. ^1H NMR (400MHz, $CDCl_3$) : δ 7.37-7.35 (m, 5H), 5.77-5.71 (m, 2H), 5.20-5.11 (m, 2H), 4.16-4.10 (m, 1H), 3.99 (t, *J* = 13 Hz, 1H), 3.85-3.80 (m, 2H), 3.47-3.37 (m, 1H), 2.89-2.85 (m, 1H), 2.55 (br s, 3H). ^{13}C NMR (400MHz, $CDCl_3$): δ 156.1 (155.6), 136.5, 135.2 (134.7), (128.6) 128.5, (128.2) 128.1, 127.7, (126.7) 126.3, 73.1 (72.6), (67.5) 67.4, 55.6 (55.3), 53.3 (52.9), (46.7) 46.5. HRMS (TOF MS ES+): *m/z* calcd for $C_{14}H_{19}N_2O_3$ (M^++H): 263.1396. Found: 263.1395.

(3*R*,4*R*,*Z*)-Benzyl 3-(4-(benzyloxy)benzamido)-4-hydroxy-2,3,4,7-tetrahydro-1*H*-azepine-1-carboxylate (29):

A suspension of the amino alcohol **28** (33 mg, 0.125 mmol), *p*-benzyloxybenzoic acid (28 mg, 0.125 mmol) and EDC·HCl (29 mg, 0.125 mmol) in CH_2Cl_2 (2.5 mL) was treated with DMAP (8 mg, 0.06 mmol) at room temperature under nitrogen and the resulting solution was stirred for 24 h. It was then quenched with water (5 mL) and extracted with CH_2Cl_2 (2 × 20 mL). The combined organic extract was washed successively with water (2 × 20 mL) and brine (20 mL), and dried (Na_2SO_4). It was filtered and the filtrate was concentrated in vacuo to provide a crude mass which was purified by chromatography over silica gel using a mixture of petroleum ether/ethyl acetate/methanol (60:40:1) as eluent to provide compound **29** as a gummy liquid (51 mg, 84%). $[\alpha]_D^{25} =$ - 45.2 (*c* 1.67, $CHCl_3$). IR (KBr): 3391, 1720, 1661 cm^{-1}. ^1H NMR (400MHz, $CDCl_3$) : δ 8.67 (d, *J* = 5.6 Hz, 1H), 7.84 (d, *J* = 8.8 Hz, 2H), 7.45-7.35 (m, 11H), 7.03 (d, *J* = 8.8 Hz, 2H), 5.74 (d, *J* = 12 Hz, 1H), 5.43-5.41 (m, 1H), 5.21 (s, 2H), 5.13 (s,2H), 4.66-4.61 (m,2H), 4.30 (dd, *J* = 12.0, 5.8 Hz, 1H), 4.0-3.92 (m, 1H), 3.71 (d, *J* = 18 Hz, 1H), 3.62 (dd, *J* = 15.2, 5.6 Hz, 1H). ^{13}C NMR (400MHz, $CDCl_3$): δ 168.8, 161.7, 157.7, 136.4, 136.0, 134.6, 129.2, 128.7, 128.6, 128.4, 128.2, 127.9, 127.5, 125.6, 123.9, 114.7, 74.6, 70.1, 68.1, 59.4, 48.8 48.5. MS (TOF MS ES+): *m/z* (%) = 495 (M^+ +Na, 100).

(3*R*,4*R*)-Benzyl 3-(4-(benzyloxy)benzamido)-4-hydroxyazepane-1-carboxylate (30):

A solution of the olefin **29** (25 mg, 0.022 mmol) in ethyl acetate (2 mL) was stirred vigorously in the presence of Pd/C (5%, 5 mg) at room temperature under hydrogen for 30 min. It was then filtered through Celite, washed with ethyl acetate (5 mL) and the combined filtrate was concentrated in vacuo to provide a crude product which on column chromatography over silica gel using a mixture of petroleum ether/ethyl acetate (60:40) as eluent produced compound **30** as a colourless solid (16 mg, 66%). mp 120-122 °C [Lit. [2] mp: 123 ^{0}C]. $[\alpha]_D$: - 72.0 (*c* 2.0, $CHCl_3$). [Lit. [2] $[\alpha]_D$: - 73.0 (*c* 1.0, $CHCl_3$).]. IR (KBr): 3442, 1502, 1367, 1235, 1166, 1025, 770,699 cm^{-1}. ^1H NMR (500 MHz, $CDCl_3$): δ 8.72 (d, *J* = 5.4 Hz, 1H), 7.81 (d, *J* = 8.8 Hz, 2H), 7.44-7.29 (m, 10H), 7.02 (d, *J* = 8.8 Hz, 2H), 5.43 (br s, 1H), 5.20 (ABq, *J* = 12.3 Hz, 2H), 5.13 (s, 2H), 4.20 (dd, J = 14.2, 3.75 Hz, 2H), 4.16 (d, J = 15.8 Hz, 1H), 4.10-4.06 (m, 1H), 3.76 (dd, J = 8.9, 6.5 Hz, 1H), 3.34 (dd, *J* = 5.1, 15.3 Hz, 1H), 2.79 (dt, J = 13.6, 3.4 Hz, 1H), 1.97-1.93 (m, 1H), 1.90-1.81 (m, 2H), 1.70-1.67 (m, 1H). ^{13}C NMR (75 MHz, $CDCl_3$): δ 168.5, 161.6, 157.7, 136.4, 136.2, 129.1, 128.6, 128.4, 128.2, 128.1, 127.7, 127.4, 125.7, 114.7, 79.5, 70.0, 67.8, 60.7, 50.4, 50.2, 32.7, 27.2.MS (TOF MS ES^+): *m/z* (%) = 513 (M^++K, 100).

(3*S*,4*S*,*Z*)-Benzyl 3-(4-(benzyloxy)benzamido)-4-(3,5-bis(benzyloxy)-4-(2-(benzyloxy)-6-(benzyloxy-carbonyl)benzoyl)benzoyloxy)-2,3,4,7-tetrahydro-1*H*-azepine-1-carboxylate (31):

A mixture of the amido alcohol **29** (25 mg, 0.05 mmol), acid **7** (35 mg, 0.05 mmol) and 2-chloro-1-methylpyridinium iodide (16.5 mg, 0.065 mmol) in CH_2Cl_2 (2.5 mL) was treated with Et_3N (15 µL, 0.1 mmol) at room temperature under nitrogen and stirred for 30 min. Then DMAP (3 mg, 0.025 mmol) was added and the resulting mixture was stirred for another 1 h at room temperature. It was then filtered through a pad of silica, concentrated in vacuo to produce a crude mass which on chromatographic purification over silica gel using a mixture of petroleum ether/ethyl acetate (7:3) as eluent provided the compound **31** (41 mg, 73%). $[\alpha]_D^{25}$ = - 66.1 (*c* 1.1, $CHCl_3$). IR(KBr): 3351,2924, 1728, 1660 cm^{-1}. ^{1}H NMR (400MHz, $CDCl_3$) : δ 7.86 (d, *J* = 8.8 Hz, 1H), 7.76 (d, *J* = 8.4 Hz, 2H), 7.45-7.35 (m, 12H), 7.30-7.28 (m, 4H), 7.25-7.24 (m, 3H), 7.21-7.71 (m, 14H), 7.11-7.10 (m, 6H), 7.06-7.02 (m, 2H), 6.92-6.89 (m, 2H), 6.83-6.82 (m, 2H), 5.80-5.73 (m, 2H), 5.67-5.64 (m, 1H), 5.36-5.33 (m, 1H), 5.26-5.23 (m, 1H), 5.10-5.07 (m, 3H), 5.04-5.03 (m, 3H), 4.9-4.79 (m, 6H), 4.68 (s, 2H), 4.07-4.03 (m, 1H), 3.77 (d, *J* = 16.4 Hz, 1H), 3.68 (dd, *J* = 15, 6.8 Hz, 1H). ^{13}C NMR (400MHz, $CDCl_3$) : δ 191.7, 167.33, (166.1) 165.9, 161.4, 158.1 (158.0), 157.4 (156.2), 136.4 (136.0), 135.8, 132.6 (132.4), 130.5 (130.4), 129.6, 128.9, 128.7 (128.6), 128.5, 128.9, 127.7 (128.6), 128.5, 128.4, 128.3, 128.2, 128.18, 128.0, 127.9, 127.7, 127.5, 127.4, 127.3, 127.0, 126.6, 126.1, 123.7, 122.0, 115.4, 114.7, 107.1, 75.1, 70.5, 70.0, 68.2, 67.0, 52.8, 50.0, 49.1. HRMS (TOF MS ES+): *m/z* calcd for $C_{71}H_{60}N_2O_{12}Na$ (M^++ Na): 1155.4044. Found: 1155.4070. [The compound exhibited extensive rotamerism and hence the number of proton and carbon signals exceeds. This has also been observed [3] by Nicolaou et al.]

Balanol (1):

To a solution of protected balanol **31** (20 mg, 0.018 mmol) in formic acid (5 mL) was added palladium black (50 mg) and the reaction mixture was allowed to stir vigorously for 18 h. The reaction mixture was then filtered through a pad of silica gel and the filtrate was concentrate under vacuo to provide a crude mass which on repeated purification by preparative TLC following Nicolaou's protocol provided (−)-balanol (**1**) as yellowish amorphous powder (4 mg, 41%). $[\alpha]_D^{25} = -106.0$ (*c* 0.10 MeOH) [Lit. [3] $[\alpha]_D^{25} = -$ 111.0 (*c* 0.10 MeOH)] [Lit. [4] $[\alpha]_D^{25} = -$ 107.0 (*c* 0.252 MeOH)] . ^{1}H NMR (500MHz, CD_3OD+ D_2O) : 7.58 (d, *J* = 8.4 Hz, 2H), 7.39 (d, *J* = 7.2 Hz, 1H), 7.18 (t, *J* = 9.0 Hz, 1H), 6.82 (s, 2H), 6.71 (d, *J* = 7.6 Hz, 2H), 5.39 (m, 1H), 4.47 (m, 1H), 3.52-2.92 (m, 4H), 2.19-2.02 (m, 4H).MS (TOF MS ES^+): *m/z* = 573.4 (M^++Na).

(3*R*,4*S*)-Benzyl 3-(4-(benzyloxy)benzamido)-4-(3,5-bis(benzyloxy)-4-(2-(benzyloxy)-6-(benzyloxy-carbonyl)benzoyl)benzoyloxy)-5,6-dihydroxyazepane-1-carboxylate (32):

N-Methylmorpholine *N*-oxide (3.5 mg, 0.03 mmol) and OsO_4 (32.5 µL, 1% aq solution) were sequentially added to a solution of **31** (28 mg, 0.025 mmol) in acetone/water (1:1) and the resulting mixture was stirred at room temperature for 12 h before being quenched by addition of saturated aqueous sodium bisulfite (0.1 mL). The resulting mixture was concentrated in vacuo and then diluted with water (10 mL). It was extracted with ethyl acetate (2 × 15 mL) and the combined organic extract was washed successively with water (2 × 20 mL) and brine (20 mL) and dried (Na_2SO_4). After filtration, the filtrate was concentrated in vacuo to provide a crude mass which was roughly purified by chromatography over flash silica using a mixture of petroleum ether/ethyl acetate (1:1) as eluent to provide a mixture (81:19) of isomers **31** as a viscous liquid (20 mg, 68%). IR (KBr): 3436, 1719, 1667, 1605, 1580, 1498 cm^{-1}.^{1}H NMR (400MHz, $CDCl_3$) : δ 7.81 (d, *J*=8.4 Hz, 1H), 7.70 (d, *J* = 8.4 Hz, 2H), 7.41-7.32 (m, 11H), 7.23-7.19 (m, 16H), 7.12-7.02 (m, 9H), 6.90-6.87 (m, 3H), 6.82 (d, *J*=7.2 Hz, 2H), 5.30-5.22 (m, 2H), 5.16-5.08 (m, 4H), 5.00-5.08 (m, 2H), 5.00 (s, 1H), 4.97-4.88 (m, 1H), 4.86-4.79 (m, 3H), 4.75 (d, *J*=2.8 Hz, 1H), 4.71-4.64 (m, 2H), 4.35 (br s, 1H), 4.19 (dd, *J*=14.4, 6 Hz, 1H), 4.04 (d, *J*=15 Hz, 2H), 3.67 (dd, *J* = 15, 6.4 Hz, 1H). ^{13}C NMR (100MHz,$CDCl_3$): δ 191.7, 171.2, 167.2, 166.6, 166.0, 161.4, 158.1, 157.6, 156.2, 137.6, 136.4, 136.3, 135.9, 135.8, 135.3, 132.5, 130.4, 130.1, 128.9, 128.8, 128.7, 128.5, 128.4, 128.3, 128.2, 128.0, 127.9, 127.8, 127.7, 127.5, 127.5, 127.4, 127.3, 127.2, 126.9, 126.3, 123.6, 122.0, 115.5, 114.9, 114.7, 107.1, 78.7, 75.1, 70.5, 70.4, 70.0, 68.4, 68.2, 67.0, 60.4, 52.0, 50.0, 49.9. HRMS (TOF MS ES+): *m/z* calcd for $C_{71}H_{62}N_2NaO_{14}$ (M^++ Na): 1189.4099. Found: 1189.4105.

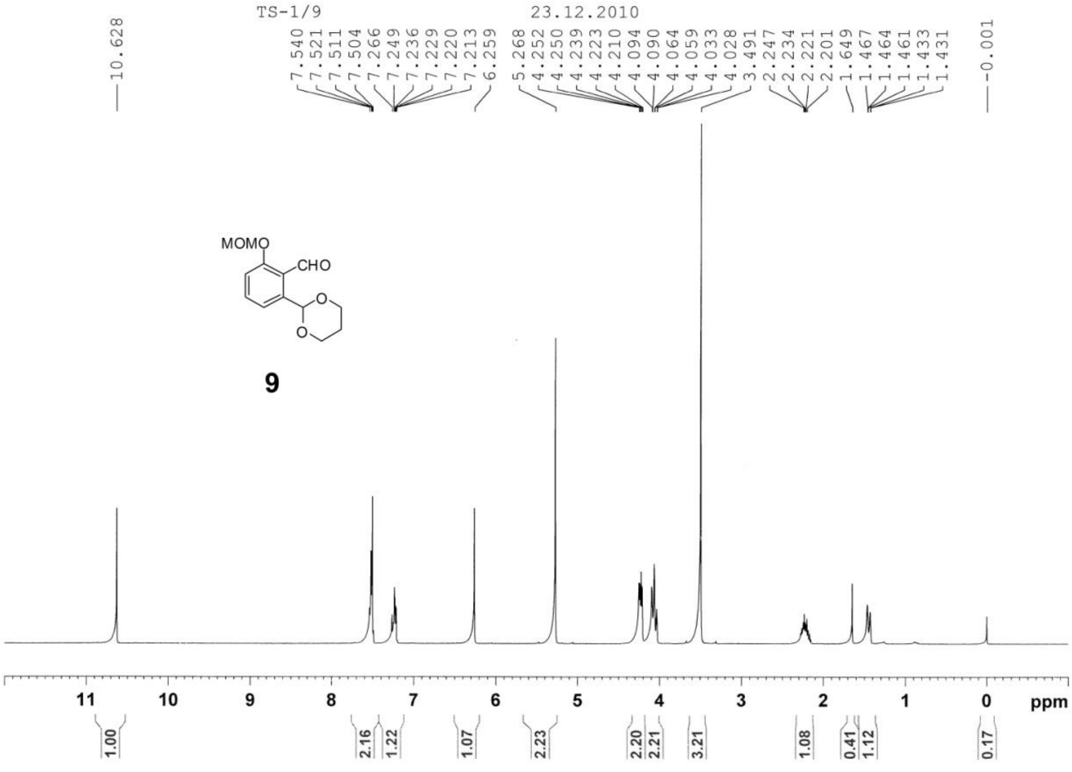
TS-1/9
23.12.2010
10.628
7.540 7.521 7.511 7.504 7.266 7.249 7.236 7.229 7.220 7.213 6.259
5.268 4.252 4.250 4.239 4.223 4.210 4.094 4.090 4.064 4.059 4.033 4.028 3.491
2.247 2.234 2.221 2.201 1.649 1.467 1.464 1.461 1.433 1.431
-0.001
MOMO
CHO
O
O
9
ppm
1.00 2.16 1.22 1.07 2.23 2.20 2.21 3.21 1.08 0.41 1.12 0.17

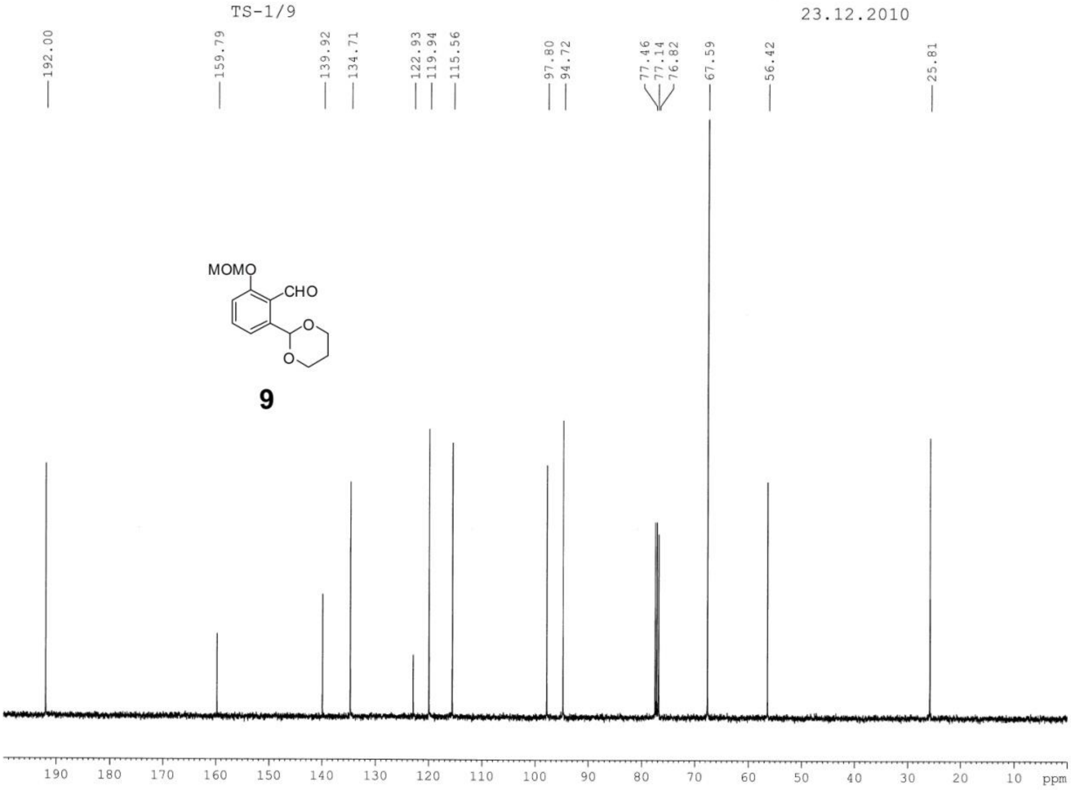
TS-1/9
23.12.2010
192.00
159.79
139.92 134.71
122.93 119.94 115.56
97.80 94.72
77.46 77.14 76.82 67.59
56.42
25.81
MOMO
CHO
O
O
9
ppm

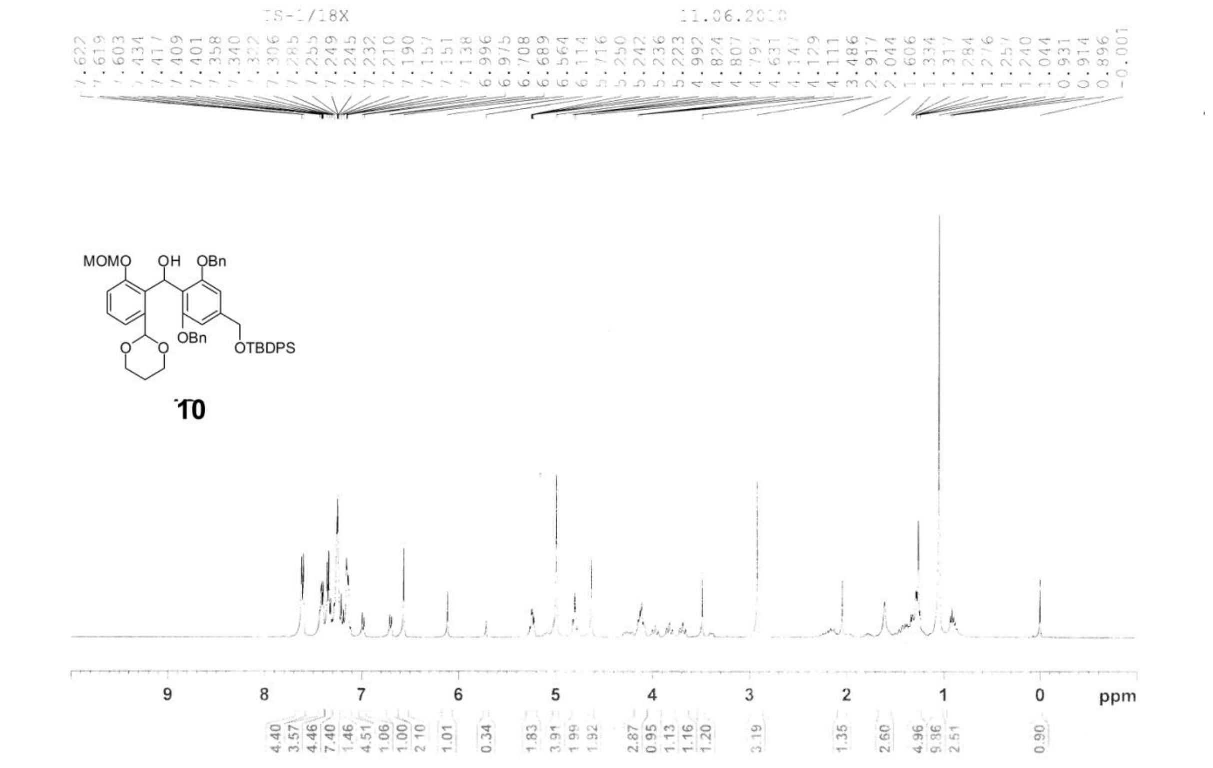
TS-1/18X
11.06.2010
7.622 7.619 7.603 7.434 7.417 7.409 7.401 7.358 7.340 7.322 7.306 7.285 7.255 7.249 7.245 7.232 7.210 7.190 7.157 7.151 7.138 6.996 6.975 6.708 6.689 6.564 6.114 5.716 5.250 5.242 5.236 5.223 4.992 4.824 4.807 4.797 4.631 4.147 4.129 4.111 3.486 2.917 2.044 1.606 1.334 1.317 1.284 1.276 1.257 1.240 1.044 0.931 0.914 0.896 -0.001
MOMO
OH
OBn
OBn
OTBDPS
10
ppm

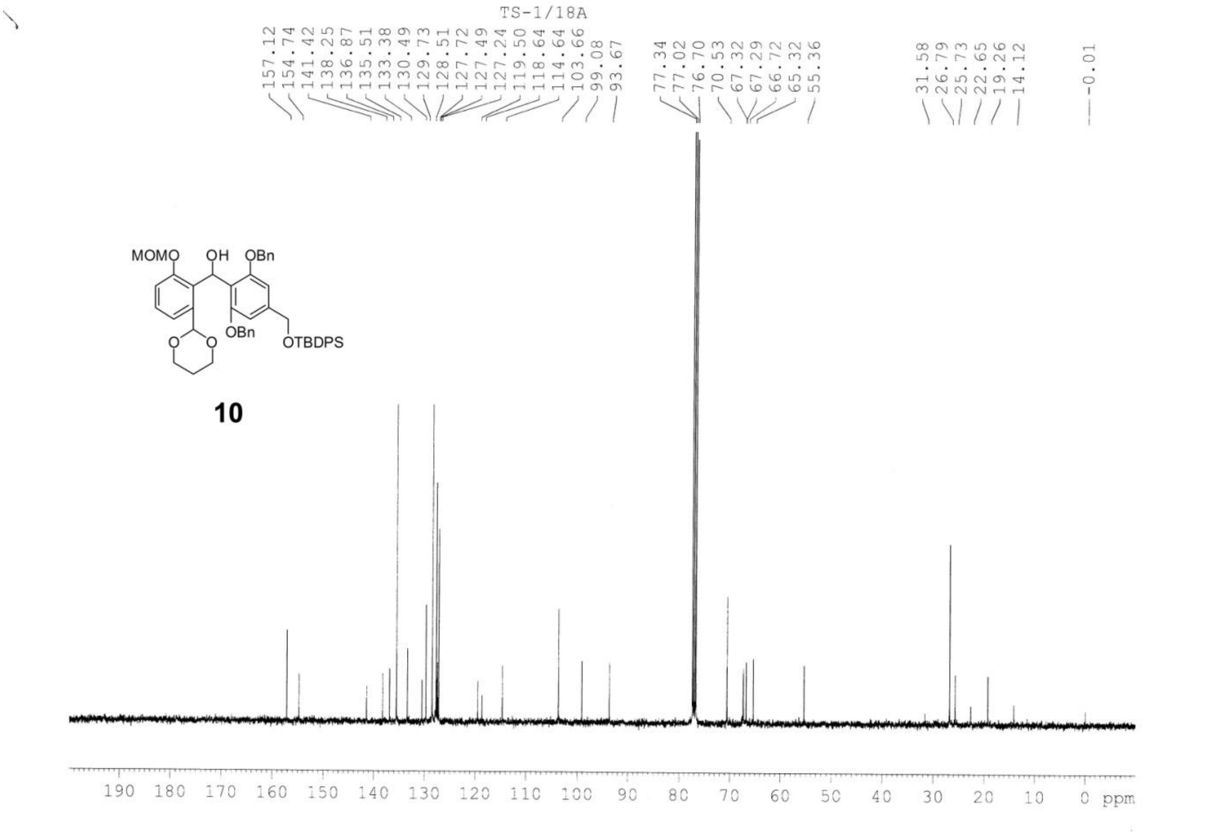
TS-1/18A
157.12 154.74 141.42 138.25 136.87 135.51 133.38 130.49 129.73 128.51 127.72 127.49 127.24 119.50 118.64 114.64 103.66 99.08 93.67
77.34 77.02 76.70 70.53 67.32 67.29 66.72 65.32 55.36
31.58 26.79 25.73 22.65 19.26 14.12
-0.01
MOMO
OH
OBn
OBn
OTBDPS
10
190 180 170 160 150 140 130 120 110 100 90 80 70 60 50 40 30 20 10 0 ppm

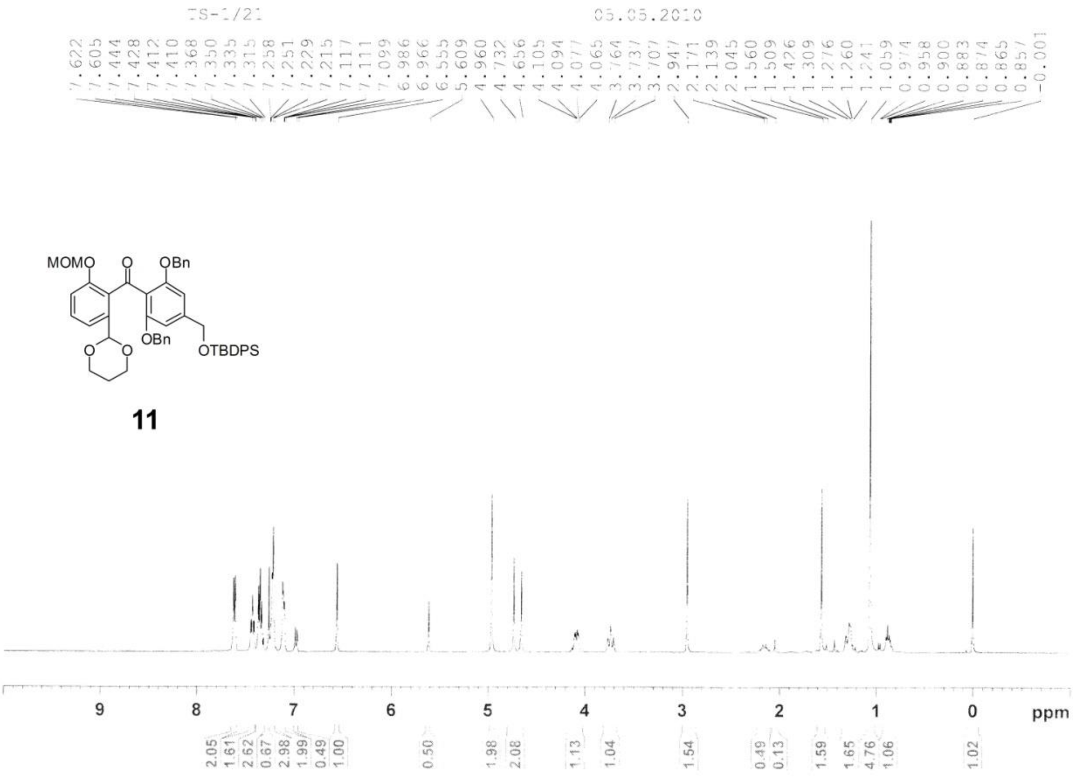
TS-1/21
MOMO
OBn
OBn
OTBDPS
ppm

11

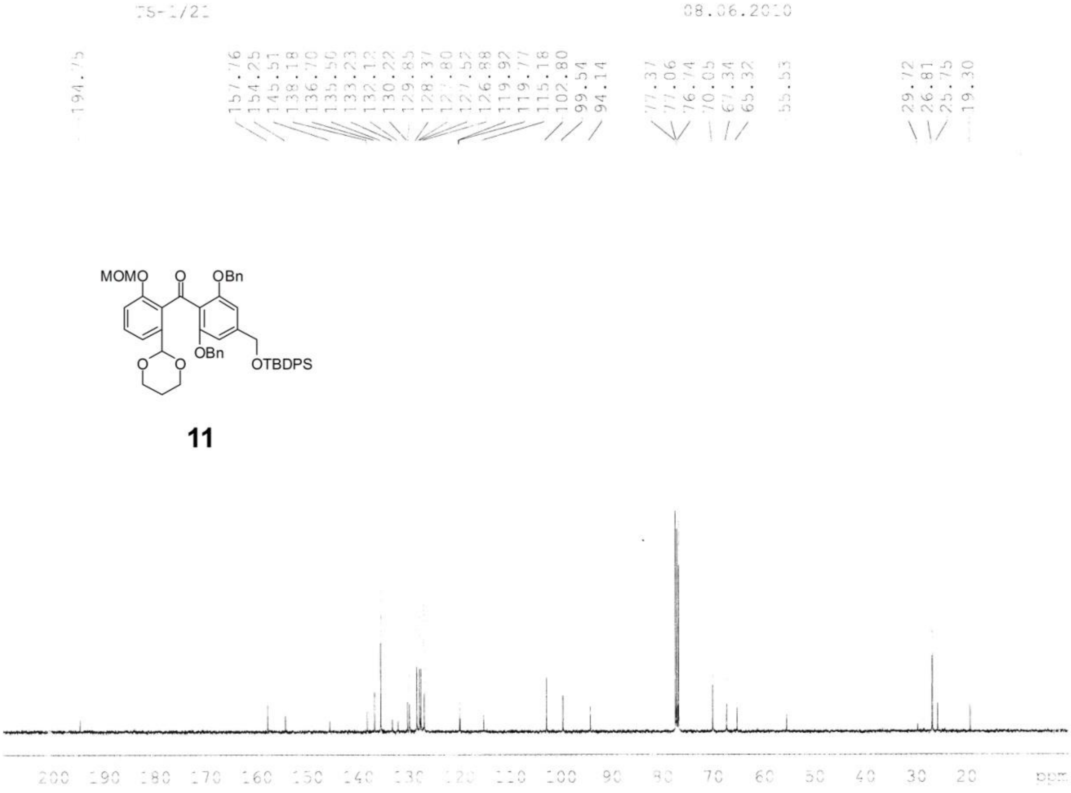
TS-1/21
08.06.2010
MOMO
OBn
OBn
OTBDPS
ppm

11

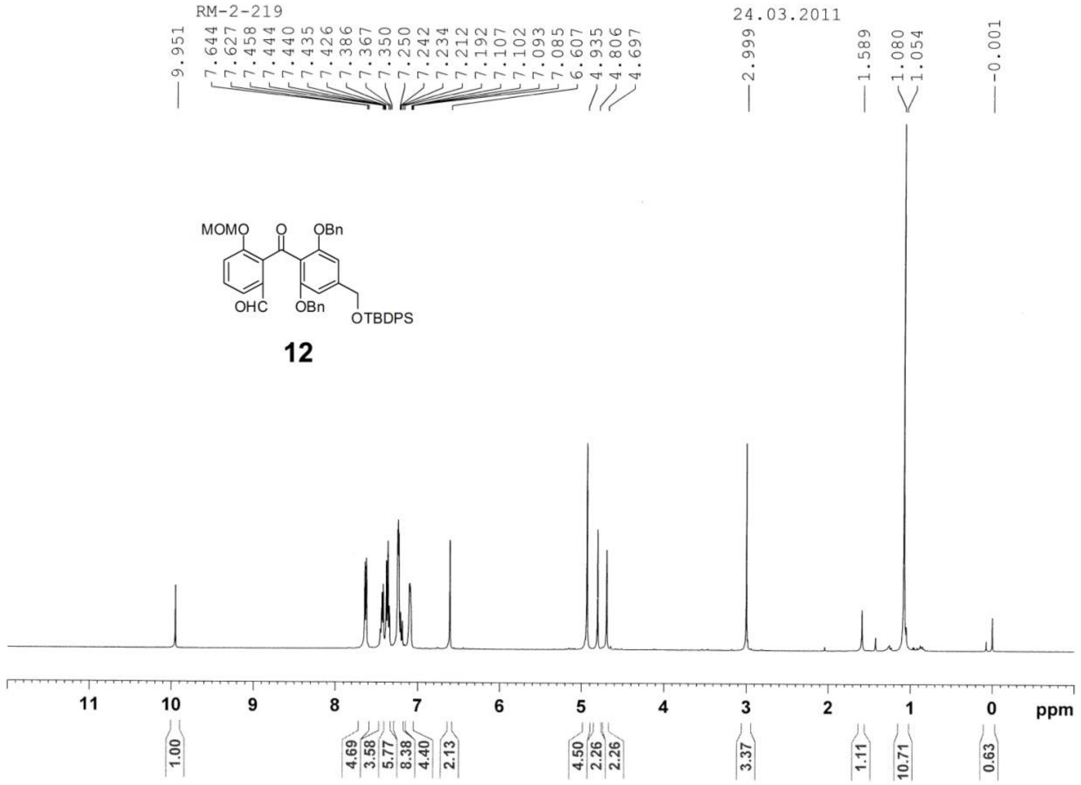
RM-2-219
24.03.2011
9.951
7.644 7.627 7.458 7.444 7.440 7.435 7.426 7.386 7.367 7.350 7.250 7.242 7.234 7.212 7.192 7.107 7.102 7.093 7.085 6.607 4.935 4.806 4.697
2.999
1.589
1.080 1.054
-0.001
MOMO
O
OBn
OHC
OBn
OTBDPS
12
11 10 9 8 7 6 5 4 3 2 1 0 ppm
1.00
4.69 3.58 5.77 8.38 4.40 2.13
4.50 2.26 2.26
3.37
1.11
10.71
0.63

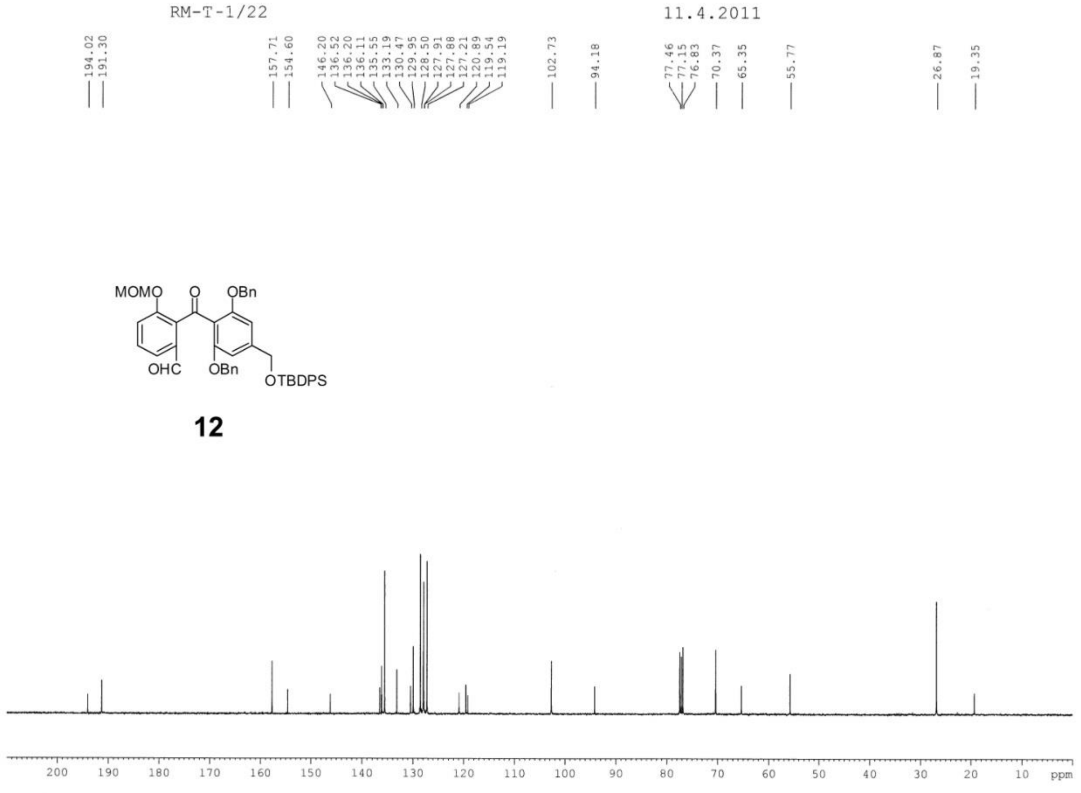
RM-T-1/22
11.4.2011
194.02 191.30
157.71 154.60
146.20 136.52 136.20 136.11 135.55 133.19 130.47 129.95 128.50 127.91 127.88 127.21 120.89 119.54 119.19
102.73
94.18
77.46 77.15 76.83 70.37 65.35
55.77
26.87
19.35
MOMO
O
OBn
OHC
OBn
OTBDPS
12
200 190 180 170 160 150 140 130 120 110 100 90 80 70 60 50 40 30 20 10 ppm

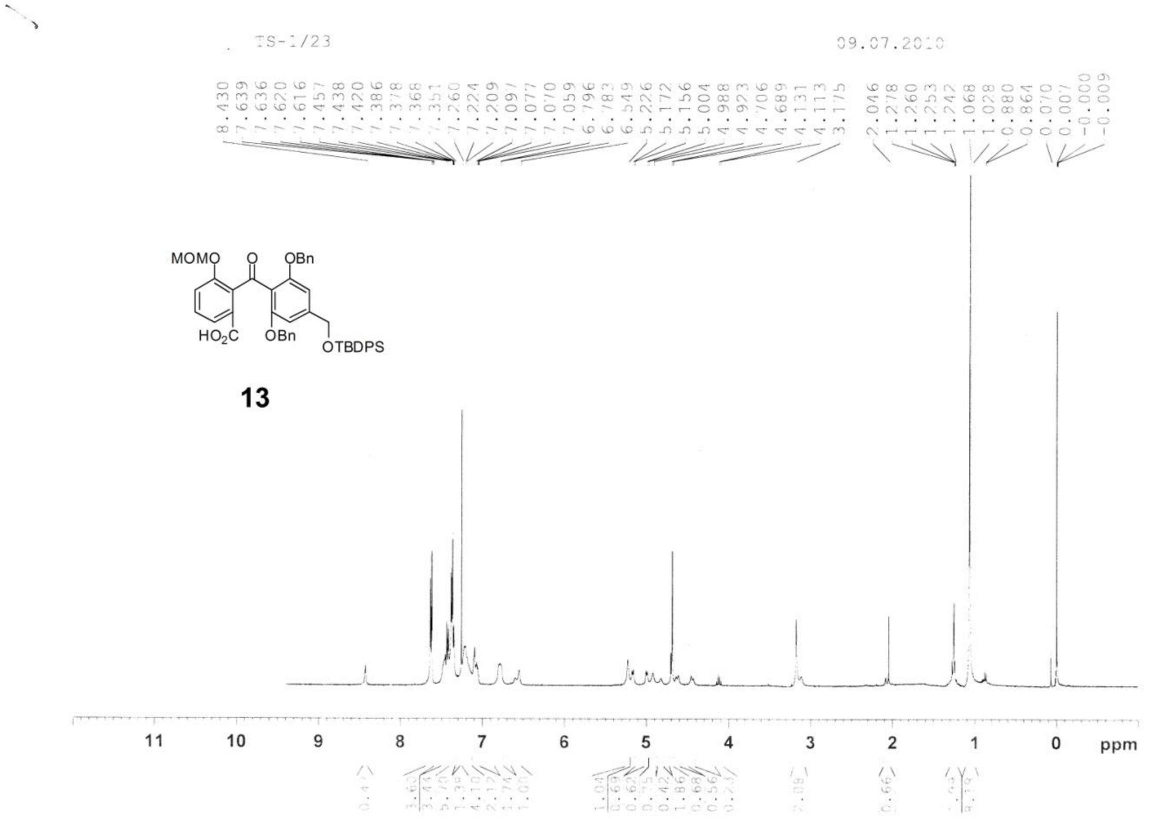
TS-1/23
09.07.2010
8.430 7.639 7.636 7.620 7.616 7.457 7.438 7.420 7.386 7.378 7.368 7.351 7.260 7.224 7.209 7.097 7.077 7.070 7.059 6.796 6.783 6.549 5.226 5.172 5.156 5.004 4.988 4.923 4.706 4.689 4.131 4.113 3.175 2.046 1.278 1.260 1.253 1.242 1.068 1.028 0.880 0.864 0.070 0.007 -0.000 -0.009
MOMO
O
OBn
HO2C
OBn
OTBDPS
13
11 10 9 8 7 6 5 4 3 2 1 0 ppm

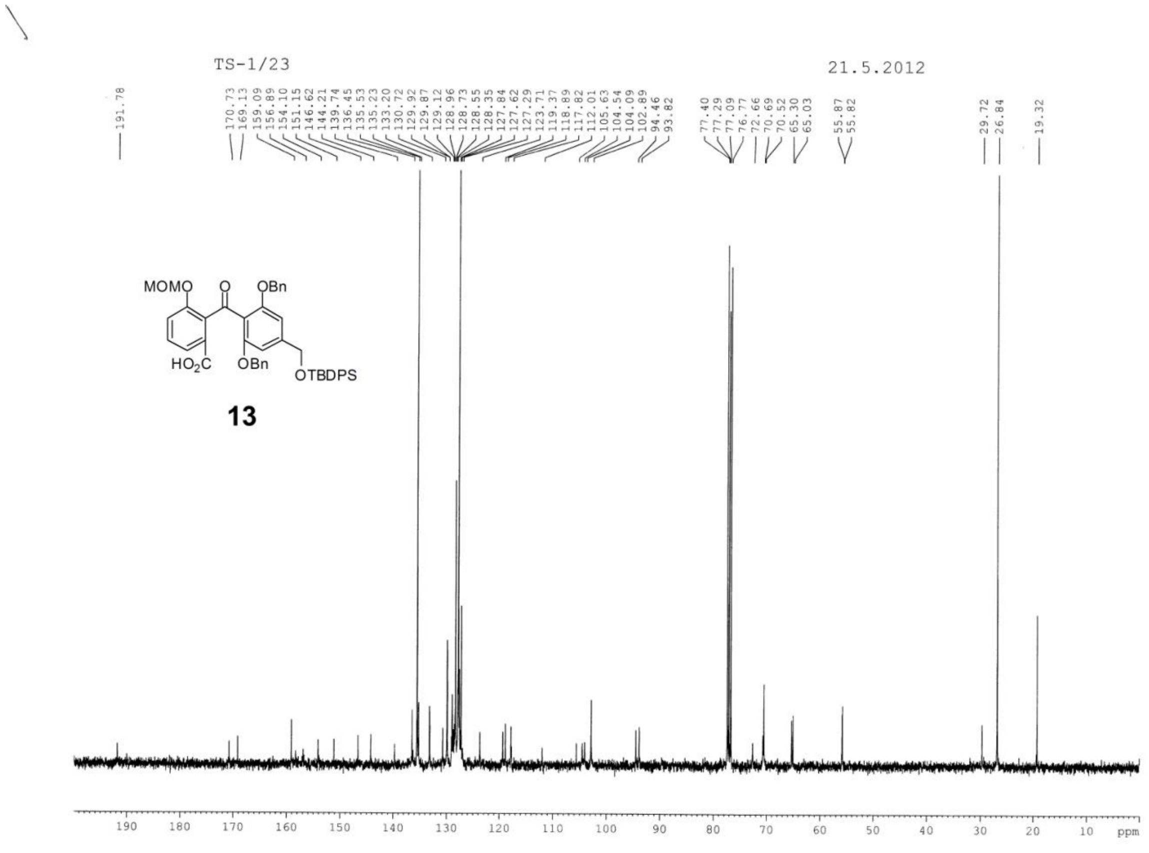
TS-1/23
21.5.2012
191.78
170.73 169.13 159.09 156.89 154.10 151.15 146.62 144.21 139.74 136.45 135.53 135.23 133.20 130.72 129.92 129.87 129.12 128.96 128.73 128.55 128.35 127.84 127.62 127.29 123.71 119.37 118.89 117.82 112.01 105.63 104.54 104.09 102.89 94.46 93.82
77.40 77.29 77.09 76.77 72.66 70.69 70.52 65.30 65.03
55.87 55.82
29.72 26.84
19.32
MOMO
O
OBn
HO2C
OBn
OTBDPS
13
190 180 170 160 150 140 130 120 110 100 90 80 70 60 50 40 30 20 10 ppm

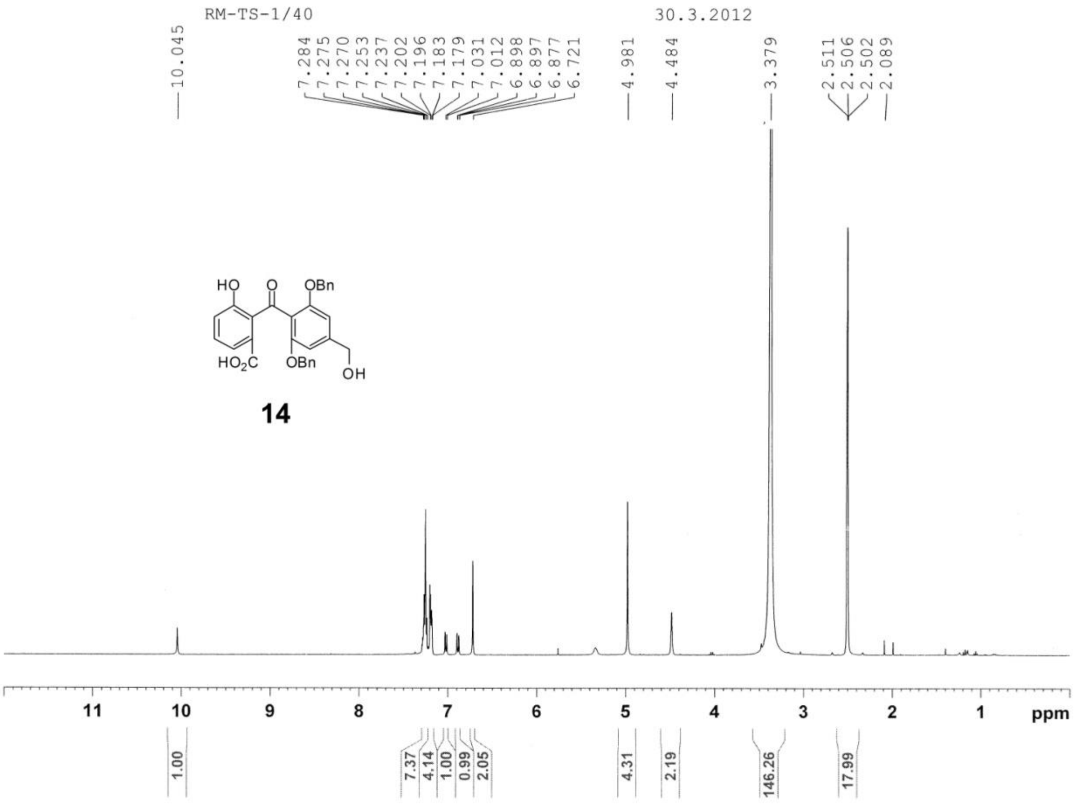
RM-TS-1/40
30.3.2012
10.045
7.284
7.275
7.270
7.253
7.237
7.202
7.196
7.183
7.179
7.031
7.012
6.898
6.897
6.877
6.721
4.981
4.484
3.379
2.511
2.506
2.502
2.089
HO
O
OBn
HO2C
OBn
OH
14
11
10
9
8
7
6
5
4
3
2
1
ppm
1.00
7.37
4.14
1.00
0.99
2.05
4.31
2.19
146.26
17.99

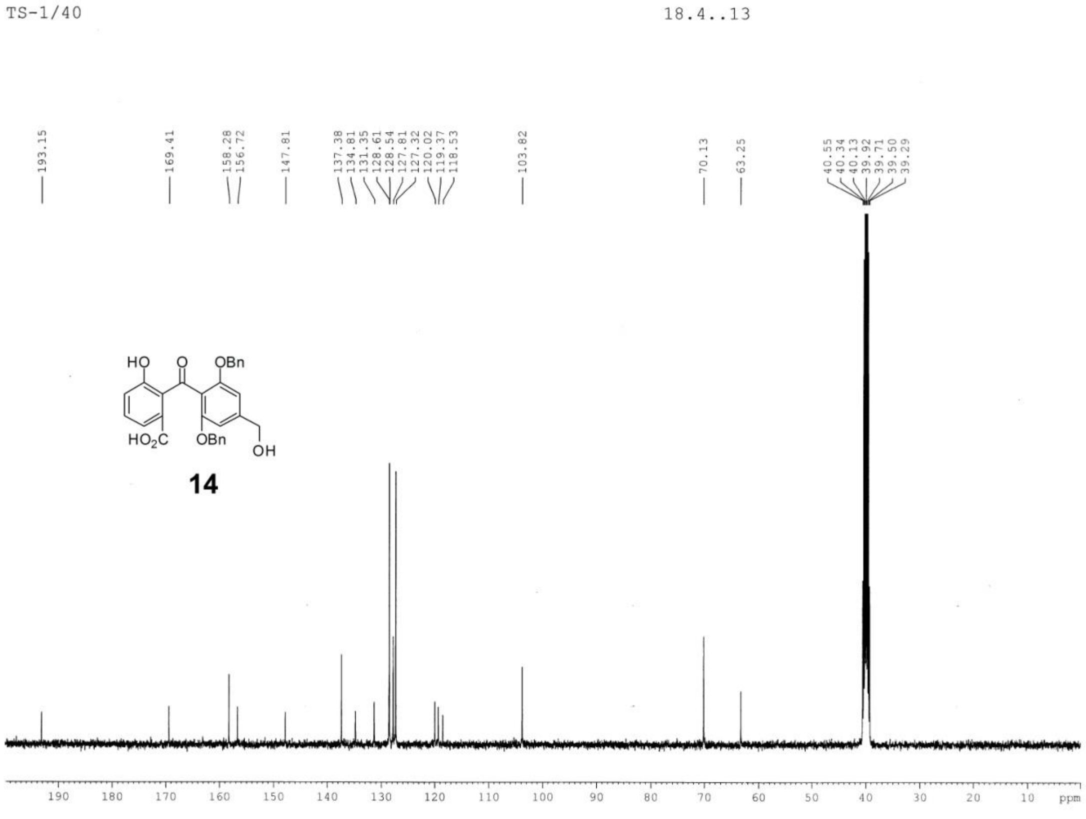
TS-1/40
18.4..13
193.15
169.41
158.28
156.72
147.81
137.38
134.81
131.35
128.61
128.54
127.81
127.32
120.02
119.37
118.53
103.82
70.13
63.25
40.55
40.34
40.13
39.92
39.71
39.50
39.29
HO
O
OBn
HO2C
OBn
OH
14
190
180
170
160
150
140
130
120
110
100
90
80
70
60
50
40
30
20
10
ppm

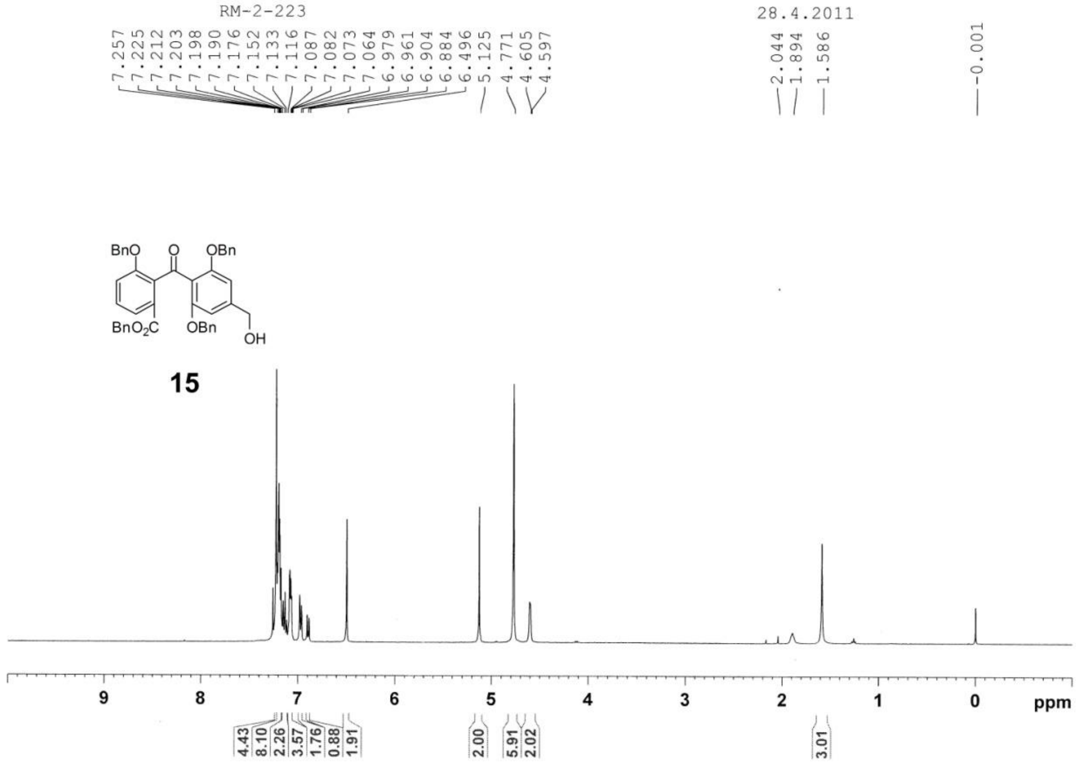
RM-2-223
28.4.2011
7.257 7.225 7.212 7.203 7.198 7.190 7.176 7.152 7.133 7.116 7.087 7.082 7.073 7.064 6.979 6.961 6.904 6.884 6.496 5.125 4.771 4.605 4.597
2.044 1.894 1.586
-0.001
BnO O OBn
BnO2C OBn OH
15
9 8 7 6 5 4 3 2 1 0 ppm
4.43 8.10 2.26 3.57 1.76 0.88 1.91 2.00 5.91 2.02 3.01

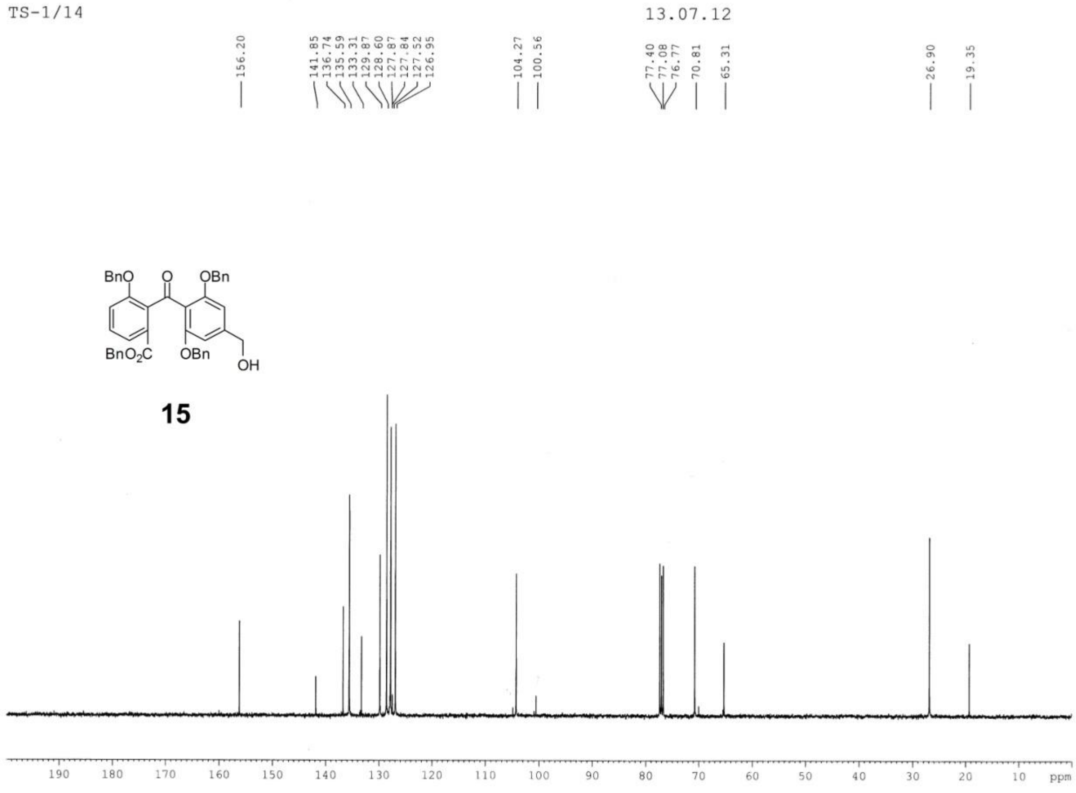
TS-1/14
13.07.12
156.20
141.85 136.74 135.59 133.31 129.87 128.60 127.87 127.84 127.52 126.95
104.27 100.56
77.40 77.08 76.77 70.81 65.31
26.90 19.35
BnO O OBn
BnO2C OBn OH
15
190 180 170 160 150 140 130 120 110 100 90 80 70 60 50 40 30 20 10 ppm

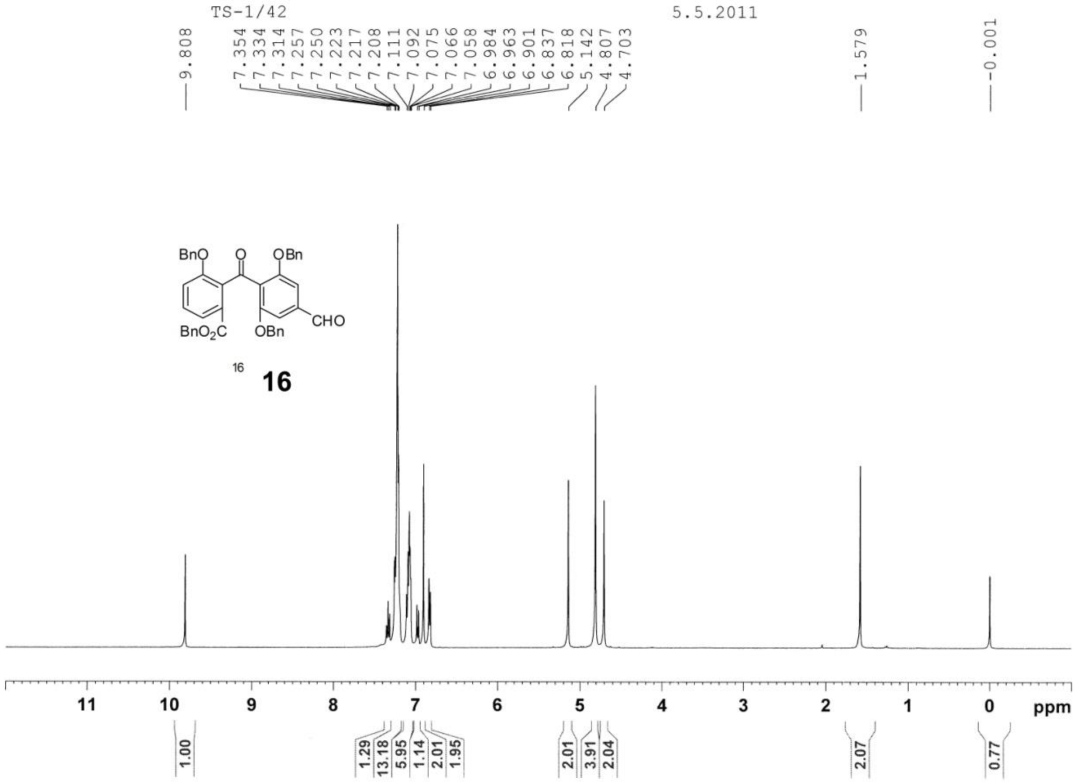
TS-1/42
5.5.2011
9.808
7.354
7.334
7.314
7.257
7.250
7.223
7.217
7.208
7.111
7.092
7.075
7.066
7.058
6.984
6.963
6.901
6.837
6.818
5.142
4.807
4.703
1.579
-0.001
BnO
O
OBn
BnO2C
OBn
CHO
16
16
11
10
9
8
7
6
5
4
3
2
1
0
ppm
1.00
1.29
13.18
5.95
1.14
2.01
1.95
2.01
3.91
2.04
2.07
0.77

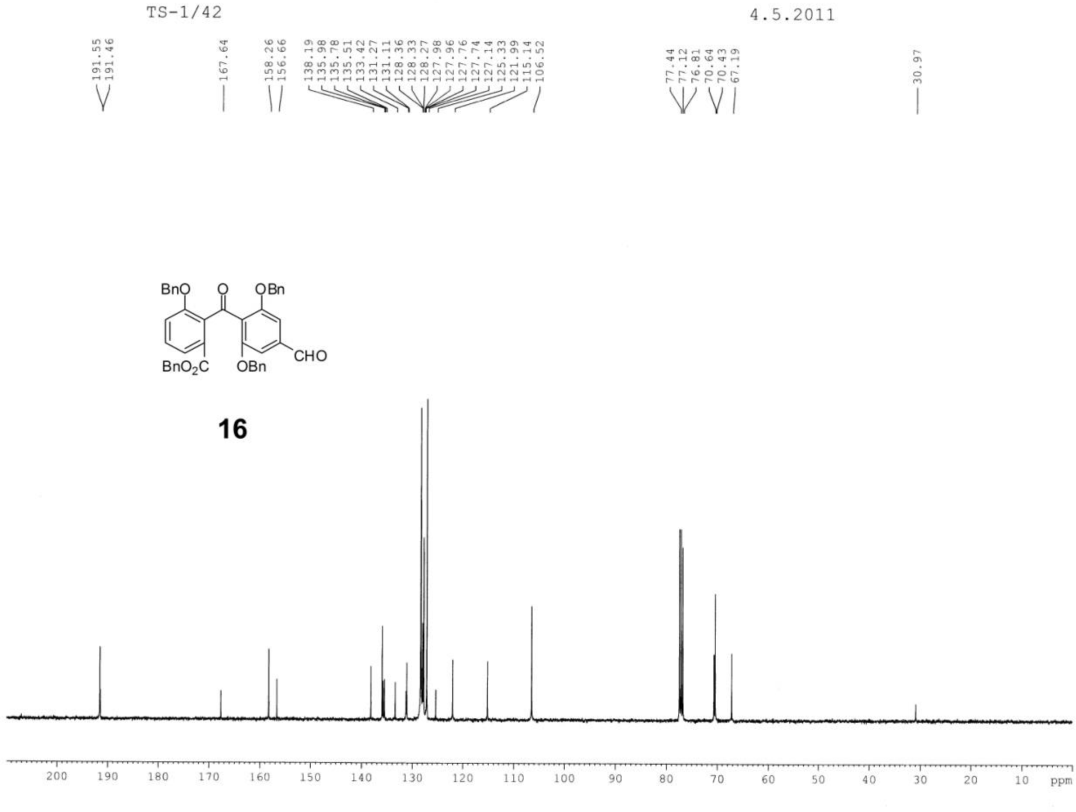
TS-1/42
4.5.2011
191.55
191.46
167.64
158.26
156.66
138.19
135.98
135.78
135.51
133.42
131.27
131.11
128.36
128.33
128.27
127.98
127.96
127.76
127.74
127.14
125.33
121.99
115.14
106.52
77.44
77.12
76.81
70.64
70.43
67.19
30.97
BnO
O
OBn
BnO2C
OBn
CHO
16
200
190
180
170
160
150
140
130
120
110
100
90
80
70
60
50
40
30
20
10
ppm

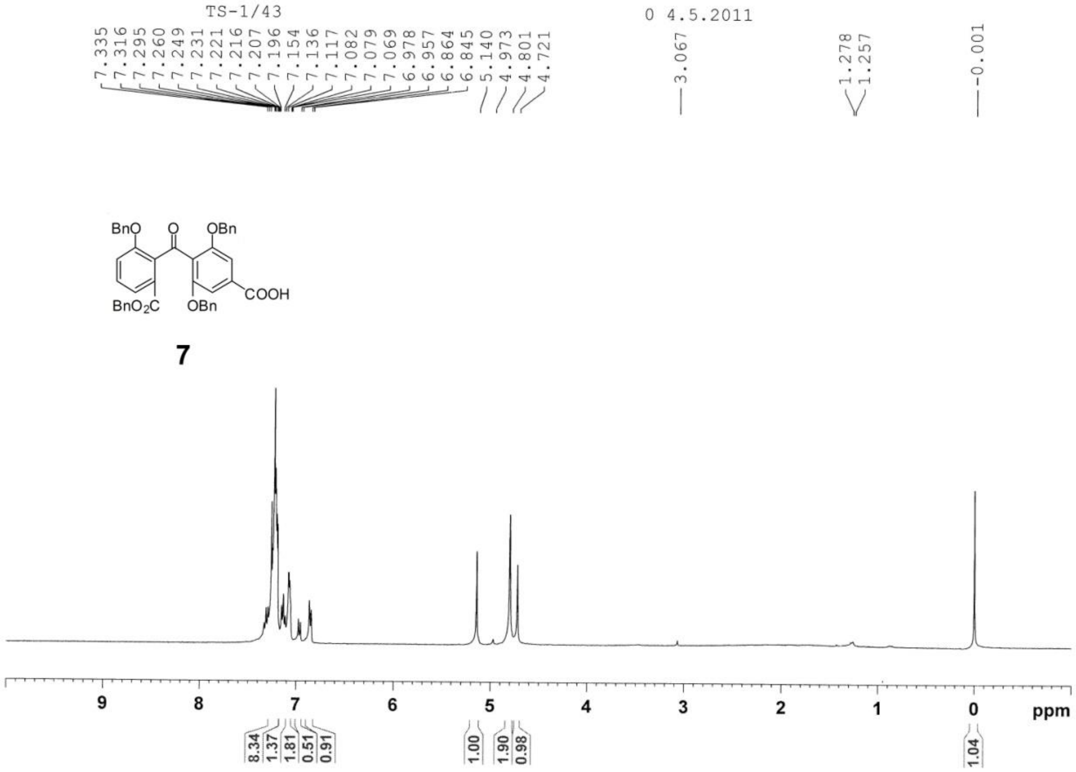
TS-1/43
0 4.5.2011
7.335 7.316 7.295 7.260 7.249 7.231 7.221 7.216 7.207 7.196 7.154 7.136 7.117 7.082 7.079 7.069 6.978 6.957 6.864 6.845 5.140 4.973 4.801 4.721
3.067
1.278 1.257
-0.001
BnO O OBn
BnO2C OBn COOH
7
9 8 7 6 5 4 3 2 1 0 ppm
8.34 1.37 1.81 0.51 0.91
1.00 1.90 0.98
1.04

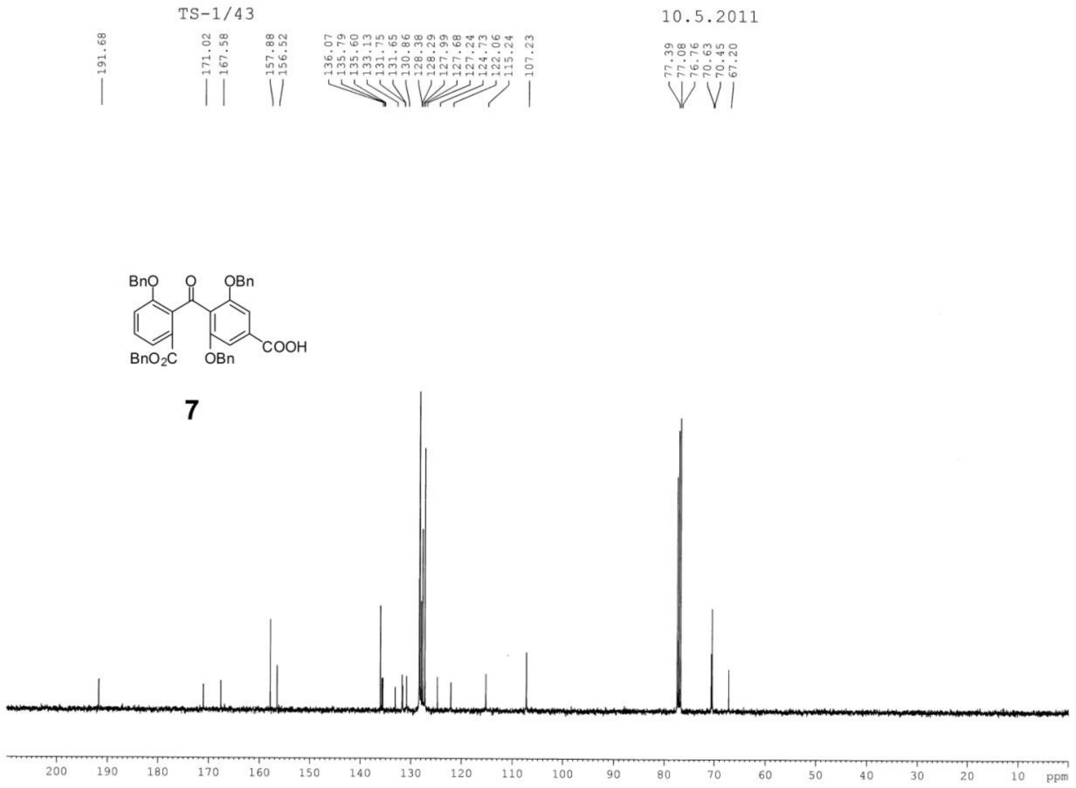
TS-1/43
10.5.2011
191.68
171.02 167.58
157.88 156.52
136.07 135.79 135.60 133.13 131.75 131.65 130.86 128.38 128.29 127.99 127.68 127.24 124.73 122.06 115.24
107.23
77.39 77.08 76.76 70.63 70.45 67.20
BnO O OBn
BnO2C OBn COOH
7
200 190 180 170 160 150 140 130 120 110 100 90 80 70 60 50 40 30 20 10 ppm

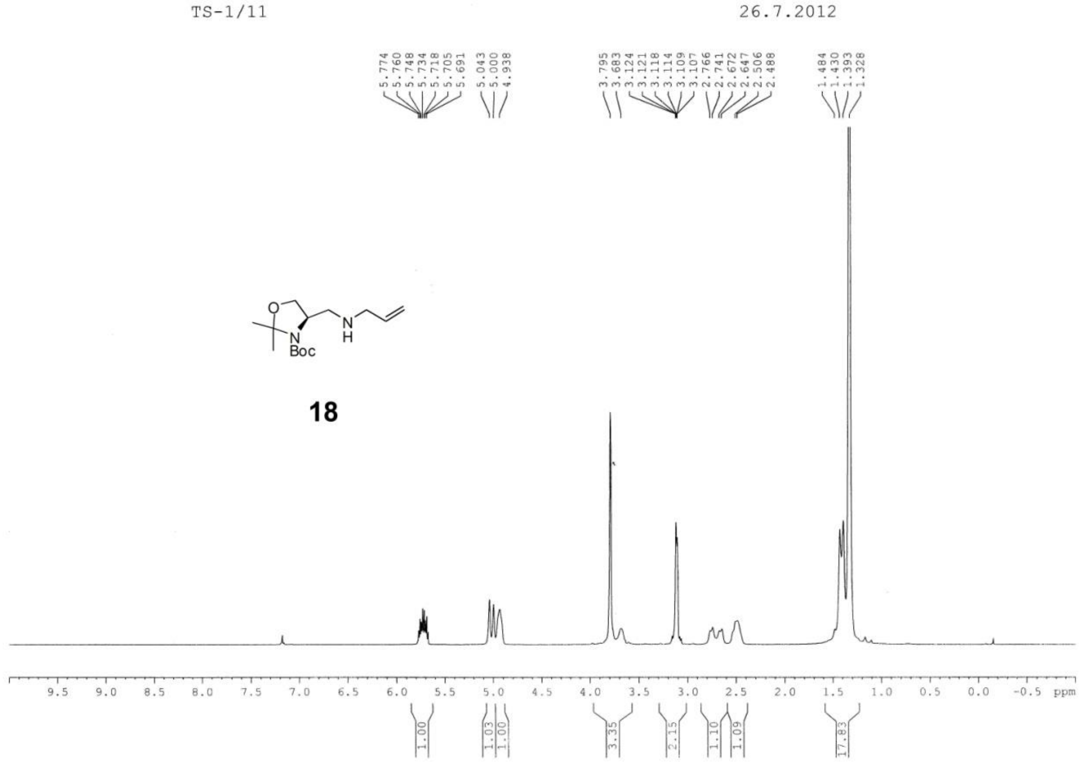
TS-1/11
26.7.2012
5.774 5.760 5.748 5.734 5.718 5.705 5.691
5.043 5.000 4.938
3.795 3.683 3.124 3.121 3.118 3.114 3.109 3.107 2.766 2.741 2.672 2.647 2.506 2.488
1.484 1.430 1.393 1.328
N H
O
N
Boc
18
9.5 9.0 8.5 8.0 7.5 7.0 6.5 6.0 5.5 5.0 4.5 4.0 3.5 3.0 2.5 2.0 1.5 1.0 0.5 0.0 -0.5 ppm
1.00 1.03 1.00 3.35 2.15 1.10 1.09 17.83

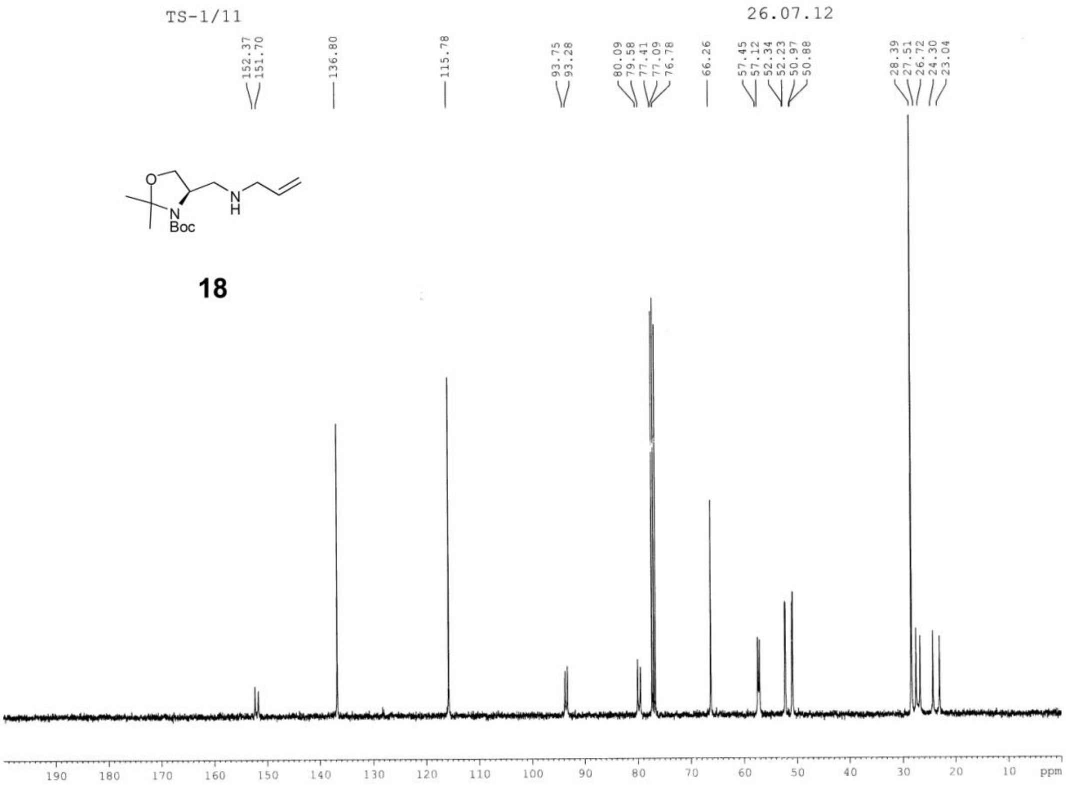
TS-1/11
26.07.12
152.37 151.70
136.80
115.78
93.75 93.28
80.09 79.58 77.41 77.09 76.78
66.26
57.45 57.12 52.34 52.23 50.97 50.88
28.39 27.51 26.72 24.30 23.04
N H
O
N
Boc
18
190 180 170 160 150 140 130 120 110 100 90 80 70 60 50 40 30 20 10 ppm

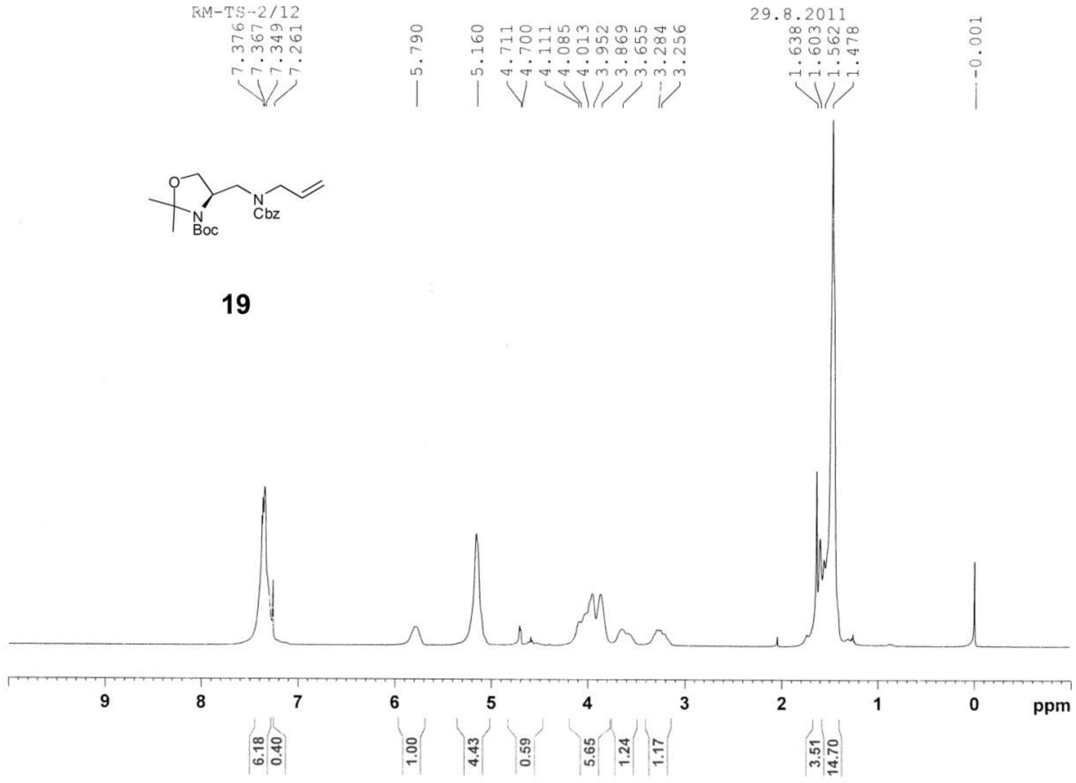
O
N
Boc
N
Cbz
19
RM-TS-2/12
29.8.2011
7.376
7.367
7.349
7.261
5.790
5.160
4.711
4.700
4.111
4.085
4.013
3.952
3.869
3.655
3.284
3.256
1.638
1.603
1.562
1.478
-0.001
9
8
7
6
5
4
3
2
1
0
ppm
6.18
0.40
1.00
4.43
0.59
5.65
1.24
1.17
3.51
14.70

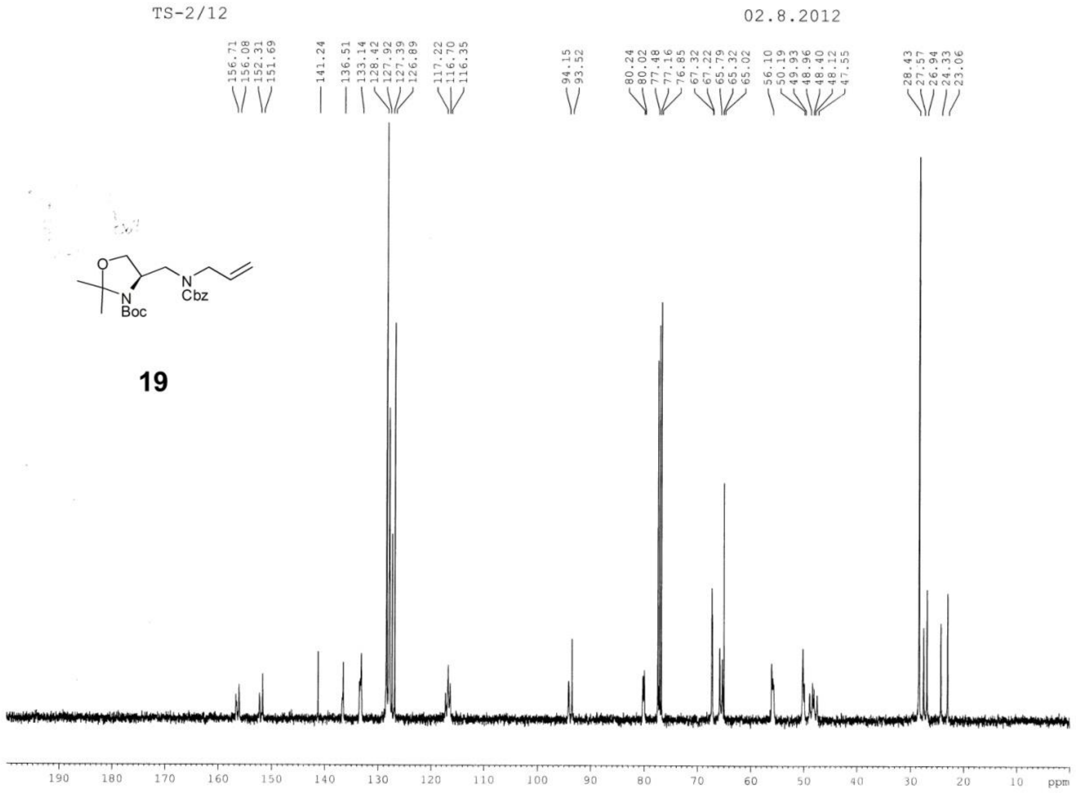
O
N
Boc
N
Cbz
19
TS-2/12
02.8.2012
156.71
156.08
152.31
151.69
141.24
136.51
133.14
128.42
127.92
127.39
126.89
117.22
116.70
116.35
94.15
93.52
80.24
80.02
77.48
77.16
76.85
67.32
67.22
65.79
65.32
65.02
56.10
50.19
49.93
48.96
48.40
48.12
47.55
28.43
27.57
26.94
24.33
23.06
190
180
170
160
150
140
130
120
110
100
90
80
70
60
50
40
30
20
10
ppm

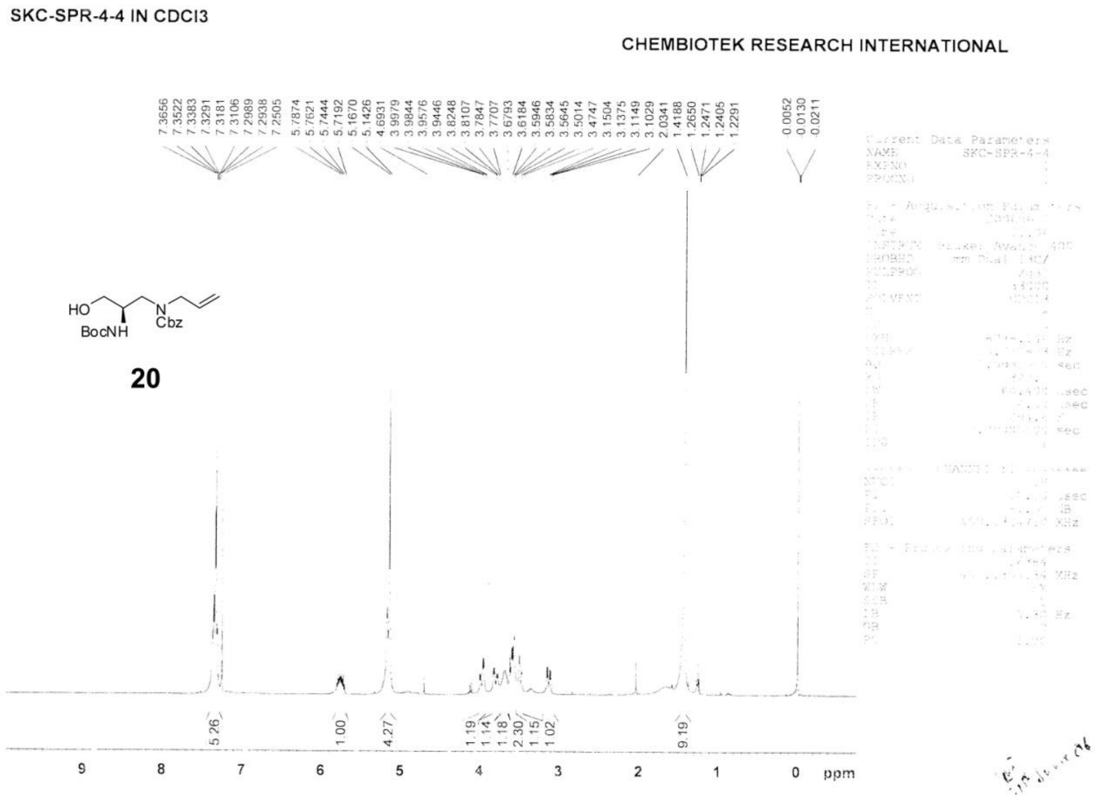
SKC-SPR-4-4 IN CDCl3
CHEMBIOTEK RESEARCH INTERNATIONAL
7.3656 7.3522 7.3383 7.3291 7.3181 7.3106 7.2989 7.2938 7.2505 5.7874 5.7621 5.7444 5.7192 5.1670 5.1426 4.6931 3.9979 3.9844 3.9576 3.9446 3.8248 3.8107 3.7847 3.7707 3.6793 3.6184 3.5946 3.5834 3.5645 3.5014 3.4747 3.1504 3.1375 3.1149 3.1029 2.0341 1.4188 1.2650 1.2471 1.2405 1.2291 0.0052 -0.0130 -0.0211
HO
BocNH
N
Cbz
20
5.26
1.00
4.27
1.19
1.14
1.18
2.30
1.15
1.02
9.19
9 8 7 6 5 4 3 2 1 0 ppm

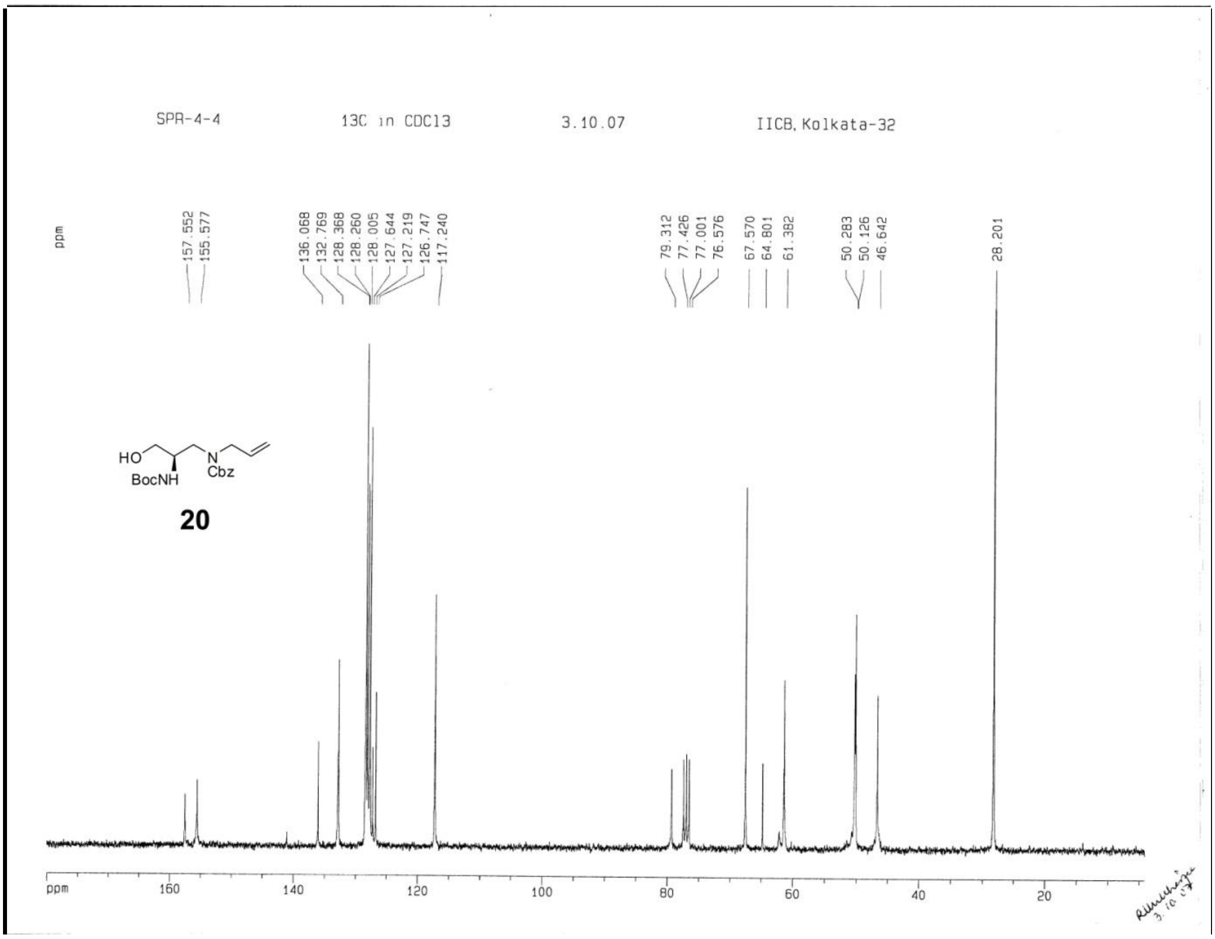
SPR-4-4
13C in CDCl3
3.10.07
IICB, Kolkata-32
157.552 155.577 136.068 132.769 128.368 128.260 128.005 127.644 127.219 126.747 117.240 79.312 77.426 77.001 76.576 67.570 64.801 61.382 50.283 50.126 46.642 28.201
HO
BocNH
N
Cbz
20
ppm 160 140 120 100 80 60 40 20

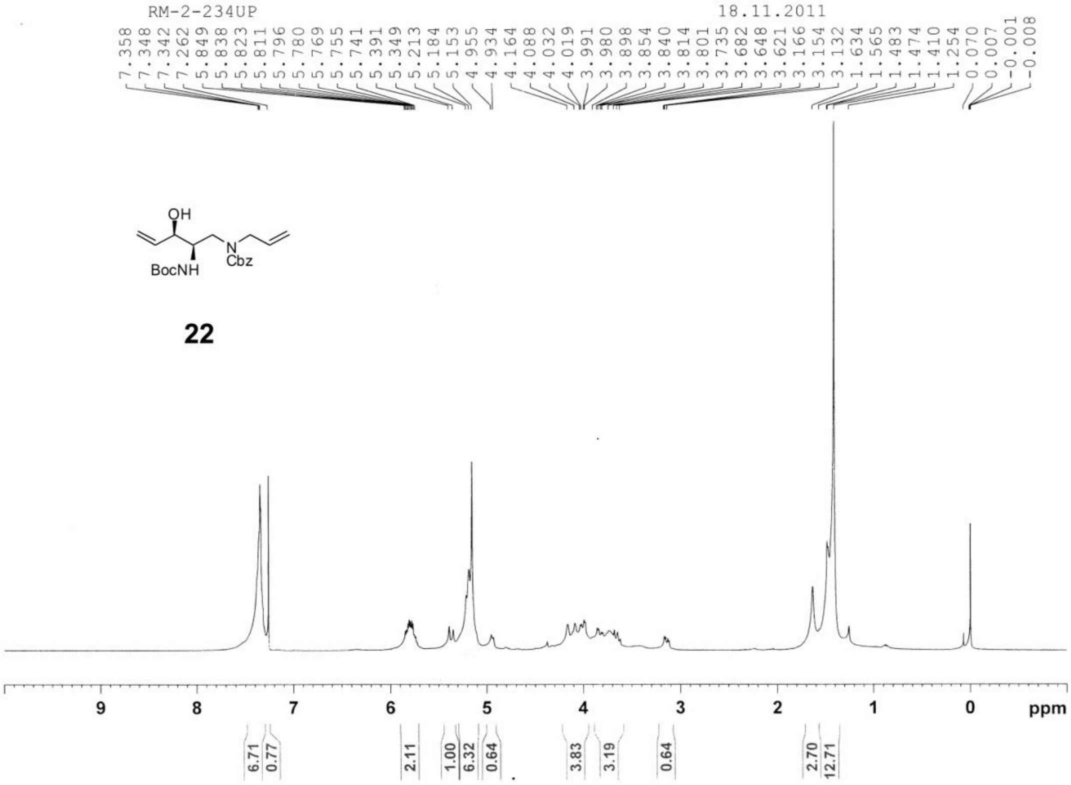
RM-2-234UP
18.11.2011
7.358
7.348
7.342
7.262
5.849
5.838
5.823
5.811
5.796
5.780
5.769
5.755
5.741
5.391
5.349
5.213
5.184
5.153
4.955
4.934
4.164
4.088
4.032
4.019
3.991
3.980
3.898
3.854
3.840
3.814
3.801
3.735
3.682
3.648
3.621
3.166
3.154
3.132
1.634
1.565
1.483
1.474
1.410
1.254
0.070
0.007
-0.001
-0.008
OH
N
Cbz
BocNH
22
ppm
6.71
0.77
2.11
1.00
6.32
0.64
3.83
3.19
0.64
2.70
12.71

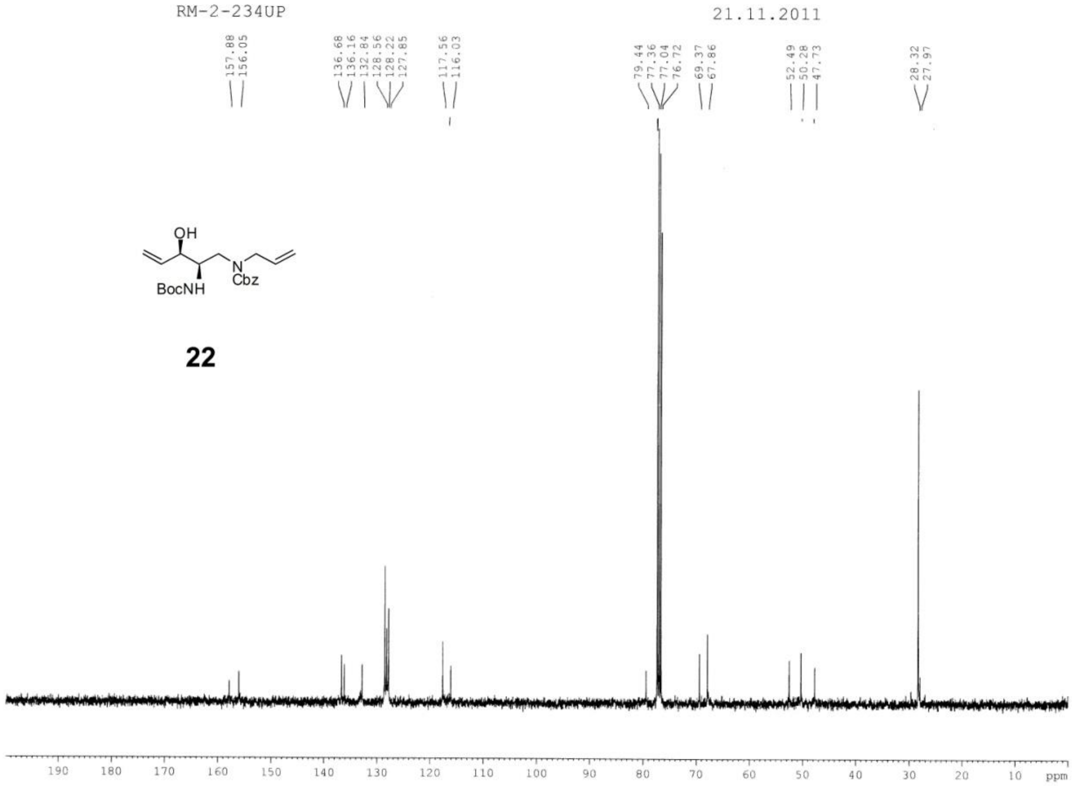
RM-2-234UP
21.11.2011
157.88
156.05
136.68
136.16
132.84
128.56
128.22
127.85
117.56
116.03
79.44
77.36
77.04
76.72
69.37
67.86
52.49
50.28
47.73
28.32
27.97
OH
N
Cbz
BocNH
22
ppm

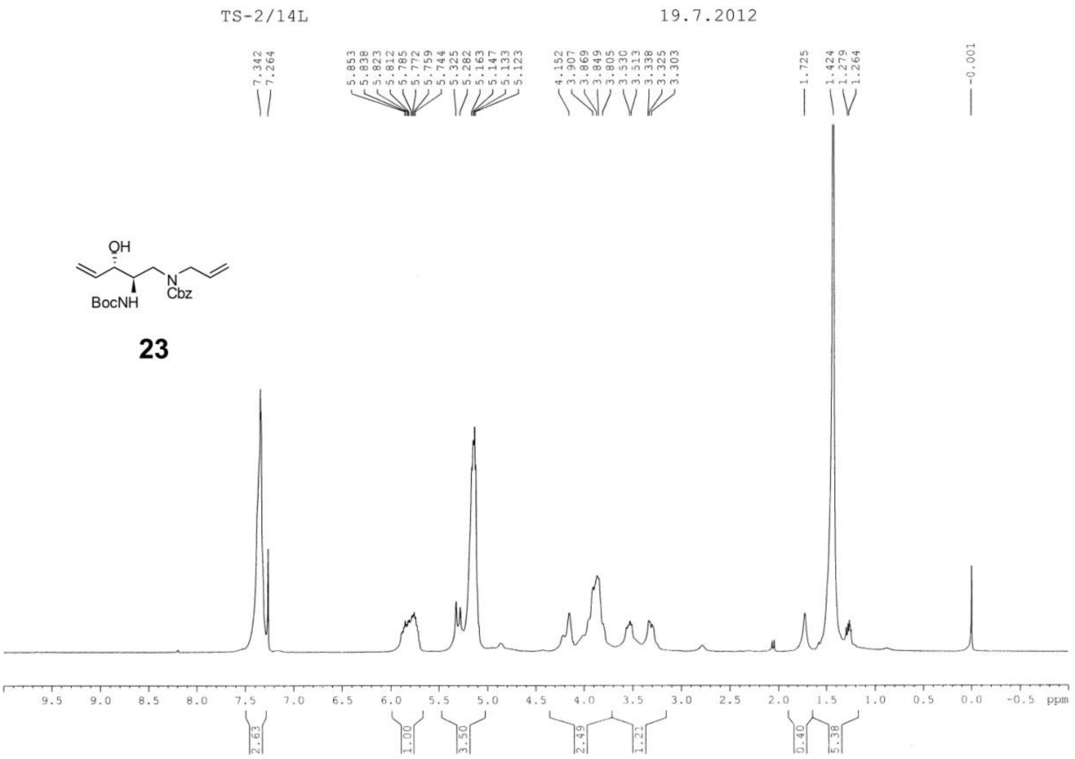

TS-2/14L
19.7.2012
BocNH
Cbz
23
ppm

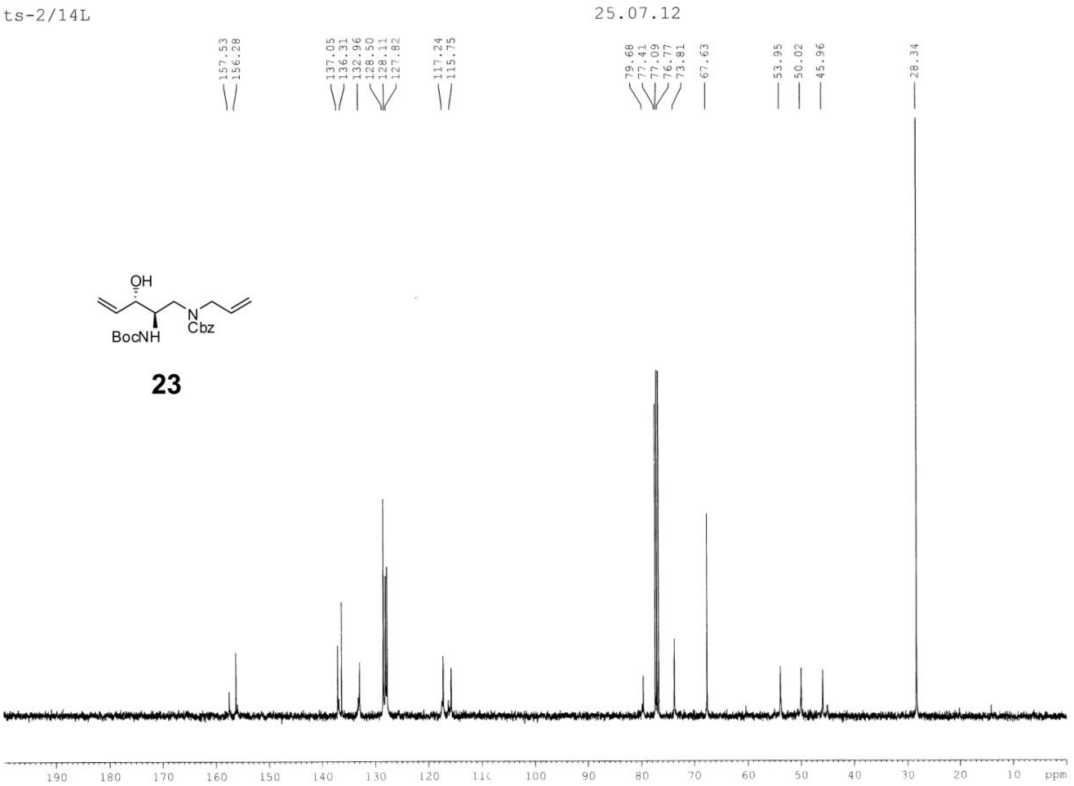

ts-2/14L
25.07.12
BocNH
Cbz
23
ppm

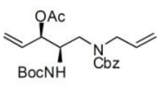

24

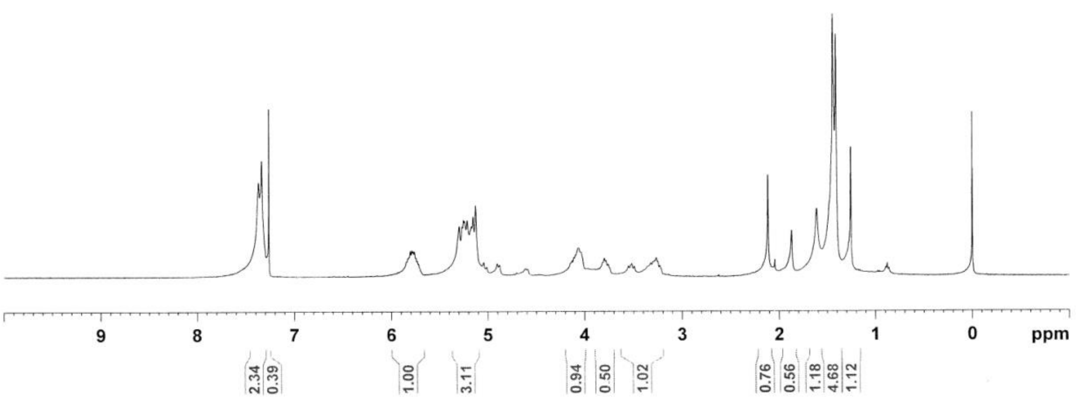
RM-2-235U
05.12.2011
7.521 7.370 7.363 7.336 7.285 7.263 5.806 5.790 5.779 5.765 5.734 5.721 5.297 5.268 5.254 5.241 5.214 5.171 5.154 5.126 5.044 5.014 4.905 4.881 4.617 4.592 4.130 4.112 4.061 3.901 3.854 3.799 3.783 3.762 3.553 3.518 3.494 3.324 3.268 3.251 3.230 2.115 2.046 1.871 1.611 1.438 1.405 1.253 0.896 0.879 0.862 -0.001
9 8 7 6 5 4 3 2 1 0 ppm
2.34 0.39 1.00 3.11 0.94 0.50 1.02 0.76 0.56 1.18 4.68 1.12

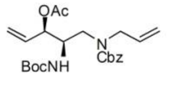

24

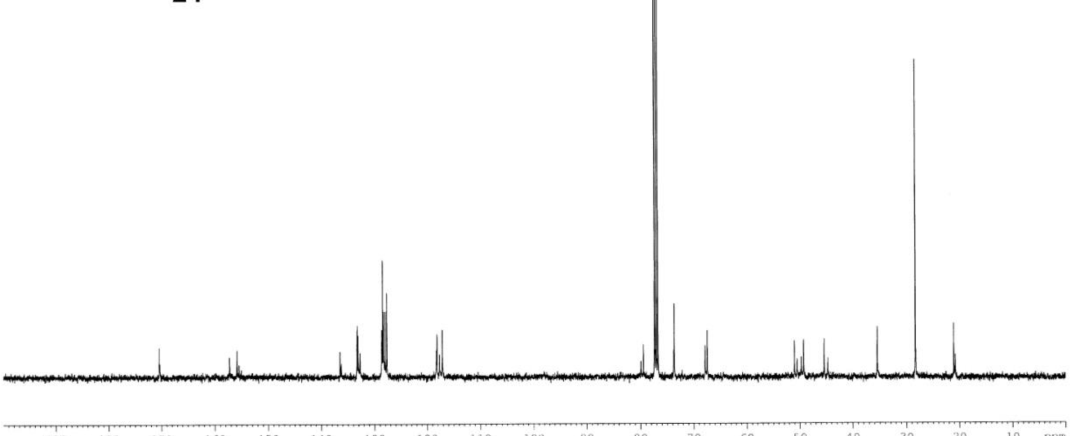
RM-2-250Lo (253)
20-3-2012
170.46 170.33 157.27 155.85 155.47 136.50 136.24 133.25 133.09 132.75 128.69 128.49 128.34 128.01 127.68 118.29 118.15 117.67 117.14 79.91 79.44 77.37 77.05 76.73 73.68 67.81 67.42 51.02 50.51 49.72 49.29 45.37 44.71 35.42 28.32 21.05 20.78
190 180 170 160 150 140 130 120 110 100 90 80 70 60 50 40 30 20 10 ppm

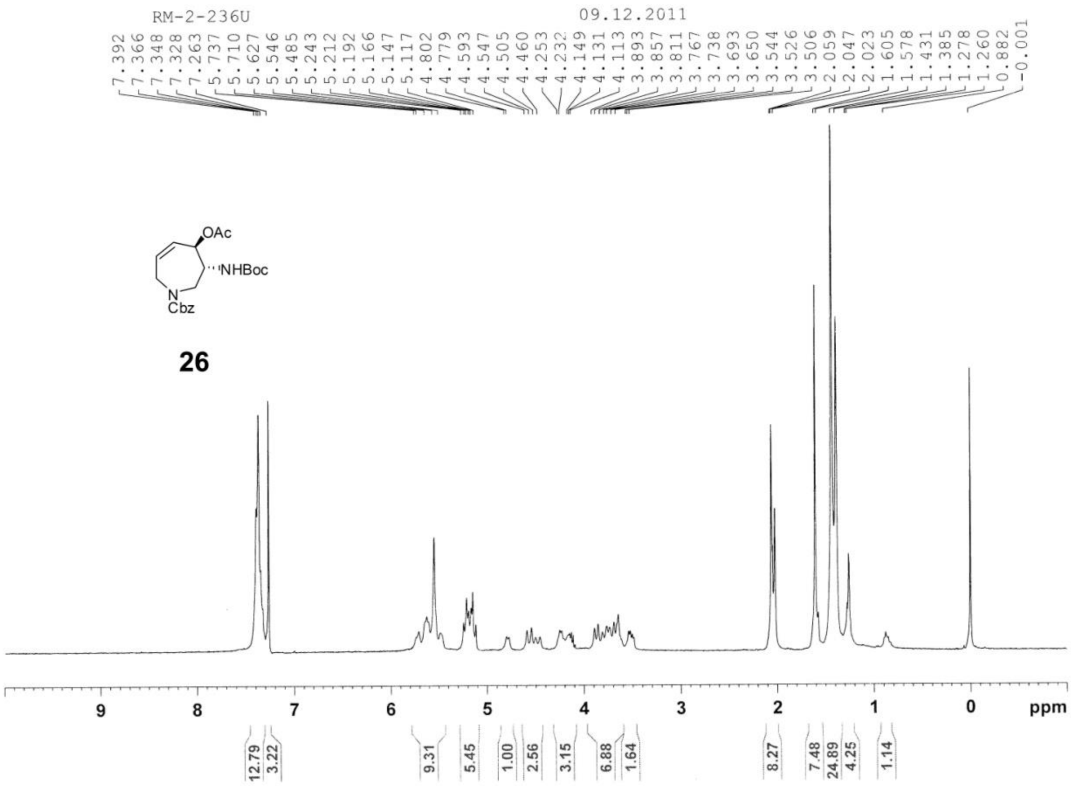
RM-2-236U
09.12.2011
7.392
7.366
7.348
7.328
7.263
5.737
5.710
5.627
5.546
5.485
5.243
5.212
5.192
5.166
5.147
5.117
4.802
4.779
4.593
4.547
4.505
4.460
4.253
4.232
4.149
4.131
4.113
3.893
3.857
3.811
3.767
3.738
3.693
3.650
3.544
3.526
3.506
2.059
2.047
2.023
1.605
1.578
1.431
1.385
1.278
1.260
0.882
-0.001
OAc
NHBoc
N
Cbz
9 8 7 6 5 4 3 2 1 0 ppm
12.79
3.22
9.31
5.45
1.00
2.56
3.15
6.88
1.64
8.27
7.48
24.89
4.25
1.14

26

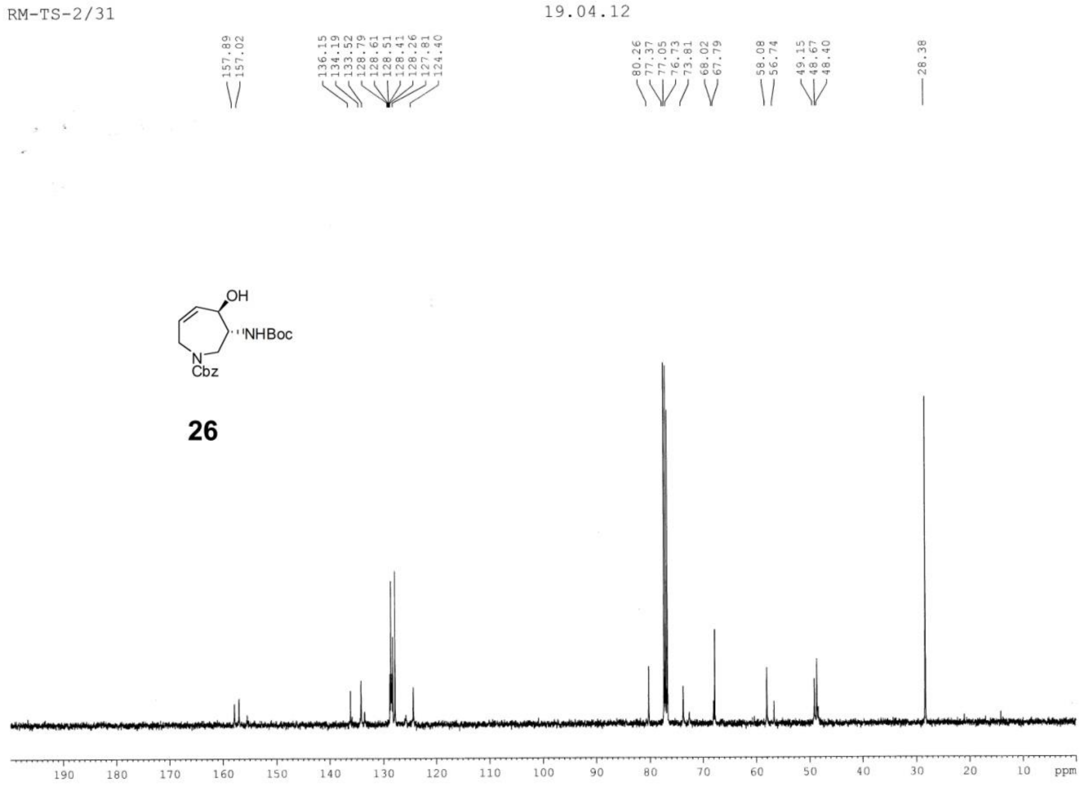
RM-TS-2/31
19.04.12
157.89
157.02
136.15
134.19
133.52
128.79
128.61
128.51
128.41
128.26
127.81
124.40
80.26
77.37
77.05
76.73
73.81
68.02
67.79
58.08
56.74
49.15
48.67
48.40
28.38
OH
NHBoc
N
Cbz
190 180 170 160 150 140 130 120 110 100 90 80 70 60 50 40 30 20 10 ppm

26

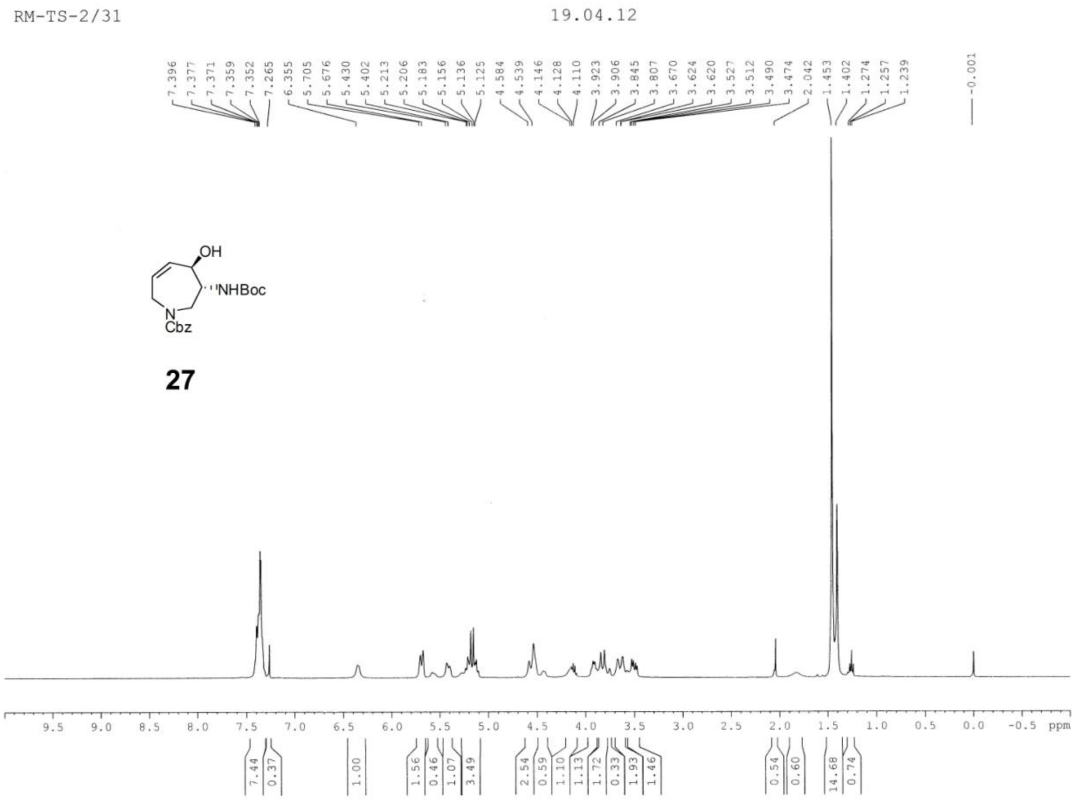
RM-TS-2/31
19.04.12
7.396 7.377 7.371 7.359 7.352 7.265 6.355 5.705 5.676 5.430 5.402 5.213 5.206 5.183 5.156 5.136 5.125 4.584 4.539 4.146 4.128 4.110 3.923 3.906 3.845 3.807 3.670 3.624 3.620 3.527 3.512 3.490 3.474 2.042 1.453 1.402 1.274 1.257 1.239 -0.001
OH
NHBoc
N
Cbz
27
9.5 9.0 8.5 8.0 7.5 7.0 6.5 6.0 5.5 5.0 4.5 4.0 3.5 3.0 2.5 2.0 1.5 1.0 0.5 0.0 -0.5 ppm
7.44 0.37 1.00 1.56 0.46 1.07 3.49 2.54 0.59 1.10 1.13 1.72 0.33 1.93 1.46 0.54 0.60 14.68 0.74

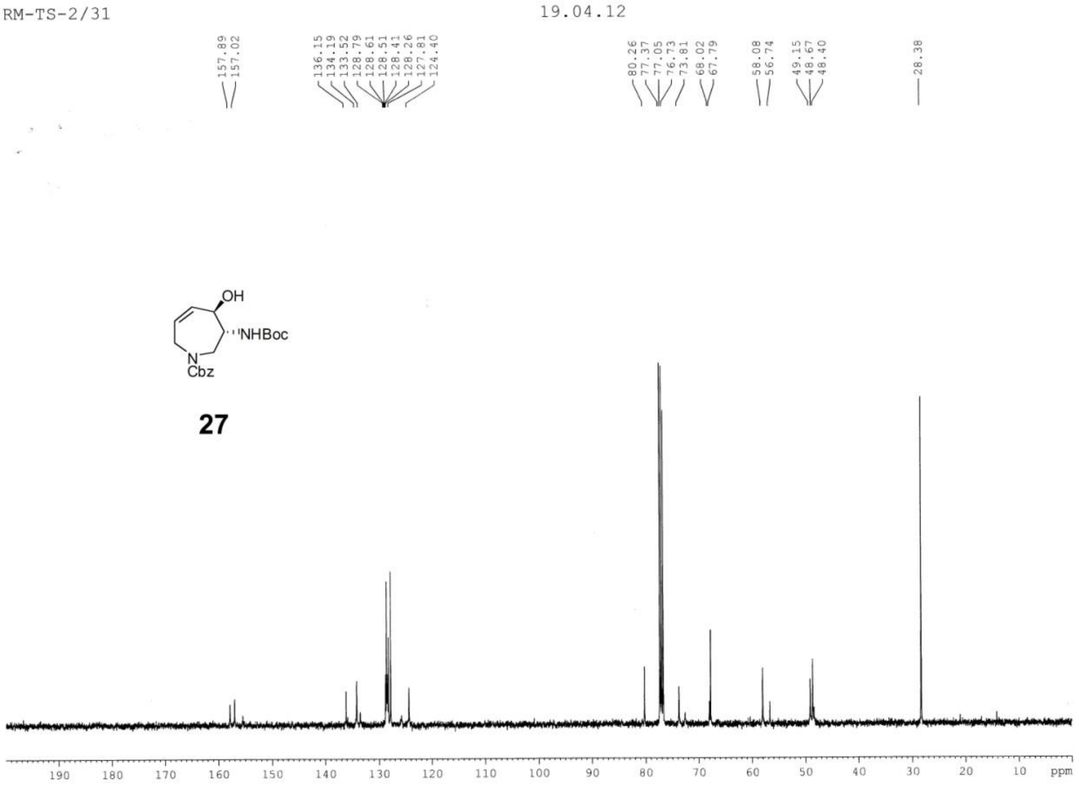
RM-TS-2/31
19.04.12
157.89 157.02 136.15 134.19 133.52 128.79 128.61 128.51 128.41 128.26 127.81 124.40 80.26 77.37 77.05 76.73 73.81 68.02 67.79 58.08 56.74 49.15 48.67 48.40 28.38
OH
NHBoc
N
Cbz
27
190 180 170 160 150 140 130 120 110 100 90 80 70 60 50 40 30 20 10 ppm

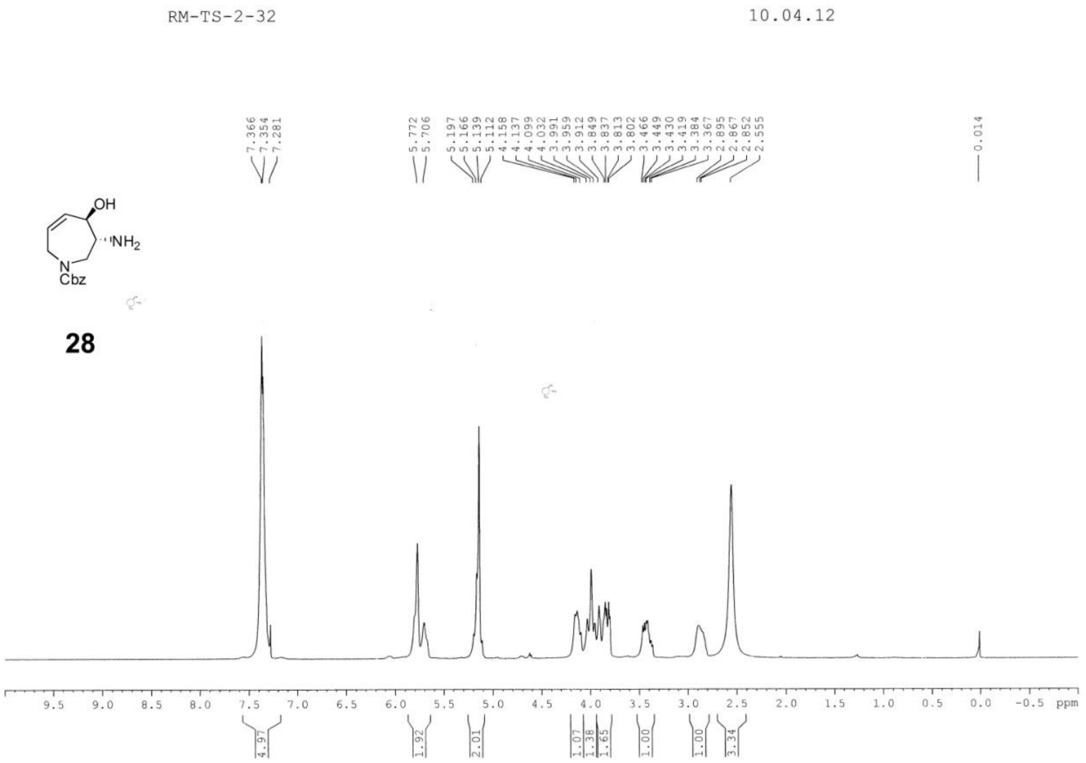

RM-TS-2-32
10.04.12
7.366
7.354
7.281
5.772
5.706
5.197
5.166
5.139
5.112
4.158
4.137
4.099
4.032
3.991
3.959
3.912
3.849
3.837
3.813
3.802
3.466
3.449
3.430
3.419
3.384
3.367
2.895
2.867
2.852
2.555
0.014
OH
NH2
N
Cbz
28
9.5
9.0
8.5
8.0
7.5
7.0
6.5
6.0
5.5
5.0
4.5
4.0
3.5
3.0
2.5
2.0
1.5
1.0
0.5
0.0
-0.5 ppm
4.97
1.92
2.01
1.07
1.38
1.65
1.00
1.00
3.34

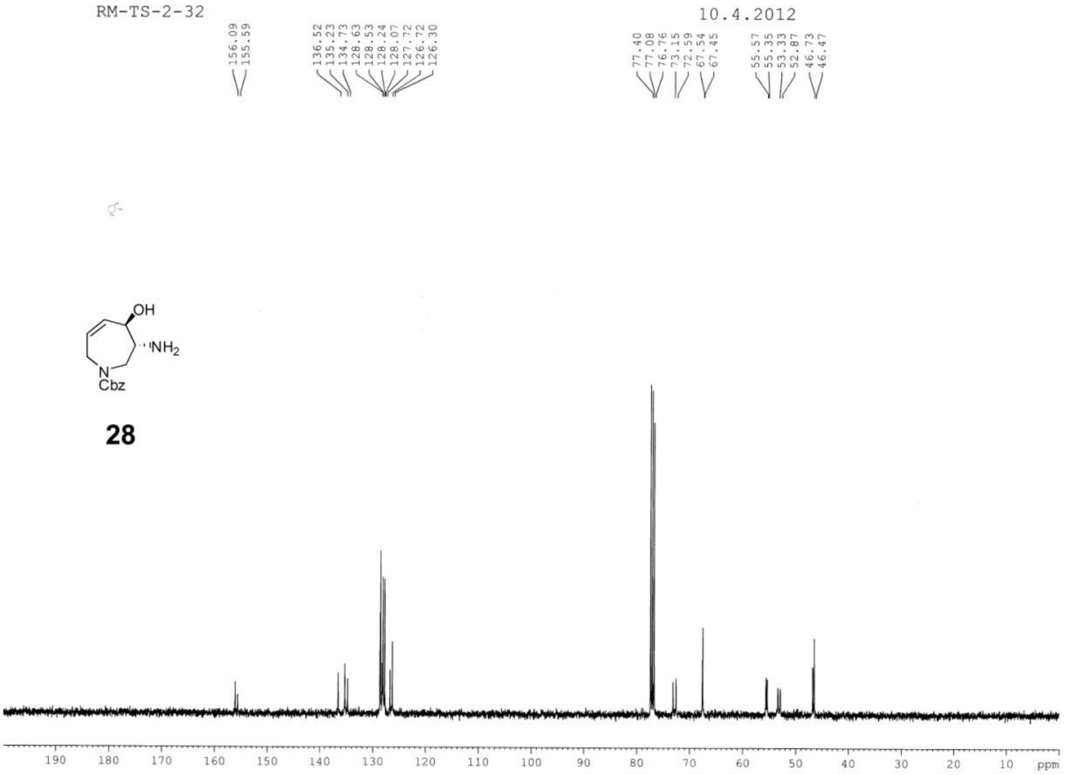

RM-TS-2-32
10.4.2012
156.09
155.59
136.52
135.23
134.73
128.63
128.53
128.24
128.07
127.72
126.30
77.40
77.08
76.76
73.15
72.59
67.54
67.45
55.57
55.35
53.33
52.87
46.73
46.47
OH
NH2
N
Cbz
28
190
180
170
160
150
140
130
120
110
100
90
80
70
60
50
40
30
20
10
ppm

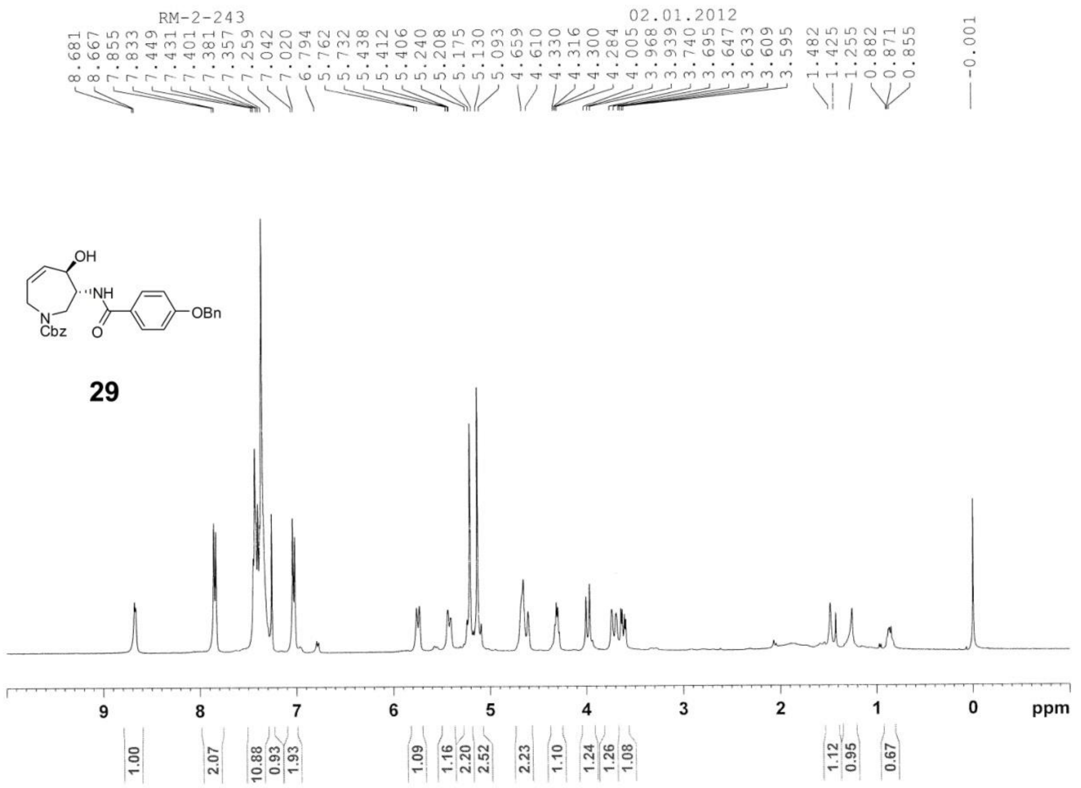
RM-2-243
02.01.2012
8.681 8.667 7.855 7.833 7.449 7.431 7.401 7.381 7.357 7.259 7.042 7.020 6.794 5.762 5.732 5.438 5.412 5.406 5.240 5.208 5.175 5.130 5.093 4.659 4.610 4.330 4.316 4.300 4.284 4.005 3.968 3.939 3.740 3.695 3.647 3.633 3.609 3.595 1.482 1.425 1.255 0.882 0.871 0.855 -0.001
OH
NH
OBn
N
Cbz
O
29
1.00 2.07 10.88 0.93 1.93 1.09 1.16 2.20 2.52 2.23 1.10 1.24 1.26 1.08 1.12 0.95 0.67
ppm

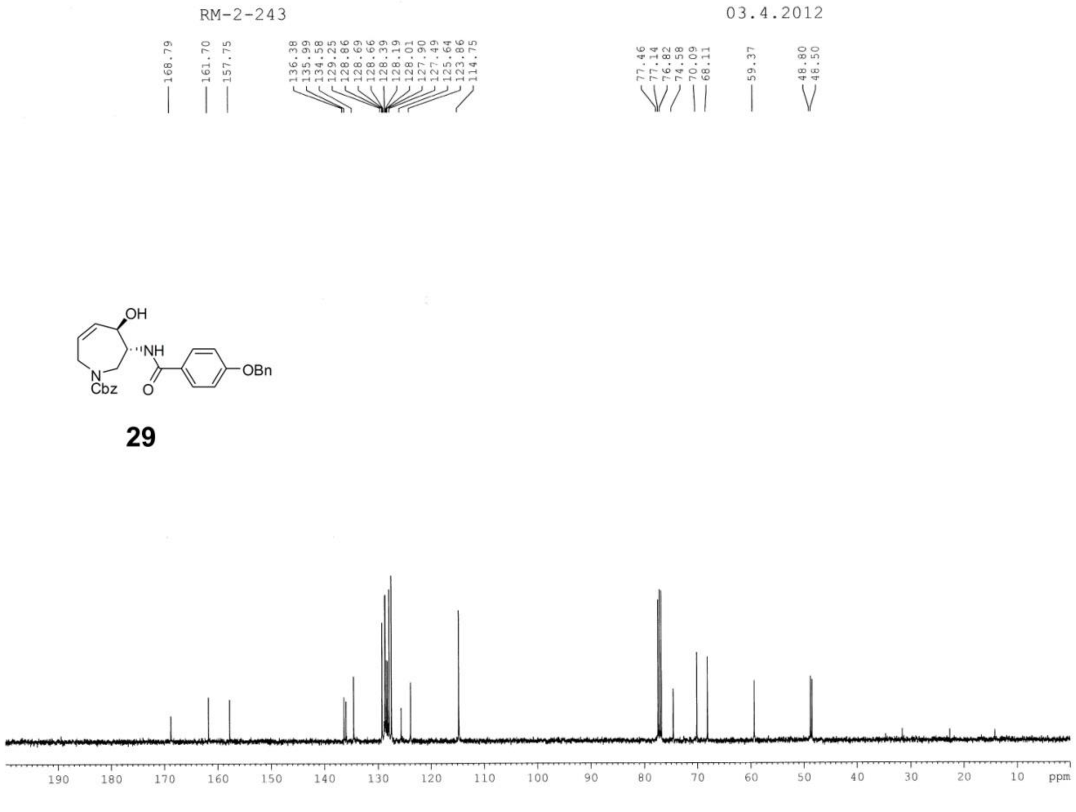
RM-2-243
03.4.2012
168.79 161.70 157.75 136.38 135.99 134.58 129.25 128.86 128.69 128.66 128.39 128.19 128.01 127.90 127.49 125.64 123.86 114.75 77.46 77.14 76.82 74.58 70.09 68.11 59.37 48.80 48.50
OH
NH
OBn
N
Cbz
O
29
ppm

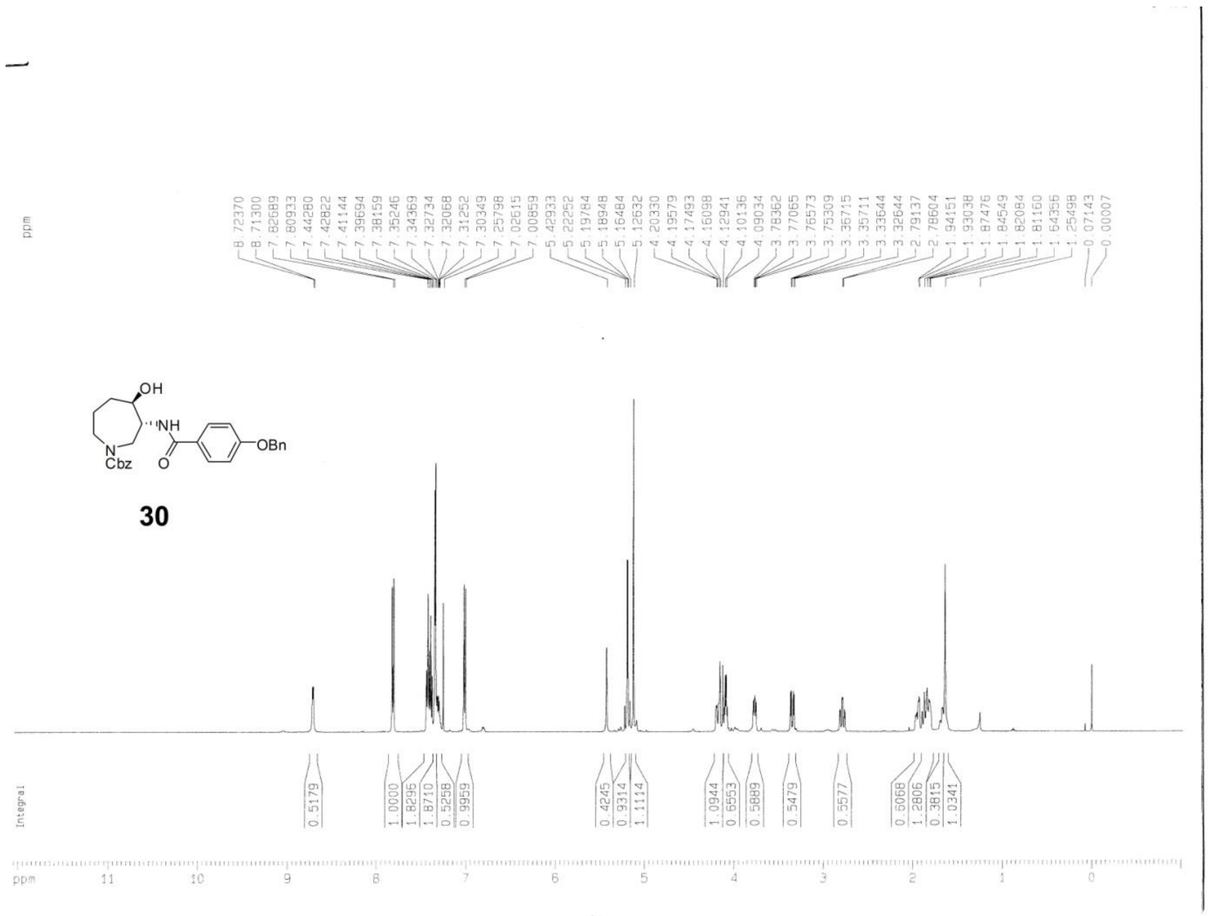
ppm
8.72370
8.71300
7.82689
7.80933
7.44280
7.42822
7.41144
7.39694
7.38159
7.35246
7.34369
7.32734
7.32068
7.31252
7.30349
7.25798
7.02615
7.00859
5.42933
5.22252
5.19784
5.18948
5.16484
5.12632
4.20330
4.19579
4.17493
4.16098
4.12941
4.10136
4.09034
3.78362
3.77065
3.76573
3.75309
3.36715
3.35711
3.33644
3.32644
2.79137
2.78604
1.94151
1.93038
1.87476
1.84549
1.82084
1.81160
1.64356
1.25498
0.07143
0.00007
OH
NH
OBn
N
Cbz
O
30
Integral
0.5179
1.0000
1.8296
1.8710
0.5258
0.9959
0.4245
0.9314
1.1114
1.0944
0.6553
0.5889
0.5479
0.5577
0.6068
1.2806
0.3815
1.0341
ppm 11 10 9 8 7 6 5 4 3 2 1 0

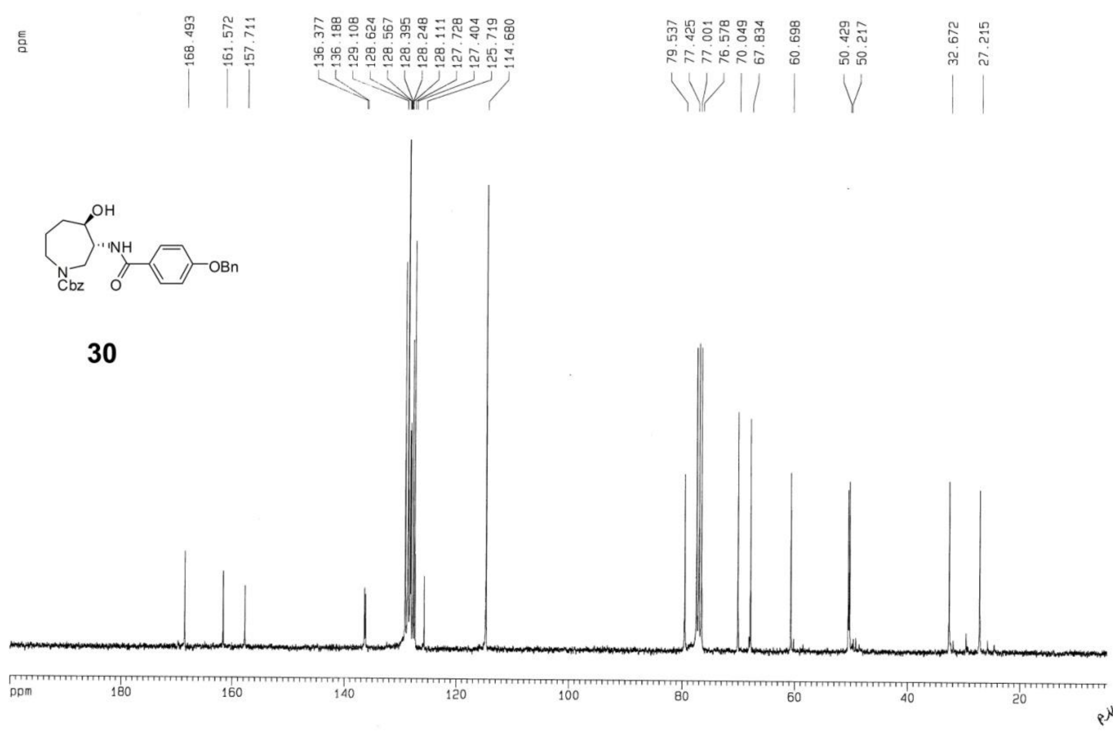
ppm
168.493
161.572
157.711
136.377
136.188
129.108
128.624
128.567
128.395
128.248
128.111
127.728
127.404
125.719
114.680
79.537
77.425
77.001
76.578
70.049
67.834
60.698
50.429
50.217
32.672
27.215
OH
NH
OBn
N
Cbz
O
30
ppm 180 160 140 120 100 80 60 40 20

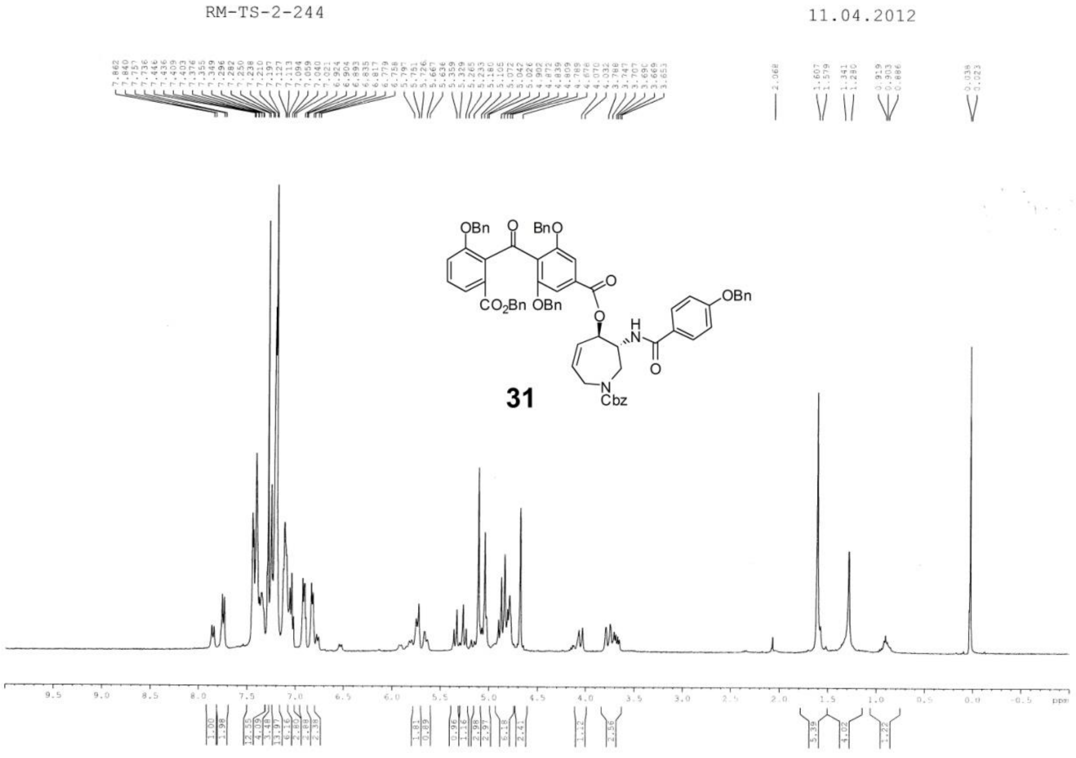
RM-TS-2-244
11.04.2012
OBn
O
BnO
CO2Bn
OBn
O
OBn
H
N
O
N
Cbz
31
ppm

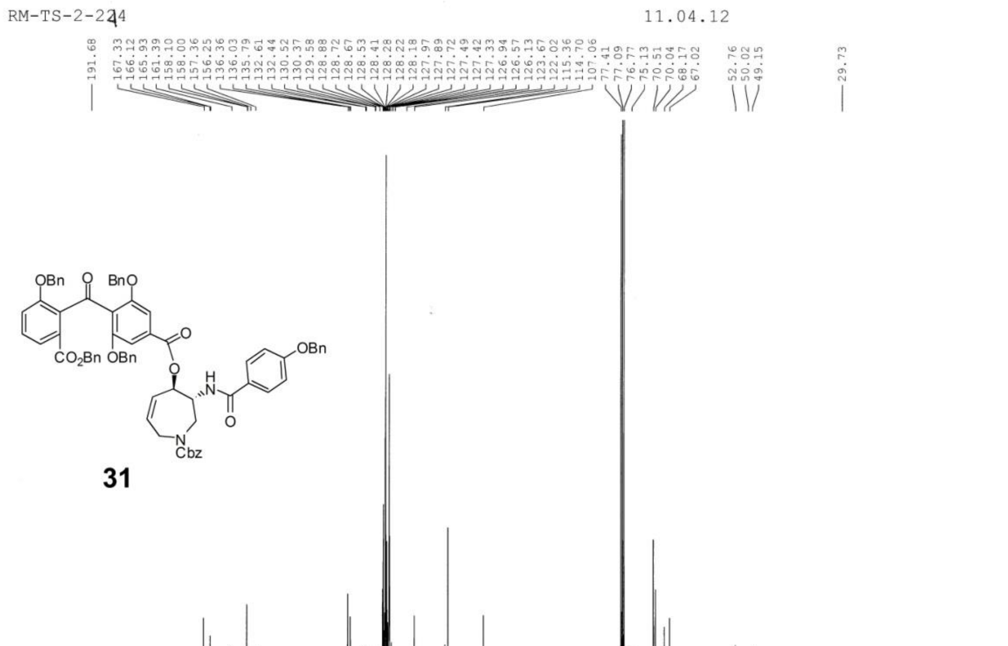
RM-TS-2-244
11.04.12
191.68
167.33
166.12
165.93
161.39
158.10
158.00
157.36
156.25
136.36
136.03
135.79
132.61
132.44
130.52
130.37
129.58
128.88
128.72
128.67
128.53
128.41
128.28
128.22
128.18
127.97
127.89
127.72
127.49
127.42
127.33
126.94
126.57
126.13
123.67
122.02
115.36
114.70
107.06
77.41
77.09
76.77
75.13
70.51
70.04
68.17
67.02
52.76
50.02
49.15
29.73
OBn
O
BnO
CO2Bn
OBn
O
OBn
H
N
O
N
Cbz
31
ppm

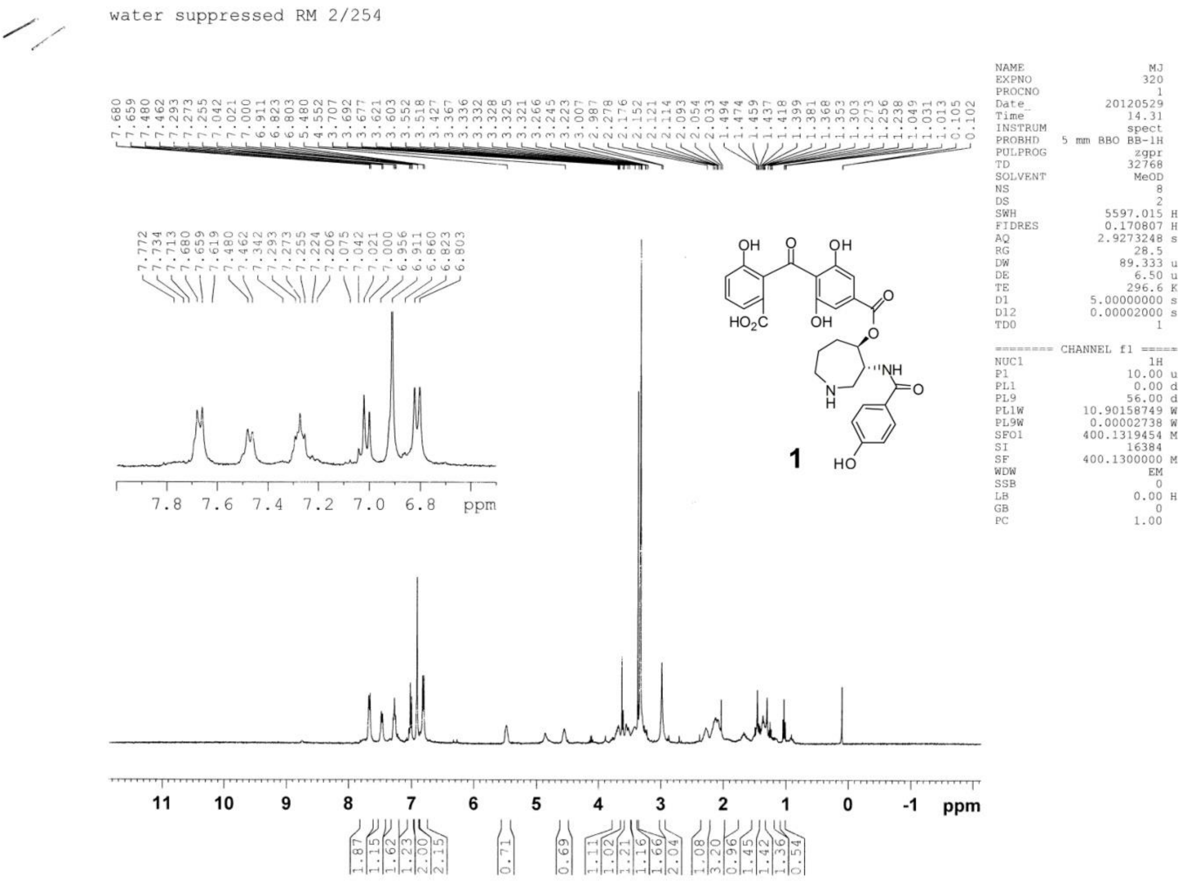

water suppressed RM 2/254
NAME MJ
EXPNO 320
PROCNO 1
Date_ 20120529
Time 14.31
INSTRUM spect
PROBHD 5 mm BBO BB-1H
PULPROG zgpr
TD 32768
SOLVENT MeOD
NS 8
DS 2
SWH 5597.015 Hz
FIDRES 0.170807 Hz
AQ 2.9273248 sec
RG 28.5
DW 89.333 usec
DE 6.50 usec
TE 296.6 K
D1 5.00000000 sec
D12 0.00002000 sec
TD0 1
CHANNEL f1
NUC1 1H
P1 10.00 usec
PL1 0.00 dB
PL9 56.00 dB
PL1W 10.90158749 W
PL9W 0.00002738 W
SFO1 400.1319454 MHz
SI 16384
SF 400.1300000 MHz
WDW EM
SSB 0
LB 0.00 Hz
GB 0
PC 1.00
1
ppm

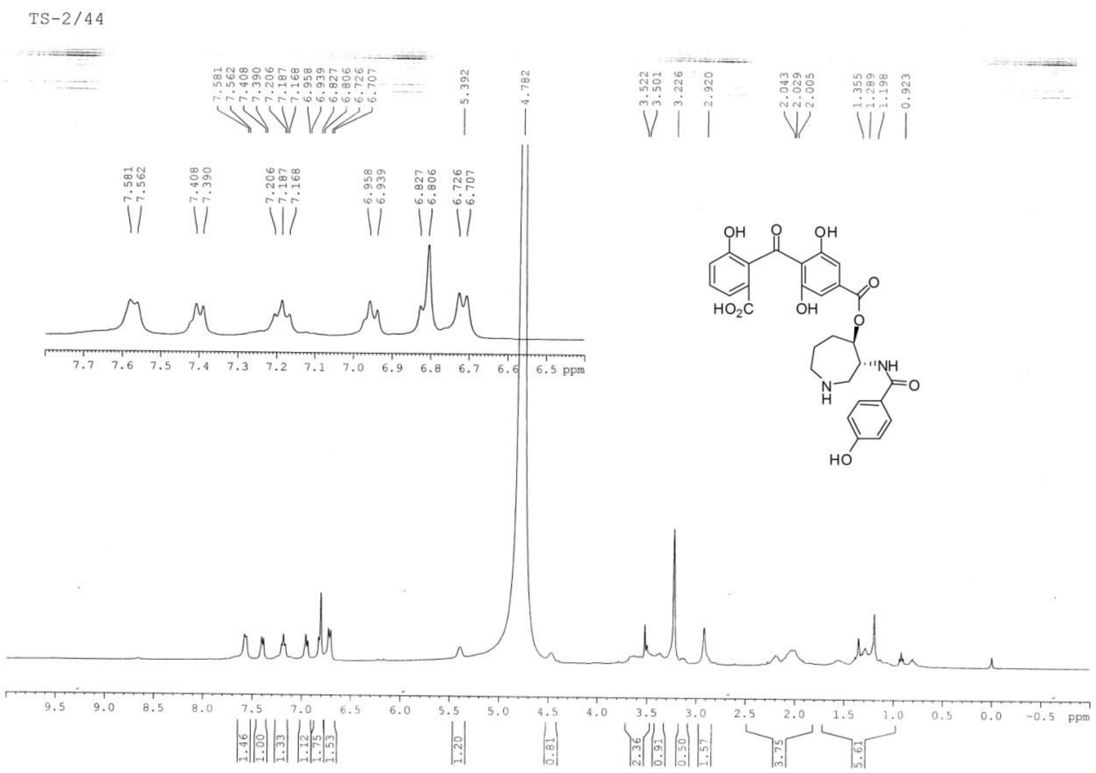
TS-2/44
7.581
7.562
7.408
7.390
7.206
7.187
7.168
6.958
6.939
6.827
6.806
6.726
6.707
5.392
4.782
3.522
3.501
3.226
2.920
2.043
2.029
2.005
1.355
1.289
1.198
0.923
ppm
OH
O
OH
HO2C
OH
O
O
NH
O
N
H
HO
1.46
1.00
1.33
1.12
1.75
1.53
1.20
0.81
2.36
0.91
0.50
1.57
3.75
5.61

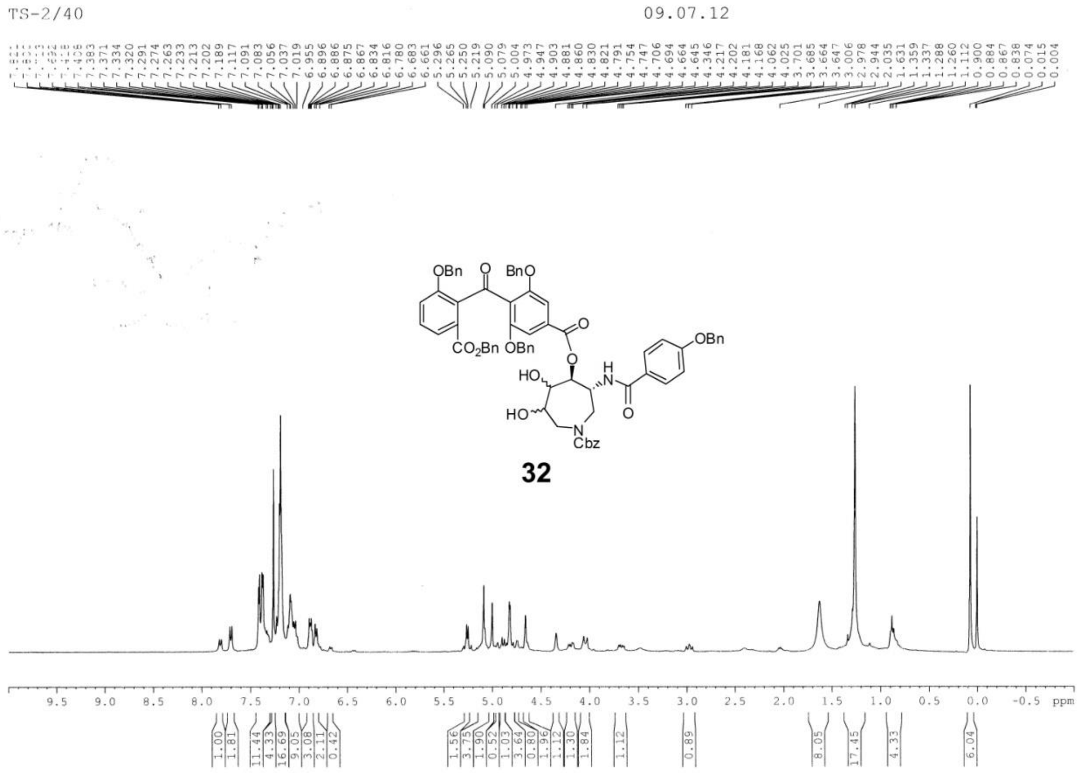
TS-2/40
09.07.12
OBn
O
BnO
CO2Bn OBn
O
OBn
HO
HO
N
H
N
O
Cbz
32
ppm

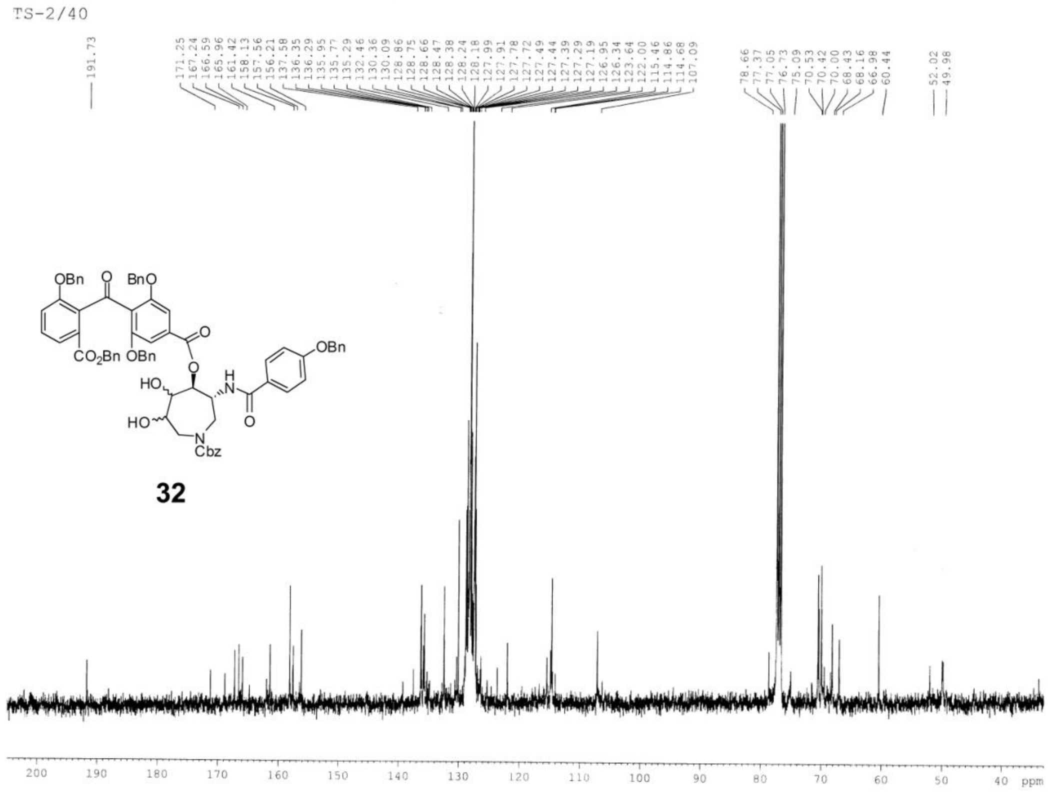
TS-2/40
191.73
OBn
O
BnO
CO2Bn OBn
O
OBn
HO
HO
N
H
N
O
Cbz
32
ppm

^{1}H NMR data of compound **30**(500 MHz, $CDCl_3$)	^{1}H NMR data of compound **30** (500 MHz, $CDCl_3$) reported by Nicolaou *et. al.*	^{13}C NMR data of compound **30**	^{13}C NMR data of compound **30** reported
8.72 (d, *J* = 5.4 Hz, 1H),	8.75 (d, *J* = 5.5 Hz,1H),	168.5	168.5
7.81 (d, *J* = 8.8 Hz, 2H),	7.82 (ddd, *J* = 8.8, 2.8, 1.9 Hz, 2H)	161.6	161.5
7.44-7.29 (m, 10H),	7.45-7.29 (m, 10H),	157.7	157.7
7.02 (d, *J* = 8.8 Hz, 2H),	7.02 (ddd, *J* =8.8, 2.8, 1.9 Hz, 2H)	136.4	136.3
5.43 (br s, 1H),<br>5.20 (AB q, *J* = 12.3 Hz, 2H),	5.21, 5.18 (AB , *J* = 12.3 Hz, 2H)	136.2	136.1
5.13 (s, 2H),	5.13 (s, 2H),	129.1	129.1
4.20 (dd, J=14.2, 3.75Hz, 1H)<br>4.16 (d, *J* = 15.8 Hz, 1H)	4.19 (dd, *J* = 14.3, 3.8 Hz,1H),<br>4.15 (d, *J* = 15.4 Hz, 1H),	128.6	128.62
		128.4	128.56
4.10- 4.06 (m, 1H),	4.10 (m, 1H),	128.2	128.2
3.76 (dd, *J* =8.9, 6.5Hz, 1H),	3.77 (ddd, *J* =10.2, 6.1, 1.6 Hz, 1H)	128.1	128.1
3.34 (dd, *J* =15.3, 5.1 Hz,1H)	3.35 (dd, *J* = 15.4, 5.1 Hz, 1H),	127.7	127.7
2.79 (dt, *J* = 3.4, 13.6Hz, 1H)	2.79 (ddd,*J* =14.3, 13.2, 3.5 Hz,1H)	127.4	127.4
1.97-1.93 (m, 1H),<br>1.90-1.81 (m, 2H),	1.95-1.81 (m, 3H),	125.7	125.6
		114.7	114.6
1.70-1.67 (m, 1H).	1.66 (m, 1H).	79.5	79.7
		70.0	70.0
		67.8	67.8
		60.7	60.7
		50.4	50.4
		50.2	50.2
		32.7	32.7
		27.2.	27.3.

^{1}H NMR(500 MHz, $CDCl_3$) data of (−)-balanol reported by Nicolaou *et. al* [2]	^{1}H NMR(500 MHz, $CDCl_3$) data of compound **1**
7.60 (d, *J* = 8.7, 2 H)	δ 7.58 (d, *J* = 8.4 Hz, 2H)
7.26 (d, *J* = 7.3 Hz, 1 H)	7.39 (d, *J* = 7.2 Hz, 1H)
7.17 (dd, *J* = 7.9, 7.9 Hz, 1H)	7.18 (t, *J* = 9.0 Hz, 1H)
6.91 (s, 2H)	6.94 (d, *J* = 8.4 Hz, 1H)
6.79 (d, *J* = 7.7Hz, 1H)	6.82 (s, 2H)
6.76 (d, *J* = 8.7 Hz, 2H)	6.71 (d, *J* = 7.6 Hz, 2H)
5.29 (m, 1H)	5.39 (m, 1H)
4.33 (br m, 1H)	4.47 (m, 1H)
3.44-2.98 (br m, 4H)	3.52-2.92 (m, 4H)
2.11-1.85 (br m, 4H)	2.19-2.02 (m, 4H)

References

1. Hollinshead, S. P.; Nichols, J. B.; Wilson, J. W. *J. Org. Chem.* **1994,** *59,* 6703–6709. doi:10.1021/jo00101a032
2. Nicolaou, K. C.; Koide, K.; Bunnage, M. E. *Chem.–Eur. J.* **1995,** *1,* 454–466. doi:10.1002/chem.19950010711
3. Nicolaou, K. C.; Bunnage, M. E.; Koide, K. *J. Am. Chem. Soc.* **1994,** *116,* 8402–8403. doi:10.1021/ja00097a072
4. Lampe, J. W.; Hughes, P. F.; Biggers, C. K.; Smith, S. H.; Hu, H. *J. Org. Chem.* **1994,** *59,* 5147–5148. doi:10.1021/jo00097a014
